# Supplementary material for: Budget impact analysis of a home-based colorectal cancer screening programme in Malaysia
Source: BMJ Open. 2023 Mar 21;13(3):e066925. doi: 10.1136/bmjopen-2022-066925 (PMC10032398; doi:10.1136/bmjopen-2022-066925)
Supplement: Supplementary data [file bmjopen-2022-066925supp001.pdf]

Supplementary material

Figure S1: Visual depiction of the budget impact cost calculator

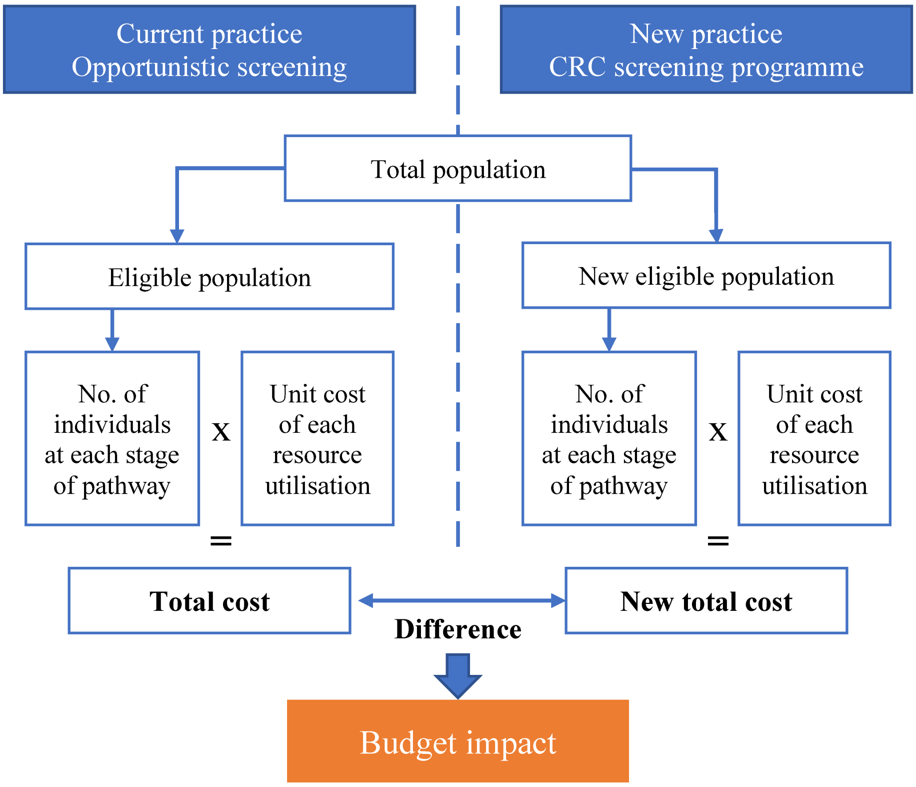

Supplementary material

Table S1: Net budget impact of CRC screening programme over 5-year timeframe, by state

Currency: Malaysian ringgit

State	Population	Year 1	Year 2	Year 3	Year 4	Year 5
**Johor**	3,822,800	12,597,041	13,791,897	14,986,752	16,181,607	17,376,462
**Kedah**	2,206,200	7,272,541	7,962,112	8,651,682	9,341,252	10,030,823
**Kelantan**	1,884,300	6,212,311	6,801,268	7,390,225	7,979,183	8,568,140
**Melaka**	934,700	3,084,637	3,376,787	3,668,937	3,961,087	4,253,238
**Negeri Sembilan**	1,141,000	3,764,122	4,120,753	4,477,384	4,834,016	5,190,647
**Pahang**	1,702,900	5,614,833	6,147,092	6,679,351	7,211,609	7,743,868
**Perak**	2,569,300	8,468,450	9,271,511	10,074,572	10,877,633	11,680,693
**Pulau Pinang**	1,767,100	5,826,301	6,378,626	6,930,951	7,483,276	8,035,601
**Sabah**	3,919,600	12,915,864	14,140,975	15,366,086	16,591,197	17,816,308
**Sarawak**	2,829,400	9,325,144	10,209,501	11,093,859	11,978,217	12,862,575
**Terengganu**	1,245,300	4,107,648	4,496,879	4,886,111	5,275,342	5,664,573
**Perlis**	253,500	841,028	920,262	999,496	1,078,730	1,157,964
**W.P. Kuala Lumpur**	1,790,100	5,902,043	6,461,557	7,021,071	7,580,585	8,140,099
**W.P. Labuan**	99,000	332,138	363,081	394,024	424,968	455,911
**W.P. Putrajaya**	97,100	325,886	356,236	386,585	416,935	447,284
**Nation-wide**	**32,676,786**	**107,631,959**	**117,845,422**	**128,058,885**	**138,272,349**	**148,485,812**

CRC: Colorectal cancer | W.P.: The Federal Territories (Malay: Wilayah Persekutuan)

Readers can convert from Malaysian Ringgit to their currency of interest (e.g., International Dollar, US Dollar, British Pound, Euro etc.) using the free web-based tool ‘CCEMG – EPPI-Centre Cost Converter’ (https://eppi.ioe.ac.uk/costconversion/default.aspx). This tool help adjusting estimates of cost expressed in one currency and price year to a specific target currency and price year.

Supplementary material

Table S2: Sensitivity of the total budget impact of CRC screening programme to changes in each variable individually

Baseline budget impact=RM107,631,959

Unit: Thousand Ringgit Malaysia

	Baseline value	Min value (-20% from baseline)	Max value (+20% from baseline)	Min budget impact	Max budget impact	Change
Probability of adults is contactable for CRC screening programme	66%	53%	79%	86,574	129,818	43,244
Probability of adults is included (eligible for CRC screening programme)	77%	62%	92%	86,386	128,950	42,564
Probability of adults agree to participate in CRC screening programme after being invited	52%	42%	63%	86,454	129,675	43,221
Probability of adults needing 1st reminder	79%	63%	94%	107,430	107,766	336
Probability of adults needing 2nd reminder	88%	70%	100%	107,535	107,643	108
Probability of adults return iFOBT result	42%	33%	50%	105,062	110,060	4,998
Probability of adults with iFOBT positive result	18%	14%	22%	105,204	109,988	4,784
Probability of adults taking colonoscopy after positive iFOBT	41%	33%	49%	105,673	109,493	3,820
Probability of adults with CRC detection after getting colonoscopy	4%	3%	5%	107,594	107,603	9
Cost to perform the screening (asking for symptoms, family history, referral)	5.58	4.47	6.70	107,633	107,567	-66
Cost of stool specimen processing	1.70	1.36	2.04	107,610	107,590	-20
Interpretation of results	2.79	2.23	3.35	107,617	107,583	-34
Cost to convey definitive diagnosis to patients (along with explaining treatment plan or referral etc.)	8.37	6.70	10.05	107,597	107,604	7
Contact eligible individuals - agreed to participate	0.98	0.79	1.18	107,285	107,926	641
Contact eligible individuals - rejected/excluded to participate	0.47	0.38	0.56	107,465	107,735	270
iFOBT rapid test kit (only the rapid test kit itself)	6.90	5.52	8.28	105,331	109,870	4,539
Print materials (instruction leaflet, explanatory statement)	1.10	0.88	1.32	107,238	107,962	724
Postage (stamp etc.)	5.35	4.28	6.42	105,840	109,360	3,520
Sending video through Whatapp	0.41	0.33	0.50	107,461	107,740	279
Sending reminder text message	0.41	0.33	0.50	107,490	107,710	220
Reminder call	0.28	0.22	0.33	107,536	107,661	125
Interpret the test kit result	1.70	1.36	2.04	107,366	107,832	466

Supplementary material

Unit: Thousand Ringgit Malaysia

	Baseline value	Min value (-20% from baseline)	Max value (+20% from baseline)	Min budget impact	Max budget impact	Change
Sending text message informing negative result	0.45	0.36	0.54	107,550	107,651	101
Call to inform positive result	0.41	0.33	0.50	107,590	107,611	21
Prepare and send referral letter	1.12	0.90	1.35	107,573	107,628	55
Follow up effort	6.73	5.39	8.08	107,503	107,698	195
Colonoscopy	200	160	240	105,638	109,564	3,926
Consumables – stool container, gloves, mask, plastic waste bag and disposal of materials from the test	10.80	8.64	12.96	93,612	121,641	28,029
