# Supplementary material for: Budget impact analysis of a home-based colorectal cancer screening programme in Malaysia
Source: BMJ Open. 2023 Mar 21;13(3):e066925. doi: 10.1136/bmjopen-2022-066925 (PMC10032398; doi:10.1136/bmjopen-2022-066925)
Supplement: Author's manuscript [file bmjopen-2022-066925.draft_revisions.pdf]

BMJ Open is committed to open peer review. As part of this commitment we make the peer review history of every article we publish publicly available.

When an article is published we post the peer reviewers' comments and the authors' responses online. We also post the versions of the paper that were used during peer review. These are the versions that the peer review comments apply to.

The versions of the paper that follow are the versions that were submitted during the peer review process. They are not the versions of record or the final published versions. They should not be cited or distributed as the published version of this manuscript.

BMJ Open is an open access journal and the full, final, typeset and author-corrected version of record of the manuscript is available on our site with no access controls, subscription charges or pay-per-view fees (http://bmjopen.bmj.com).

If you have any questions on BMJ Open's open peer review process please email info.bmjopen@bmj.com

BMJ Open

A budget impact analysis of a home-based colorectal cancer screening programme in Malaysia

Journal:	*BMJ Open*
Manuscript ID	bmjopen-2022-066925
Article Type:	Original research
Date Submitted by the Author:	28-Jul-2022
Complete List of Authors:	Ngan, Tran; Queen's University Belfast, Centre for Public Health<br>Ramanathan, Kogila; Monash University Malaysia, Global Public Health, Jeffrey Cheah School of Medicine and Health Sciences; Monash University Malaysia, South East Asia Community Observatory<br>Saleh, Muhamad; Monash University Malaysia, South East Asia Community Observatory<br>Schliemann, Desiree ; Queen's University Belfast, Centre for Public Health<br>Ibrahim Tamin, Nor Saleha ; Ministry of Health Malaysia<br>Su, Tin ; Monash University Malaysia, Global Public Health, Jeffrey Cheah School of Medicine and Health Sciences; Monash University Malaysia, South East Asia Community Observatory<br>Donnelly, Michael; Queen's University Belfast, Centre for Public Health<br>O'Neill, Ciaran; Queen's University Belfast, Centre for Public Health
Keywords:	Health economics < HEALTH SERVICES ADMINISTRATION & MANAGEMENT, Health policy < HEALTH SERVICES ADMINISTRATION & MANAGEMENT, Public health < INFECTIOUS DISEASES

SCHOLARONE™
Manuscripts

I, the Submitting Author has the right to grant and does grant on behalf of all authors of the Work (as defined in the below author licence), an exclusive licence and/or a non-exclusive licence for contributions from authors who are: i) UK Crown employees; ii) where BMJ has agreed a CC-BY licence shall apply, and/or iii) in accordance with the terms applicable for US Federal Government officers or employees acting as part of their official duties; on a worldwide, perpetual, irrevocable, royalty-free basis to BMJ Publishing Group Ltd ("BMJ") its licensees and where the relevant Journal is co-owned by BMJ to the co-owners of the Journal, to publish the Work in this journal and any other BMJ products and to exploit all rights, as set out in our licence.

The Submitting Author accepts and understands that any supply made under these terms is made by BMJ to the Submitting Author unless you are acting as an employee on behalf of your employer or a postgraduate student of an affiliated institution which is paying any applicable article publishing charge ("APC") for Open Access articles. Where the Submitting Author wishes to make the Work available on an Open Access basis (and intends to pay the relevant APC), the terms of reuse of such Open Access shall be governed by a Creative Commons licence – details of these licences and which Creative Commons licence will apply to this Work are set out in our licence referred to above.

Other than as permitted in any relevant BMJ Author's Self Archiving Policies, I confirm this Work has not been accepted for publication elsewhere, is not being considered for publication elsewhere and does not duplicate material already published. I confirm all authors consent to publication of this Work and authorise the granting of this licence.

For peer review only

A budget impact analysis of a home-based colorectal cancer screening programme in Malaysia

Tran Thu Ngan[1*], Kogila Ramanathan[2,3], Muhamad Raziq Bin Mohd Saleh[3], Désirée Schliemann[1], Nor Saleha Binti Ibrahim Tamin[4], Tin Tin Su[2,3], Michael Donnelly[1], Ciaran O'Neill[1]

[1] *Centre for Public Health, Queen's University Belfast, Belfast, UK*

[2] *Global Public Health, Jeffrey Cheah School of Medicine and Health Sciences, Monash University Malaysia, Selangor, Malaysia*

[3] *South East Asia Community Observatory (SEACO), Jeffrey Cheah School of Medicine and Health Sciences, Monash University Malaysia*

[4] *Ministry of Health Malaysia, Putrajaya, Malaysia*

* Correspondence to:

Tran Thu Ngan, PhD

Postal address: Centre for Public Health, Queen's University Belfast, Belfast, United Kingdom

Email: n.t.tran@qub.ac.uk

ORCID of the authors

Tran Thu Ngan : 0000-0003-2771-9878

Désirée Schliemann : 0000-0002-8746-3002

Ciaran O'Neill : 0000-0001-7668-3934

A budget impact analysis of a home-based colorectal cancer screening programme in Malaysia

Abstract

Objectives: The 2020-2022 research project '**C**olo**r**ectal **C**ancer **S**creening **I**ntervention for **M**alaysia' (CRC-SIM) evaluated the implementation of a home-based CRC screening pilot in Segamat District. This budget impact analysis (BIA) assessed the expected changes in health expenditure of the Malaysian Ministry of Health budget in the scenario where the pilot programme was implemented nationwide versus current opportunistic screening.

Methods: Assumptions and costs in the opportunistic and novel CRC screening scenarios were derived from a previous evaluation of opportunistic CRC screening in community health clinics across Malaysia and the CRC-SIM research project, respectively. The BIA was conducted from the viewpoint of the federal government and estimated the annual financial impact over a period of five years.

Results: The total annual cost of the current practice of opportunistic screening was RM1,584,321 of which 80% (RM1,274,690) was expended on the provision of opportunistic CRC to adults who availed of the service. Regarding the implementation of national CRC screening programme, the net budget impact in the 1st year was estimated to be RM107,631,959 and to reach RM148,485,812 in the 5th year based on an assumed increased uptake of 5% annually. The costs were calculated to be sensitive to the probability of adults who were contactable, eligible, and agreeable to participating in the programme.

Conclusions: The findings highlighted the net budget impact of implementing a population-based national CRC screening programme in Malaysia. Together with the modelling estimations, the results illustrate how a BIA may be used to improve informed decision-making by health authorities about the affordability of programme implementation as well as aid budgetary planning and decisions generally about implementation.

Keywords: Colorectal cancer screening, budget impact analysis, home-based testing, global health, Malaysia

For peer review only

Strengths and limitations of this study

- A budget impact analysis (BIA) aids decision making by health service planners and commissioners about whether an intervention or programme is affordable within given budget constraints
- BIA and its pragmatic approach is an ideal method when a situation calls for an evaluation of 'affordability' which is of central importance in low and middle income countries (LMICs)
- A BIA is not intended to provide answers to questions about whether or not the screening programme is good value for money (which can be answered by cost-effectiveness analysis)

INTRODUCTION

Colorectal cancer (CRC) has the second highest incidence and mortality rate among all types of cancer in both sexes in Malaysia.[1] The age standardised incidence rate in 2012-2016 was 14.8 per 100,000 males and 11.1 per 100,000 females which appears to be stable compared to 2007-2011.[2] In contrast, the proportion of CRC patients who are diagnosed at a late stage (i.e., stage III or IV) is increasing. The proportion of males with late stage CRC increased from 65.9% during 2007-2011 to 72.4% during 2012-2016; and from 65.2% to 73.1% for females.[2] Late stage diagnosis negatively impacts survival rate; thus, it is unsurprising that the 5-year survival of CRC patients in Malaysia is much lower compared to high-income countries (e.g., less than 50% of Malaysians compared to 92% of the population in the United States).[3 4] Improved survival can be achieved by early detection through screening and the removal of premalignant polyps.[4] However, Malaysia currently does not have a population-based national CRC screening programme.

The Ministry of Health of Malaysia (MoHM) adopted the use of immunochemical faecal occult blood test (iFOBT) for opportunistic CRC screening at public health clinics since 2014.[5] MoHM guidelines recommend screening for asymptomatic individuals aged 50-75 years old with average risk of CRC.[6] The uptake of this opportunistic screening tends to be very low. For

For peer review only - http://bmjopen.bmj.com/site/about/guidelines.xhtml

example, the annual average uptake during 2014-2018 was 0.5% while the 5-year cumulative uptake was 2.29 %.[5] Home-based iFOBT has been implemented in many high-income countries (HICs) to improve the accessibility and uptake of CRC screening.[7] In this context, the Southeast Asia Community Observatory (SEACO) at Monash University Malaysia and Queen's University Belfast (Northern Ireland) collaborated to conduct the research project, '**C**olo**r**ectal **C**ancer **S**creening **I**ntervention for **M**alaysia' (CRC-SIM) in 2020-2022. This project evaluated the implementation of a home-based CRC screening pilot in Segamat District. The uptake of the novel screening programme was 22%. The significantly higher uptake indicates the potential population wide impact if this screening approach (i.e., using home-based iFOBT and self-reporting test results) was scaled up. However, in order to aid public health decision making, there is a need to model a scaled-up version of the research-tested screening programme and, more specifically, gather insights about the total costs of programme implementation and how it might impact the MoHM budget. Therefore, this budget impact analysis (BIA) assessed the expected changes in the health expenditure of MoHM budget as a result of implementing a population-based national CRC screening programme versus current opportunistic screening (or 'usual care'). It assessed the affordability of the screening programme given potential budget constraints.

METHODS

The conduct of this BIA and presentation of this paper followed the guidelines developed by the International Society for Pharmacoeconomics and Outcomes Research (ISPOR) Task Force.[8, 9] All costs are presented in local currency -the Malaysian Ringgit (RM)- and International Dollar (I$). RM was converted to I$ using purchasing power parity (PPP) conversion factors instead of market exchange rates.

For peer review only - http://bmjopen.bmj.com/site/about/guidelines.xhtml

Health service under assessment and its comparator

The specific health service that was the focus of the BIA was a population-based screening programme for colorectal cancer using a self-rapid response iFOBT. The comparator was current or 'usual care' - opportunistic screening.

The BIA is predicated on the opportunistic screening programme being replaced by the new population-based screening programme (i.e., the two programmes would not be run in conjunction or in other words, the two scenarios in assessment are mutually exclusive). In each scenario, the patient pathway from the point when patients were invited for screening to receipt of a definitive diagnosis were identified and described. The screening procedure ends at the point of a patient receiving their iFOBT result with encouragement to attend hospital for a colonoscopy (if iFOBT is positive). It is important to note that the BIA included costs of screening and diagnosis (e.g., colonoscopy, biopsy) but not treatment. The BIA also did not address issues with respect to equity of access and uptake of services in either screening scenarios.

The patient pathways for the 'usual care' practice and the novel CRC screening programme are presented in Figure 1 and 2, respectively. In opportunistic screening practice, it is recommended or expected that individuals who are aged 50-75 years will be screened for CRC symptoms when they attend their local health clinic (for any health condition or problem). If they are asymptomatic and have an average risk of having CRC (based on family history), they are offered an iFOBT, followed by a colonoscopy if the iFOBT test was positive. If CRC is detected following a colonoscopy, the result is conveyed to a patient along with an explanation of the treatment plan or referral arrangement.

(Figure 1 is about here)

For peer review only - http://bmjopen.bmj.com/site/about/guidelines.xhtml

Details of the home-based screening intervention in CRC-SIM were published elsewhere.[10] Briefly, in the novel CRC screening programme, individuals aged 50-75 years were contacted, checked for eligibility, and invited to participate. A home-screening ‘pack’ was posted to eligible participants followed by two reminders. The test was performed at home by participants who took a photograph of the completed test and texted it to trained medical professionals who interpreted the photograph. Participants with positive iFOBT were referred for a colonoscopy at hospital.

(Figure 2 is about here)

There were two main differences between these patient pathways. Firstly, individuals within the target age group for screening were contacted directly and invited to participate in the novel CRC screening programme while in the situation of ‘usual care’, CRC screening was offered (if screening guideline recommendations were followed) only when members of the target group visited their clinic for some other health condition or problem. Secondly, the iFOBT was performed by doctors at health clinics in the ‘usual care’ pathway while in the novel CRC screening programme, participants self-tested in their home. Home-based testing generated additional stages in the pathways in relation to sending a test, reminding participants, taking a photo of a completed test, and sending it to programme officers and vice versa. The remaining stages of each pathway (e.g., being screened for eligibility, receiving a colonoscopy, and receiving a treatment plan) were the same across the two scenarios.

Eligible population and input assumptions

The target population for current opportunistic screening in Malaysia is individuals aged 50-75 years, regardless of sex. Due to the nature of home-based screening, the target population for the CRC screening programme was required to meet some additional inclusion criteria as

For peer review only

For peer review only - http://bmjopen.bmj.com/site/about/guidelines.xhtml

presented in Figure 2. The number of individuals who presented and completed each stage was estimated using input assumptions.

Data about the population of Malaysia by age was taken from government reports (i.e., Department of Statistics, Malaysia) and from World Population Review. The total population was reported to be 32,676,786 in 2021, of which, 19% or 6,228,195 were aged 50-75 years old.[11 12]

In the 'usual care' – opportunistic screening pathway or scenario, all assumptions were derived from a study by Tamin NSI (2020) which was a 5-year evaluation of opportunistic CRC screening (and the use of stool-based tests) in community health clinics across Malaysia.[5] It was assumed that 0.482% of the eligible population would avail of CRC screening when they attended local health clinics for other conditions; and 9.21% of this proportion of tested patients would receive a positive result. Only 55.9% of patients in the study by Tamin availed of a colonoscopy after a positive iFOBT. CRC detection after colonoscopy investigation was 4.04%.

In the novel CRC screening programme, all assumptions were derived from the CRC-SIM research project. It was assumed that 50.51% of the eligible population would be contactable and meet all inclusion criteria to participate in the home-based screening programme; 52.27% of people who were eligible would agree to participate; 41.63% would perform the iFOBT and send a photo of a completed test to the programme officers; 18.01% of people who would be tested would receive a positive result; 41.07% would avail of colonoscopy after a positive iFOBT result; and CRC detection after colonoscopy investigation would be 4.35%.

For peer review only - http://bmjopen.bmj.com/site/about/guidelines.xhtml

For peer review only

Table 1 summarises details about the input assumptions that were used to estimate the number of individuals at each stage of the respective pathway: the opportunistic screening pathway and the CRC screening programme pathway.

Table 1: Input assumptions used to estimate the population at each stage of the patient pathways

Stage in pathway	Opportunistic screening scenario (Current practice)		Population-based CRC programme screening scenario (Proposed practice)	
	Assumption*	No. of individuals	Assumption**	No. of individuals
Total population (all ages)	NA	32,676,786	NA	32,676,786
Target population (aged 50-75)	19.06%	6,228,195	19.06%	6,228,195
Eligible population (met all inclusion criteria)	100%	6,228,195	50.51%	3,146,020
Availed of/agreed to take CRC screening	0.482%	30,020	52.27%	1,644,561
Needed 1st reminder to return the iFOBT result (among those agreed to participate)	NA	NA	78.71%	1,294,514
Needed 2nd reminder to return the iFOBT result (among those received 1st reminder)	NA	NA	88.10%	1,140,405
Returned iFOBT result (among those agreed to participate)	100%	30,020	41.63%	684,683
Received iFOBT positive result	9.21%	2,765	18.01%	123,287
Availed of colonoscopy after positive iFOBT	55.9%	1,546	41.07%	50,636
CRC detection after colonoscopy investigation	4.04%	62	4.35%	2,202

CRC: Colorectal cancer; iFOBT: Immunochemical faecal occult blood test; NA: Not applicable; No: Number

** The assumptions were derived from a study of Tamin NSI (2020) which was a 5-year evaluation of using stool-based test for opportunistic CRC screening in primary health institutions across Malaysia [5].*

*** The assumptions were derived from the Colorectal Cancer Screening Intervention for Malaysia (or CRC-SIM research project) in Segamat District, conducted by Queen's University Belfast, Monash University, and Southeast Asia Community Observatory (SEACO) in 2021.*

For peer review only

For peer review only - http://bmjopen.bmj.com/site/about/guidelines.xhtml

Cost input and data sources

In the opportunistic screening scenario, the total cost comprised the cost of:

(i) performing screening (e.g., asking for symptoms, family history, and collecting the sample)

(ii) processing stool specimens

(iii) interpreting test results and

(iv) conveying a definitive diagnosis to patients (include explaining treatment plan or referral arrangements)

In the CRC programme screening scenario, the total cost comprised the costs of:

(i) contacting potential participants

(ii) delivering iFOBT test kits (including cost of the test, postage, print materials, and sending video instruction)

(iii) sending a reminder to participants (up to 2 times, by text message and phone call)

(iv) interpreting and conveying results to participants and

(v) following-up patients with positive iFOBT but did not take colonoscopy in order to encourage them to avail of the colonoscopy

These costs were calculated by multiplying the time allocated for the completion of each task with the salary cost of the person who undertakes each task plus cost of consumables. Table 2 shows the unit cost for each cost element, related assumptions, and data sources.

For peer review only

For peer review only - http://bmjopen.bmj.com/site/about/guidelines.xhtml

Table 2: Resources and unit costs

Currency: Malaysian ringgit (RM)

Cost element	Unit cost *(Per screen) RM (I$)*	Assumptions	Source
Current practice (opportunistic screening)			
Performing screening (asking for symptoms, family history, referral) and taking sample	5.58	20 min x salary RM2947/month	1
Processing stool specimens	1.70	10 min x salary RM1797/month	2
Interpreting the test results	2.79	10 min x salary RM2947/month	1
Conveying a definitive diagnosis to patients (along with explaining treatment plan or referral etc.)	8.37	30 min x salary RM2947/month	1
Proposed practice (Population-based CRC screening programme)			
Contact eligible individuals - agreed to participate	0.98	7.1 min x (salary RM1440/month + mobile package RM20/month)	3
Contact eligible individuals - rejected/excluded to participate	0.47	3.4 min x (same as above)	3
iFOBT rapid test kit	6.90		3
Print materials (instruction leaflet, explanatory statement)	1.10	90 cents for colour print + 20 cent for black & white print	3
Postage (stamps, etc.)	5.35		3
Sending video through WhatsApp	0.41	3 min x (salary RM1440/month + mobile package RM20/month)	3
Sending reminder text message	0.41	3 min x (same as above)	3
Reminder call	0.28	2 min x (same as above)	3
Interpreting the test kit result	1.70	10 min x salary RM1797/month	3
Sending text message to inform patient of negative result	0.45	2 min x (salary RM2350/month + mobile package RM20/month)	3
Calling patient to inform him/her of positive result	0.67	3 min x (same as above)	3
Preparing and sending referral letter to patient/clinic	1.12	5 min x (same as above)	3
Follow up effort	6.73	30 min x (same as above)	3
Developing communication materials, **one-off cost**	6,063	Communication materials do not change in 5 years	3

For peer review only - http://bmjopen.bmj.com/site/about/guidelines.xhtml

Currency: Malaysian ringgit (RM)

Cost element	Unit cost *(Per screen) RM (I$)*	Assumptions	Source
Training for data collectors*, **one-off cost**<br>* *Data collectors are those employed by the programme to (i) contact potential participants, (ii) deliver iFOBT test kits, and (iii) send a reminder to participants*	109,703	+ 1 day training (virtual using Zoom)<br>+ 1 trainer for maximum 25 trainees<br>+ 1 data collector* is needed for every target population of 400<br>+ Cost=1-day-salary of trainer/trainees x number of trainer/trainees<br>+ No retraining in 5 years	3
Same in both scenarios/practices			
Colonoscopy (including polyps removal and/or biopsy if needed)	200		
Consumables – stool container, gloves, mask, plastic waste bag and disposal of materials from the test	10.80	RM8636.7/800 sets	3

iFOBT: Immunochemical faecal occult blood test; RM: Malaysian ringgit

Source:

1. *Public Services Commission of Malaysia. Medical Officer Grade UD41. Accessed at https://www.spa.gov.my/spa/laman-utama/gaji-syarat-lantikan-deskripsi-tugas/ijazah-sarjana-phd/pegawai-perubatan-gred-ud41*
2. *Public Services Commission of Malaysia. Medical laboratory technologist Grade U29. Accessed at https://www.interactive.jpa.gov.my/ezskim/klasifikasi/perbekalanskim.asp?id_skim=3LU03*
3. *Colorectal Cancer Screening Intervention for Malaysia (or CRC-SIM research project) in Segamat District, conducted by Queen's University Belfast, Monash University, and Southeast Asia Community Observatory (SEACO) in 2021*

In the current practice of opportunistic screening, doctors were consulted about the estimated time to perform each stage in the pathway. The monthly salary of a general doctor and a medical laboratory technologist was based on the rate published by the Public Services Commission of Malaysia.[13 14] These rates were RM2,947 (~I$2,045) and RM1,797 (~I$1,247), respectively. In the novel CRC screening programme, the time to perform each stage in the pathway, salary of personnel, and costs of material resources (e.g., rapid kit test, consumables, postage, printing materials) were based on the time and expenditure observed in the CRC-SIM research project. All costs were calculated per screen except the cost of training and the cost of developing communication materials which were one-off costs based on the assumption that communication materials would not change, and no re-training would be needed within 5 years.

For peer review only - http://bmjopen.bmj.com/site/about/guidelines.xhtml

It was assumed (based on the experience of operating the screening programme during the CRC-SIM project) that one data collector (i.e., those employed by the programme to (i) contact potential participants, (ii) deliver iFOBT test kits, and (iii) send a reminder to participants) would be needed for every 400 people in the target population. Training would last one day and would be delivered virtually; thus, the cost of training equalled (1-day-salary of trainer x number of trainer) + (1-day-salary of trainees x number of trainees/data collectors).

Perspective and time horizon

The BIA was conducted from the viewpoint of the federal government which finances Malaysia's public health system.[15] Only those costs and resource requirements relevant to the budget holder were included in the analysis. For example, the out-of-pocket expenditure incurred by patients were excluded.

The analysis estimated the annual financial impact over a period of five years as recommended in the guidelines.[9 16] Costs were not discounted given that the BIA methodology reports the costs for each year in which they occur rather than a net present value.[9]

Budget impact analyses

Computing framework and base-case analysis

The BIA used a cost calculator programmed in Microsoft Excel, following the costing template[1] produced by the National Institute for Health and Care Excellence in the UK (NICE). The template was modified to fit the programme under assessment. The cost calculator approach is recommended by guidelines as it is easy for stakeholders to understand and replicate the results.[9]

---

[1] The template can be freely downloaded at https://www.nice.org.uk/Media/Default/About/what-we-do/our-programmes/evidence-standards-framework/budget-impact-template.xlsx

For peer review only - http://bmjopen.bmj.com/site/about/guidelines.xhtml

First, the number of individuals who completed each stage was estimated (Table 1). The resources that were used at each stage of the respective pathways (in opportunistic screening and the novel CRC screening programme) were listed along with their unit costs (i.e., cost of each resource per person) (Table 2). Unit costs were multiplied by number of users to give the total cost of resources for each scenario. The net budget impact was calculated as the difference in cost between opportunistic screening and the CRC screening programme. Visual depiction of the cost calculator is shown in Supplementary Material, Figure S1.

Uncertainty and scenario analyses

The input assumptions (that were used to estimate the number of individuals at each stage of the respective pathway) and the cost inputs were varied, and then the impact of these changes in relation to the results was analysed to investigate the sensitivity of the budget impact results to variations in individual input. As recommended by Gray et al. (2011), the range of variation regarding parameters for which data sources about dispersion were unavailable were ±20% of the base case.[17]

Patient and public involvement

It was not appropriate or possible to involve patients or the public in the design, or conduct, or reporting, or dissemination plans of our research as this type of study is a secondary analysis of data from a payer perspective (Ministry of Health Malaysia).

For peer review only - http://bmjopen.bmj.com/site/about/guidelines.xhtml

RESULTS

Base-case analysis

The total annual cost of the current practice of opportunistic screening is RM1,584,321 (~I$1,099,460), of which 80% (RM1,274,690 ~ I$884,587) was for providing opportunistic CRC to adults who availed of the service. Costs of providing colonoscopy (including polyps removal and/or biopsy if needed) after receipt of a positive iFOBT and conveying definitive diagnosis to patients (along with explaining treatment plan or referral etc.) after the outcome of the colonoscopy were RM309,108 (~I$214,509) and RM523 (~I$363), respectively.

The total annual cost over a 5-year period of the proposed practice (i.e., CRC screening programme) is shown in Table 3. It was assumed that the number of people who would agree to participate in the programme would increase by 5% each year (in consideration of health promotion activities as well as information flows including word of mouth between participants). Therefore, the financial impact would also increase accordingly.

For peer review only - http://bmjopen.bmj.com/site/about/guidelines.xhtml

246 **Table 3: Annual cost of proposed practice (i.e., CRC screening programme)**

Currency: Malaysian ringgit (RM) and International Dollar (I$)

Proposed practice	Year 1 RM *(I$)*	Year 2 RM *(I$)*	Year 3 RM *(I$)*	Year 4 RM *(I$)*	Year 5 RM *(I$)*
Contacting adults who are eligible for CRC screening programme (i.e., aged 50-75) and screen for eligibility of participating	2,320,148 *(1,610,096)*	2,320,148 *(1,610,096)*	2,320,148 *(1,610,096)*	2,320,148 *(1,610,096)*	2,320,148 *(1,610,096)*
Providing iFOBT test to adults who agreed to participate in CRC screening programme after being invited	93,654,886 *(64,992,981)*	102,612,907 *(71,209,512)*	111,570,928 *(77,426,043)*	120,528,949 *(83,642,574)*	129,486,970 *(89,859,105)*
Providing 1st reminder to participants	536,929 *(372,609)*	588,286 *(408,248)*	639,643 *(443,888)*	690,999 *(479,527)*	742,356 *(515,167)*
Providing 2nd reminder to participants	315,339 *(218,833)*	345,501 *(239,765)*	375,663 *(260,696)*	405,825 *(281,627)*	435,987 *(302,559)*
Interpreting returned iFOBT samples	1,165,129 *(808,556)*	1,276,572 *(885,893)*	1,388,016 *(963,231)*	1,499,460 *(1,040,569)*	1,610,903 *(1,117,906)*
Conveying result through message to participants with iFOBT negative result	251,990 *(174,872)*	276,093 *(191,598)*	300,196 *(208,324)*	324,298 *(225,050)*	348,401 *(241,777)*
Preparing and sending referral letter and calling participants with iFOBT POSITIVE result	221,356 *(153,613)*	242,529 *(168,306)*	263,701 *(182,999)*	284,874 *(197,692)*	306,046 *(212,384)*
Following up participants who DID NOT take colonoscopy after positive iFOBT	489,158 *(339,457)*	535,945 *(371,926)*	582,733 *(404,394)*	629,520 *(436,863)*	676,308 *(469,332)*
Providing colonoscopy (including polyps removal and/or biopsy if needed) to participants with positive iFOBT	10,127,147 *(7,027,861)*	11,095,801 *(7,700,070)*	12,064,455 *(8,372,280)*	13,033,109 *(9,044,489)*	14,001,764 *(9,716,700)*
Conveying definitive diagnosis to patients (along with explaining treatment plan or referral etc.) after the colonoscopy	18,432 *(12,791)*	20,195 *(14,015)*	21,958 *(15,238)*	23,721 *(16,461)*	25,484 *(17,685)*
Capital costs (Developing communication materials + Training for data collectors)	115,766 *(80,337)*	115,766 *(80,337)*	115,766 *(80,337)*	115,766 *(80,337)*	115,766 *(80,337)*
**Total cost of proposed practice**	**109,216,279** *(75,792,005)*	**119,429,743** *(82,879,766)*	**129,643,206** *(89,967,527)*	**139,856,670** *(97,055,288)*	**150,070,133** *(104,143,049)*

CRC: Colorectal cancer; iFOBT: Immunochemical faecal occult blood test

247

For peer review only - http://bmjopen.bmj.com/site/about/guidelines.xhtml

Similar to opportunistic screening, the cost to provide iFOBT to the eligible population who availed of the service accounted for 86% of the total cost of the proposed CRC screening programme. The second most costly component was the provision of colonoscopy (including polyps removal and/or biopsy if needed) to patients with an iFOBT positive result, at 9% of the total cost. The remaining nine cost components such as contacting potential participants, reminding participants to send photograph of iFOBT result, conveying diagnosis to participants and the follow-up effort added only up to 5% of the total cost.

The net budget impact in the 1st year of implementing CRC screening programme would be RM107,631,959 (~I$74,692,546 which equalled the total cost of future practice minus the total cost of current practice). The impact increases each year as the number of people who agree to participate in the programme increase, reaching RM117,845,422 (~I$81,780,307) in year 2, RM128,058,885 (~I$88,868,067) in year 3, RM138,272,349 (~I$ 95,955,829) in year 4, and RM148,485,812 (~I$103,043,589) in year 5.

The net budget impact of providing and delivering the CRC screening programme over the 5-year timeframe for each state in Malaysia (calculated according to the population size of each state) can be accessed in Supplementary Material, Table S1. These estimates aid service planning decisions if the novel pilot programme is implemented in one or more of these states before being scaled up into nationwide programme.

Uncertainty and scenario analyses

The tornado diagram in Figure 3 shows the change to net budget impact when assumptions and cost inputs were varied. It presents the results of multiple univariate sensitive analyses on key inputs that exert the most influence on the net budget impact. These inputs include the probability

For peer review only - http://bmjopen.bmj.com/site/about/guidelines.xhtml

of (i) making successful contact with adults about the CRC screening programme, (ii) adults agreeing to participate, (iii) adults being eligible to participate in the programme, and (iv) the cost of consumables that are required to take a stool sample. The first three inputs influence the number of individuals who are present at each stage of the patient pathway.

(Figure 3 is about here)

The net budget impact would increase from RM107 million to RM130 million (~I$74-90 million) if there was a 20% increase in (i) the probability of adults who were contactable (from a contact list of people aged 50-75 years old) or (ii) the probability of adults agreeing to participate in the CRC screening programme or (iii) the probability of adults being eligible for the programme (i.e., aged 50-75 years old; having no symptoms of CRC, a smartphone, and WhatsApp; resident within programme area; and did not have colonoscopy this year). In other words, a 20% increase in each one of these factors would require an additional RM23 million (~I$16 million) to be budgeted for the programme. Likewise, a 20% increase in the cost of the consumables that are required for taking stool samples would mean that the programme would cost RM15 million (~I$10 million) more than the originally calculated total cost.

DICUSSION

The result of this analysis provides information to guide public health service planners and commissioners in their decisions about an alternative CRC screening strategy i.e., a population-based CRC screening programme using home-based iFOBT compared to current opportunistic screening. It concluded that the net budget impact in the 1st year of implementing a CRC screening programme of this kind would be RM107,631,959 (~I$74,692,546). The impact would increase by year due to increase in uptake and would reach RM148,485,812 (~I$103,043,589) in the 5th year of implementation. This analytical approach and the results of this analysis are presented as aids to better decision making by MoHs and stakeholders in

For peer review only - http://bmjopen.bmj.com/site/about/guidelines.xhtml

lower-middle-income countries (LMICs) about health programme planning and in this particular illustrative case to the MoHM regarding the degree to which the proposed CRC screening programme is affordable.

The total budget that was allocated to the M0HM in 2022 was RM32.4 billion (~I$22.5 million).[18] Spending on prevention and public health services in 2009 was reported to be RM1.6 billion (~I$1.1 million).[15] More recent data and information about the size of the budget that is allocated to cancer screening is not available. As such, it is estimated that the net budget impact of implementing a CRC screening programme would account for between 7-10% of the total budget for prevention and public health services. This sum is a considerable amount of money relative to the budget allocation for prevention programmes/interventions, a budget that has to be spread across several health conditions or domains.

The key factor in the implementation of a population-based screening programme/service or the factor that has biggest impact on the budget is the size of the population who use the service. The degree of accuracy regarding population size estimates is related closely to the cost estimates in the budget. It is important for service planners to keep this point in mind and to take into account an increase in uptake and the impact of such an increase. Therefore, in the case of the CRC programme presented here, we assumed a 5% increase annually in uptake and calculated the net budget impact. The net budget impact can be recalculated according to the actual change in uptake after the programme is implemented.

Budget impact analysis is an economic assessment that is used to estimate the changes in expenditure of a specific budget holder if a new health technology/programme is implemented.[9] As such, BIA complements other health economic evaluation methods such as cost-

For peer review only - http://bmjopen.bmj.com/site/about/guidelines.xhtml

effectiveness analysis (CEA) to provide a comprehensive economic assessment of a health care intervention to decision makers.[9] A BIA aids decision making by health service planners and commissioners about whether an intervention or programme is affordable within given budget constraints while a CEA informs decisions about whether an intervention is good value for money.[9 19] BIA and its pragmatic approach is an ideal method when a situation calls for an evaluation of 'affordability' which is of central importance in LMICs and, arguably, is the key concern of whoever is in charge of managing a health care budget.[20 21]

It could well be that savings in earlier treatment would counterbalance the additional budget impact. However, a BIA is not intended to provide answers to questions about whether or not, in this context, the screening programme is good value for money as it does not take into account the potential improvements in outcomes (e.g., increase quality-related life years) and savings from lower treatment costs for CRC diagnosed at earlier stages. The conduct of other types of economic evaluations such as a cost-effectiveness analysis would be required to provide a complete and comprehensive set of evidence for decision makers.

Finally, the conduct of BIA in this paper has some limitations. First, assumptions and cost inputs for the CRC screening programme were based on the costs and rates that were observed in the CRC-SIM research project. The project was conducted in only one district (Segamat); and the distribution of three main ethnic groups (i.e., Malay, Chinese, Indian) in the project differed from the proportions that have been reported nation-wide (72%:24%:3% vs 62%:21%:6%, respectively). Therefore, it is important to be mindful of the possibility that the assumptions and inputs (based on the project) may not be representative for, or read across to, the whole population of Malaysia. Likewise, it is important to bear in mind that our findings do not include the perspective of other payers and may not generalise to other settings. The

For peer review only - http://bmjopen.bmj.com/site/about/guidelines.xhtml

results are related directly to the context of the Malaysian health system and the epidemiology of CRC in the country though they are illustrative of the positive contribution of the BIA methodology and approach.

CONCLUSIONS

This study employed a BIA methodology to analyse the costs of a novel CRC screening programme using home-based iFOBT and mHealth versus the current opportunistic screening. The findings estimated the net budget impact of implementing a population-based national CRC screening programme in Malaysia. The modelling estimations are important considerations for health authorities when they are required to decide the affordability of implementing a programme and to aid budgetary planning as well as decision making, generally, about implementation. Our study illustrates the use and value of the BIA approach in LMICs and resource-constrained settings.

For peer review only

Abbreviations

BIA	Budget impact analysis
CEA	Cost-effectiveness analysis
CRC	Colorectal cancer
CRC-SIM	Colorectal Cancer Screening Intervention for Malaysia
iFOBT	Immunochemical faecal occult blood test
I$	International Dollar
ISPOR	The Professional Society for Health Economics and Outcomes Research
MoHM	Ministry of Health of Malaysia
NICE	National Institute for Health and Care Excellence
RM	Malaysian ringgit

For peer review only - http://bmjopen.bmj.com/site/about/guidelines.xhtml

SEACO	Southeast Asia Community Observatory
UK	The United Kingdom

DECLARATIONS

Research ethics approval. Not applicable (This study does not involve human participants).

Funding. This research was supported by the Medical Research Council (UK) Global Challenges Research Fund (MR/S014349/1) and the National Medical Research Register Malaysia (NMRR ID-21-02045-O7G). The funder of the study had no role in study design, data collection, data analysis, data interpretation, or writing of the report.

Competing interests. The authors have no conflicts of interest to declare that are relevant to the content of this article.

Availability of data and material. All data generated or analysed during this study are included in this published article.

Authors' contributions. TTN: Methodology, Formal Analysis, Writing – Original draft, Writing – Review & Editing. KG, MRS, and DS: Data Curation, Investigation, Writing – Review & Editing. ST: Resources, Writing – Review & Editing. TTS, and MD: Conceptualization, Funding acquisition, Writing – Review & Editing, Supervision. CON: Methodology, Formal Analysis, Writing – Review & Editing, Supervision.

REFERENCE

1. Ferlay J, Ervik M, Lam F, et al. Global Cancer Observatory: Cancer Today. Lyon, France: International Agency for Research on Cancer, 2020.

2. Ministry of Health Malaysia. Malaysian National Cancer Registry Report 2012-2016: Ministry of Health Malaysia, 2020.

3. Magaji BA, Moy FM, Roslani AC, et al. Survival rates and predictors of survival among colorectal cancer patients in a Malaysian tertiary hospital. *BMC Cancer* 2017;17(1):339. doi: 10.1186/s12885-017-3336-z

4. Rawla P, Sunkara T, Barsouk A. Epidemiology of colorectal cancer: incidence, mortality, survival, and risk factors. *Prz Gastroenterol* 2019;14(2):89-103. doi: 10.5114/pg.2018.81072 [published Online First: 2019/01/06]

For peer review only

For peer review only - http://bmjopen.bmj.com/site/about/guidelines.xhtml

5. Tamin NSI, Razalli KA, Sallahuddin SN, et al. A 5-year evaluation of using stool-based test for opportunistic colorectal cancer screening in primary health institutions across Malaysia. *Cancer epidemiology* 2020;69:101829. doi: 10.1016/j.canep.2020.101829 [published Online First: 2020/10/01]

6. Ministry of Health Malaysia. Clinical Practice Guidelines – Management of Colorectal Carcinoma. Putrajaya: Malaysia Health Technology Assessment Section (MaHTAS), 2017:80.

7. Issa IA, Noureddine M. Colorectal cancer screening: An updated review of the available options. *World journal of gastroenterology* 2017;23(28):5086-96. doi: 10.3748/wjg.v23.i28.5086 [published Online First: 2017/08/16]

8. Mauskopf JA, Sullivan SD, Annemans L, et al. Principles of good practice for budget impact analysis: report of the ISPOR Task Force on good research practices--budget impact analysis. *Value Health* 2007;10(5):336-47. doi: 10.1111/j.1524-4733.2007.00187.x [published Online First: 2007/09/25]

9. Sullivan SD, Mauskopf JA, Augustovski F, et al. Budget impact analysis-principles of good practice: report of the ISPOR 2012 Budget Impact Analysis Good Practice II Task Force. *Value Health* 2014;17(1):5-14. doi: 10.1016/j.jval.2013.08.2291 [published Online First: 2014/01/21]

10. Ngan TT, Donnelly M, O'Neill C. Budget impact analysis of a population-based screening programme for colorectal cancer in Malaysia: technical report of a modelling study. July 1 ed. Belfast: Centre for Public Health, Queen's University Belfast, 2022:32.

11. World Population Review. Malaysia Population Pyramid 2022: World Population Review.

12. Department Statistics of Malaysia. Demographic Statistics Factsheet. Malaysia: Department Statistics of Malaysia, 2021.

13. Public Services Commission of Malaysia. Medical Officer Grade UD41: Public Services Commission of Malaysia.

14. Public Services Commission of Malaysia. Medical laboratory technologist Grade U29: Public Services Commission of Malaysia.

15. Jaafar S, Noh KM, Muttalib KA, et al. Malaysia Health System Review. In: Healy J, ed. Health Systems in Transition: Asia Pacific Observatory on Health Systems and Policies, 2013.

16. Health Information and Quality Authority. Guidelines for the budget impact analysis of health technologies in Ireland, 2018:50.

17. M.Gray A, Clarke PM, Wolstenholme JL, et al. Applied Methods of Cost-effectiveness Analysis in Health Care. New York: Oxford University Press 2011.

18. Ministry of Finance Malaysia. Budget 2022: RM32.4 billion allocation for MOH. 2021. https://www.mof.gov.my/portal/en/news/press-citations/budget-2022-rm32-4-billion-allocation-for-moh.

19. Leelahavarong P. Budget Impact Analysis. *J Med Assoc Thai* 2014;97(Suppl. 5):S65-S71.

20. Orlewska E, Gulácsi L. Budget-Impact Analyses: A Critical Review of Published Studies. *PharmacoEconomics* 2009;27(10):807-27. doi: 10.2165/11313770-000000000-00000

21. Garattini L, van de Vooren K. Budget impact analysis in economic evaluation: a proposal for a clearer definition. *The European Journal of Health Economics* 2011;12(6):499. doi: 10.1007/s10198-011-0348-5

For peer review only

For peer review only - http://bmjopen.bmj.com/site/about/guidelines.xhtml

Figure Legends and Tables

Table 1 Input assumptions used to estimate the population at each stage of the patient pathways

Table 2 Resources and unit costs

Table 3 Annual cost of proposed practice (i.e., CRC screening programme)

Figure 1 Patient pathways in 'usual care' practice - opportunistic screening for CRC

Figure 2 Patient pathway in population-based CRC screening programme

Figure 3 Results of multiple univariate sensitive analyses showing key factors that exert most influence the net budget impact

Supplementary material

Figure S1 Visual depiction of the budget impact cost calculator

Table S1 Net budget impact of CRC screening programme over 5-year timeframe, by state

For peer review only

Figure 1: Patient pathway in 'usual care' practice - opportunistic screening for CRC

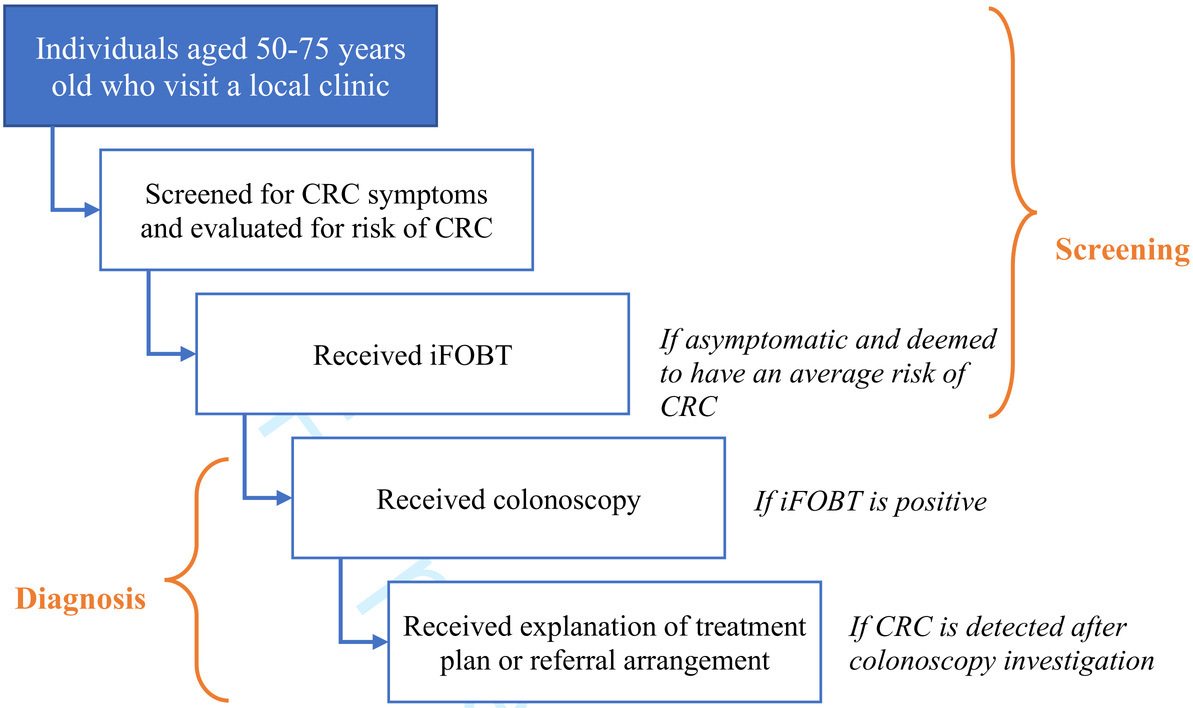

Figure 2: Patient pathway in population-based CRC screening programme

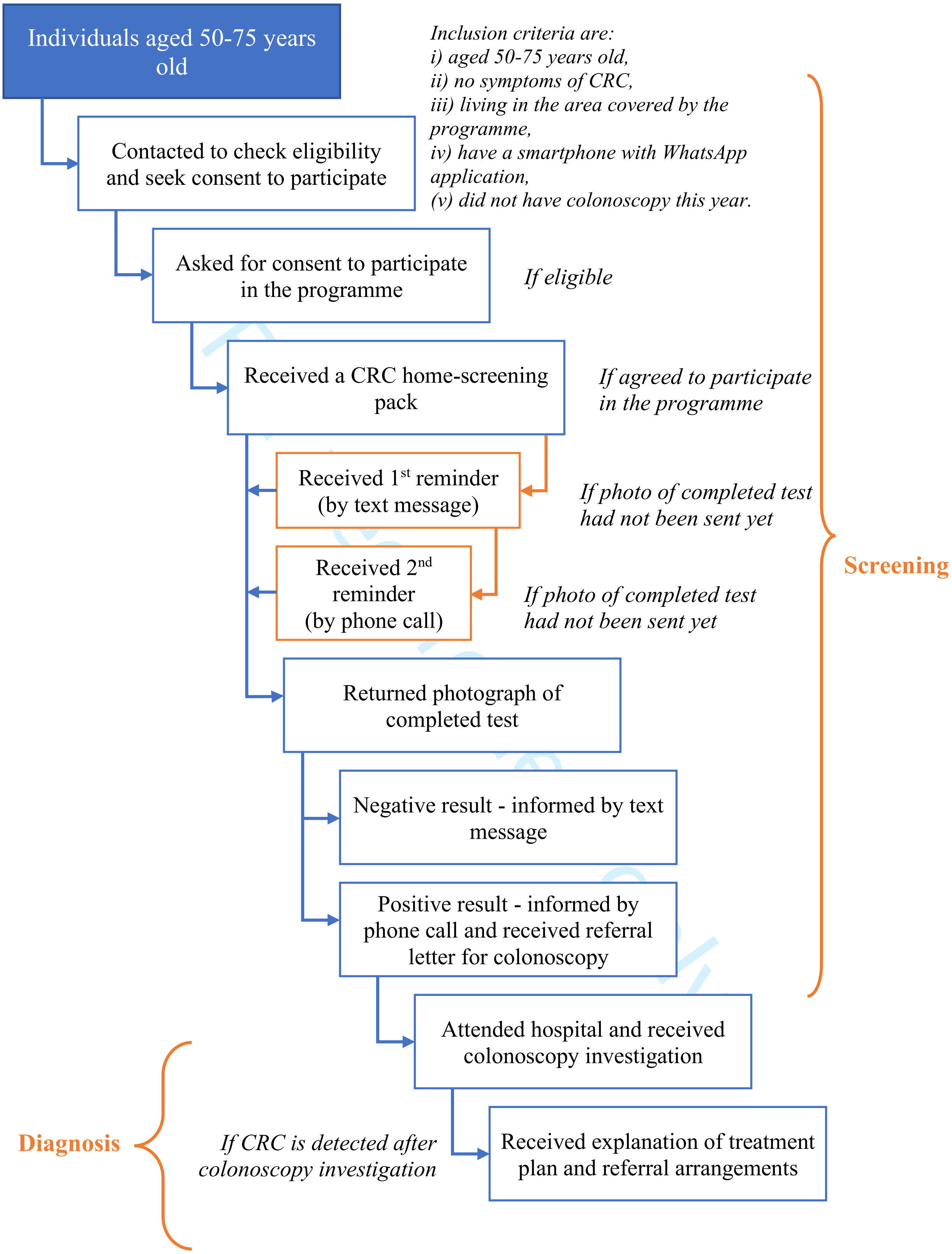

Figure 3: Results of multiple univariate sensitive analyses showing key factors that exert most influence the net budget impact

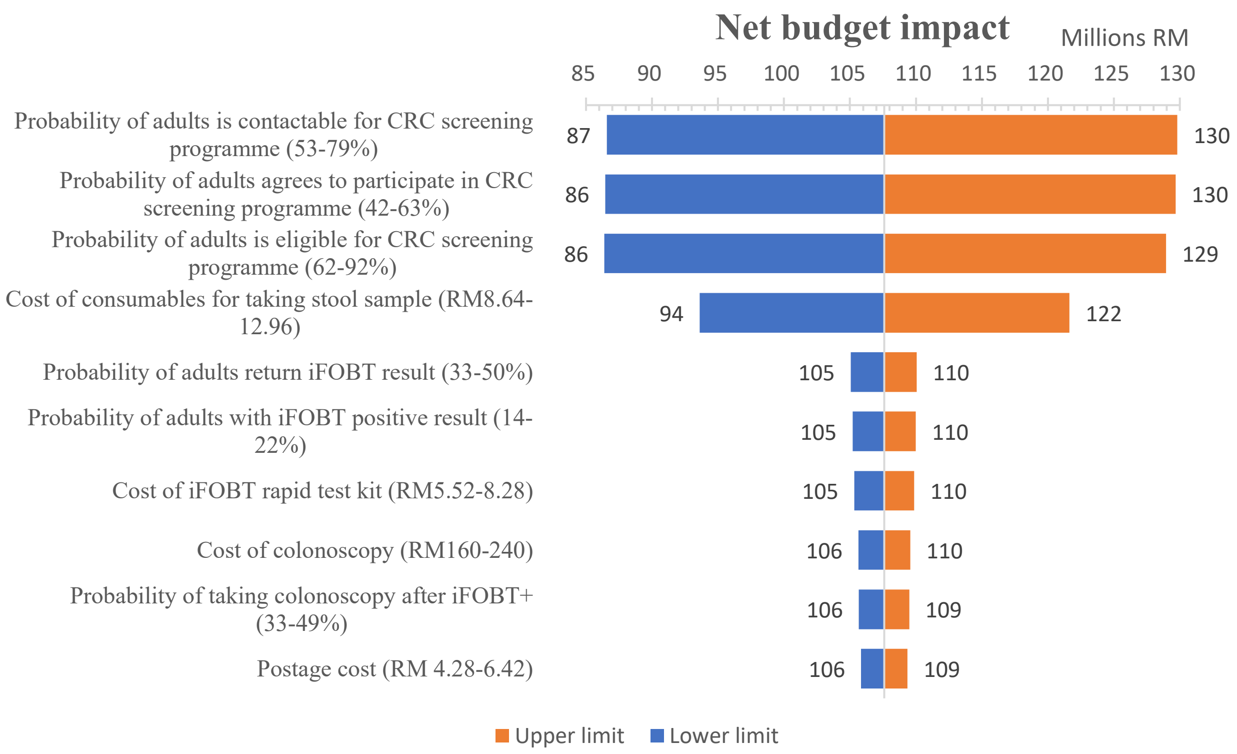

For peer review only - http://bmjopen.bmj.com/site/about/guidelines.xhtml

Supplementary material

Figure S1: Visual depiction of the budget impact cost calculator

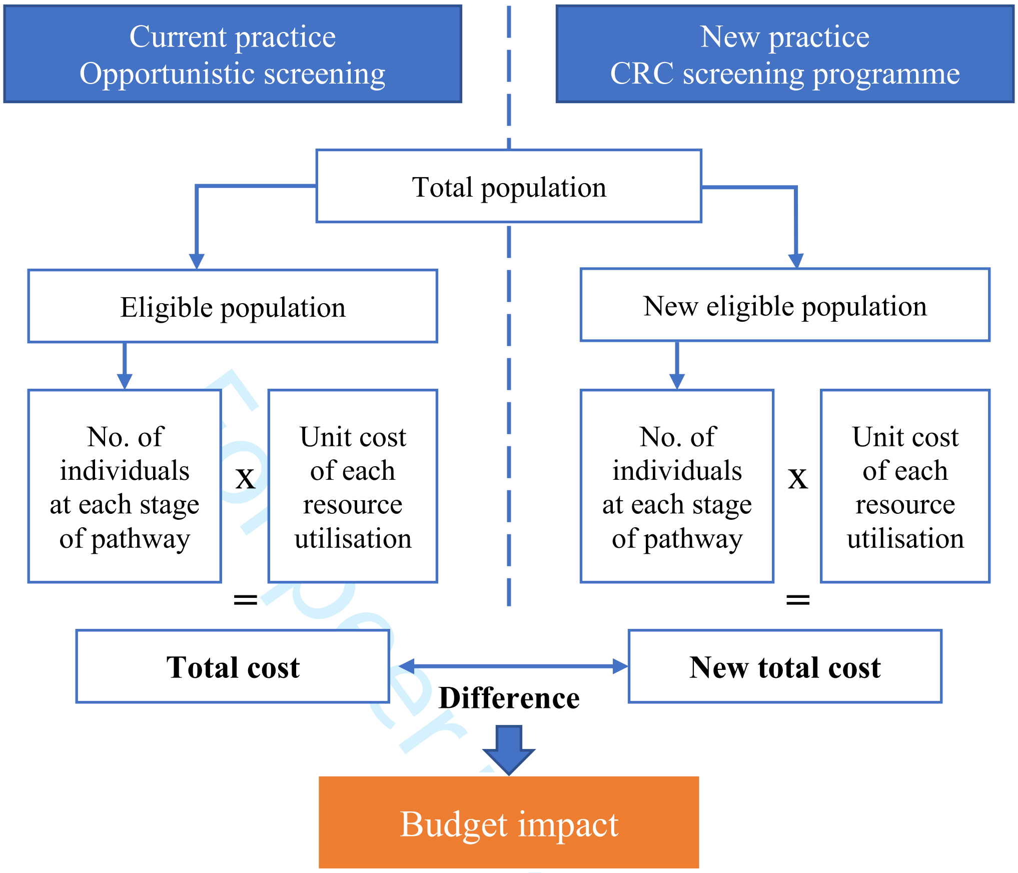

For peer review only

Supplementary material

Table S1: Net budget impact of CRC screening programme over 5-year timeframe, by state

Currency: Malaysian ringgit

State	Population	Year 1	Year 2	Year 3	Year 4	Year 5
**Johor**	3,822,800	12,597,041	13,791,897	14,986,752	16,181,607	17,376,462
**Kedah**	2,206,200	7,272,541	7,962,112	8,651,682	9,341,252	10,030,823
**Kelantan**	1,884,300	6,212,311	6,801,268	7,390,225	7,979,183	8,568,140
**Melaka**	934,700	3,084,637	3,376,787	3,668,937	3,961,087	4,253,238
**Negeri Sembilan**	1,141,000	3,764,122	4,120,753	4,477,384	4,834,016	5,190,647
**Pahang**	1,702,900	5,614,833	6,147,092	6,679,351	7,211,609	7,743,868
**Perak**	2,569,300	8,468,450	9,271,511	10,074,572	10,877,633	11,680,693
**Pulau Pinang**	1,767,100	5,826,301	6,378,626	6,930,951	7,483,276	8,035,601
**Sabah**	3,919,600	12,915,864	14,140,975	15,366,086	16,591,197	17,816,308
**Sarawak**	2,829,400	9,325,144	10,209,501	11,093,859	11,978,217	12,862,575
**Terengganu**	1,245,300	4,107,648	4,496,879	4,886,111	5,275,342	5,664,573
**Perlis**	253,500	841,028	920,262	999,496	1,078,730	1,157,964
**W.P. Kuala Lumpur**	1,790,100	5,902,043	6,461,557	7,021,071	7,580,585	8,140,099
**W.P. Labuan**	99,000	332,138	363,081	394,024	424,968	455,911
**W.P. Putrajaya**	97,100	325,886	356,236	386,585	416,935	447,284
**Nation-wide**	**32,676,786**	**107,631,959**	**117,845,422**	**128,058,885**	**138,272,349**	**148,485,812**

CRC: Colorectal cancer | W.P.: The Federal Territories (Malay: Wilayah Persekutuan)

Readers can convert from Malaysian Ringgit to their currency of interest (e.g., International Dollar, US Dollar, British Pound, Euro etc.) using the free web-based tool 'CCEMG – EPPI-Centre Cost Converter' (https://eppi.ioe.ac.uk/costconversion/default.aspx). This tool help adjusting estimates of cost expressed in one currency and price year to a specific target currency and price year.

For peer review only

ISPOR—The Professional Society for Health Economics and Outcomes Research

Budget Impact Analysis—Principles of Good Practice: Report of the ISPOR 2012 Budget Impact Analysis Good Practice II Task Force

Citation: Sullivan SD, Mauskopf JA, Augustovski F, Jaime Caro J, Lee KM, Minchin M, Orlewska E, Penna P, Rodriguez Barrios JM, Shau WY. Budget impact analysis-principles of good practice: report of the ISPOR 2012 Budget Impact Analysis Good Practice II Task Force. Value Health. 2014 Jan-Feb;17(1):5-14. doi: https://doi.org/10.1016/j.jval.2013.08.2291

Recommendations for Reporting Format

Section/topic	Guidance for reporting	Reported in section
**Introduction**		
Objectives	The objective of the BIA should be clearly stated and tied to the study perspectives	Introduction
Epidemiology and management of health problem	Present information about the prevalence and incidence of the particular disease, disease severity, disease progression, undiagnosed or undertreated cases, and risk factors pertinent to estimating the budget impact	Introduction
Clinical impact	Consist of a brief description of the eligible population and existing management options and their efficacy and safety that are relevant to the design of the study of the BIA	Introduction
Economic impact	Include a brief description of previous BIAs in the condition of interest for another intervention and condition-specific treatment patterns and cost of-care studies	Not applicable (No previous BIA)
**Study Design and Methods**		
Patient population	Specify the eligible population for the new intervention	Methods<br>Sub-section: Eligible population and input assumptions<br>Table 1
Intervention mix	Contain a detailed description of the use and characteristics of each intervention in the current intervention mix and in the expected intervention mix after the introduction of the new intervention	Methods<br>Sub-section: Health service under assessment and its comparator<br>Figure 1 and 2
Time horizon	Should be presented and the choice(s) justified	Methods<br>Sub-section: Perspective and time horizon
Perspective	Identify the BIAs' perspective(s), the cost categories included, and the intended audience	Methods<br>Perspective and time horizon

For peer review only - http://bmjopen.bmj.com/site/about/guidelines.xhtml

ISPOR—The Professional Society for Health Economics and Outcomes Research

Section/topic	Guidance for reporting	Reported in section
Analytic framework description	Complete description of the structure of the BIA cost calculator or condition-specific cohort or individual simulation model	Methods<br>Sub-section: Eligible population and input assumptions
Input data	Input values used for the reported analyses, including alternative scenarios, should be presented	Methods<br>Sub-section: Cost input and data sources<br>Table 2
Data sources	The sources of data inputs should be described in detail	Methods<br>Sub-section: Cost input and data sources<br>Table 2
Data collection	The methods and processes for any primary data collection and data abstraction tasks not reported elsewhere should be described and explained.	Not applicable (secondary data)
Analyses	A description of the calculations used to complete the BIA should be provided. The choice of all the scenarios presented in the results should be documented and justified.	Methods<br>Sub-section: Computing framework and base-case analysis under budget impact analyses
Uncertainty	Uncertainty analysis methods should be described and justified	Methods<br>Sub-sections: Uncertainty and scenario analyses under budget impact analyses
**Results**	The budget impact should be presented for each budget period over the time horizon. Both budget period resource use and costs should be presented. The estimates of resource use should be listed in a table that shows the change in use for each time period reported in the BIA<br><br>The results of the uncertainty analyses and scenarios analyzed should be described and presented in figures or tables	Results<br>Table 3<br><br>Results<br>Figure 2
**Conclusions and Limitations**	State the main conclusions on the basis of the results of the BIA.<br>Report the main limitations regarding key issues such as design aspects including off-label use and adherence assumptions and the completeness and quality of data inputs and sources.	Discussion<br><br>Conclusion
**Inclusion of Graphics and Tables**		
Figure of the analytical framework	Flow diagrams or other visual depictions of the cost calculator or condition-specific cohort or individual simulation model are recommended to be included with the analytical framework description.	Supplementary material<br>Figure S1

For peer review only - http://bmjopen.bmj.com/site/about/guidelines.xhtml

ISPOR—The Professional Society for Health Economics and Outcomes Research

Section/topic	Guidance for reporting	Reported in section
Table of assumptions	All the major assumptions should be listed in a tabular form	Table 1
Tables of inputs	All the input parameter values and their data sources and<br>derivations should be presented in a tabular form	Table 2
Tables of outputs	All outputs should be presented in a tabular and/or graphical<br>Form	Table 3
Schematic representation of uncertainty analyses	Diagrams such as Tornado diagrams should be included along with the text on the results of the scenario analyses	Figure 3
Appendices and References	The appendices may cover literature search strategies, evidence summaries, intermediate results (e.g., of individual Delphi panel rounds), and the names and addresses of participating experts and investigators, for example.	Reference

For peer review only

BMJ Open

A budget impact analysis of a home-based colorectal cancer screening programme in Malaysia

Journal:	*BMJ Open*
Manuscript ID	bmjopen-2022-066925.R1
Article Type:	Original research
Date Submitted by the Author:	21-Dec-2022
Complete List of Authors:	Ngan, Tran; Queen's University Belfast, Centre for Public Health<br>Ramanathan, Kogila; Monash University Malaysia, Global Public Health, Jeffrey Cheah School of Medicine and Health Sciences; Monash University Malaysia, South East Asia Community Observatory<br>Saleh, Muhamad; Monash University Malaysia, South East Asia Community Observatory<br>Schliemann, Desiree ; Queen's University Belfast, Centre for Public Health<br>Ibrahim Tamin, Nor Saleha ; Ministry of Health Malaysia<br>Su, Tin ; Monash University Malaysia, Global Public Health, Jeffrey Cheah School of Medicine and Health Sciences; Monash University Malaysia, South East Asia Community Observatory<br>Donnelly, Michael; Queen's University Belfast, Centre for Public Health<br>O'Neill, Ciaran; Queen's University Belfast, Centre for Public Health
**Primary Subject Heading**:	Health economics
Secondary Subject Heading:	Global health, Public health, Oncology
Keywords:	Health policy < HEALTH SERVICES ADMINISTRATION & MANAGEMENT, ONCOLOGY, HEALTH ECONOMICS, PUBLIC HEALTH

SCHOLARONE™
Manuscripts

I, the Submitting Author has the right to grant and does grant on behalf of all authors of the Work (as defined in the below author licence), an exclusive licence and/or a non-exclusive licence for contributions from authors who are: i) UK Crown employees; ii) where BMJ has agreed a CC-BY licence shall apply, and/or iii) in accordance with the terms applicable for US Federal Government officers or employees acting as part of their official duties; on a worldwide, perpetual, irrevocable, royalty-free basis to BMJ Publishing Group Ltd ("BMJ") its licensees and where the relevant Journal is co-owned by BMJ to the co-owners of the Journal, to publish the Work in this journal and any other BMJ products and to exploit all rights, as set out in our licence.

The Submitting Author accepts and understands that any supply made under these terms is made by BMJ to the Submitting Author unless you are acting as an employee on behalf of your employer or a postgraduate student of an affiliated institution which is paying any applicable article publishing charge ("APC") for Open Access articles. Where the Submitting Author wishes to make the Work available on an Open Access basis (and intends to pay the relevant APC), the terms of reuse of such Open Access shall be governed by a Creative Commons licence – details of these licences and which Creative Commons licence will apply to this Work are set out in our licence referred to above.

Other than as permitted in any relevant BMJ Author's Self Archiving Policies, I confirm this Work has not been accepted for publication elsewhere, is not being considered for publication elsewhere and does not duplicate material already published. I confirm all authors consent to publication of this Work and authorise the granting of this licence.

For peer review only

A budget impact analysis of a home-based colorectal cancer screening programme in Malaysia

Tran Thu Ngan[1*], Kogila Ramanathan[2,3], Muhamad Raziq Bin Mohd Saleh[3], Désirée Schliemann[1], Nor Saleha Binti Ibrahim Tamin[4], Tin Tin Su[2,3], Michael Donnelly[1], Ciaran O'Neill[1]

[1] *Centre for Public Health, Queen's University Belfast, Belfast, UK*

[2] *Global Public Health, Jeffrey Cheah School of Medicine and Health Sciences, Monash University Malaysia, Selangor, Malaysia*

[3] *South East Asia Community Observatory (SEACO), Jeffrey Cheah School of Medicine and Health Sciences, Monash University Malaysia*

[4] *Ministry of Health Malaysia, Putrajaya, Malaysia*

* Correspondence to:

Tran Thu Ngan, PhD

Postal address: Centre for Public Health, Queen's University Belfast, Belfast, United Kingdom

Email: n.t.tran@qub.ac.uk

ORCID of the authors

Tran Thu Ngan : 0000-0003-2771-9878

Désirée Schliemann : 0000-0002-8746-3002

Ciaran O'Neill : 0000-0001-7668-3934

For peer review only

A budget impact analysis of a home-based colorectal cancer screening programme in Malaysia

Abstract

Objectives: The 2020-2022 research project '**Colo**rectal **C**ancer **S**creening **I**ntervention for **M**alaysia' (CRC-SIM) evaluated the implementation of a home-based CRC screening pilot in Segamat District. This budget impact analysis (BIA) assessed the expected changes in health expenditure of the Malaysian Ministry of Health budget in the scenario where the pilot programme was implemented nationwide versus current opportunistic screening.

Design: Budget impact analysis. Assumptions and costs in the opportunistic and novel CRC screening scenarios were derived from a previous evaluation of opportunistic CRC screening in community health clinics across Malaysia and the CRC-SIM research project, respectively.

Setting: National level (with supplement analysis for district level). The BIA was conducted from the viewpoint of the federal government and estimated the annual financial impact over a period of five years.

Results: The total annual cost of the current practice of opportunistic screening was RM1,584,321 (~I$1,099,460; RM=Ringgit Malaysia; I$=International dollar) of which 80% (RM1,274,690 or ~I$884,587) was expended on the provision of opportunistic CRC to adults who availed of the service. Regarding the implementation of national CRC screening programme, the net budget impact in the 1st year was estimated to be RM107,631,959 (~I$74,692,546) and to reach RM148,485,812 (~I$103,043,589) in the 5th year based on an assumed increased uptake of 5% annually. The costs were calculated to be sensitive to the probability of adults who were contactable, eligible, and agreeable to participating in the programme.

Conclusions: Results from the BIA aids decision making by health services planners and commissioners in Malaysia about whether a population-based national CRC screening programme is affordable within given budget constraint. The study also illustrates the use and value of the BIA approach in LMICs and resource-constrained settings.

Keywords: Colorectal cancer screening, budget impact analysis, home-based testing, global health, Malaysia

For peer review only - http://bmjopen.bmj.com/site/about/guidelines.xhtml

Strengths and limitations of this study

- The budget impact analysis (BIA) was used to evaluate the 'affordability' of colorectal cancer (CRC) screening programme in Malaysia within given budget constraint.
- Assumptions and cost inputs for modelling the budget impact were based on the actual costs and rates observed in Malaysia.
- The total cost of resources (=unit costs * number of users) for opportunistic screening and the CRC screening programme were compared to calculate the net budget impact.
- The BIA was conducted from the viewpoint of the federal government and only included costs and resource requirements relevant to this particular budget holder.
- The BIA could not and was not intended to provide answers to questions about whether or not the screening programme is good value for money (which can be answered by a cost-effectiveness analysis).

INTRODUCTION

Colorectal cancer (CRC) has the second highest incidence and mortality rate among all types of cancer in both sexes in Malaysia [1]. The age standardised incidence rate in 2012-2016 was 14.8 per 100,000 males and 11.1 per 100,000 females which appears to be stable compared to 2007-2011 [2]. In contrast, the proportion of CRC patients who are diagnosed at a late stage (i.e., stage III or IV) is increasing. Report from Ministry of Health Malaysia (MoHM) showed that the proportion of males with late stage CRC increased from 65.9% during 2007-2011 to 72.4% during 2012-2016; and from 65.2% to 73.1% for females [2]. The report did not give an explanation about this increasing trend though [2]. Late stage diagnosis negatively impacts survival rate; for example, the 5-year survival rates for cases diagnosed at stage I, II, III, and IV in 2002-2004 in Kuala Lumpur were 78.6%, 52.9%, 44.3%, and 9.3%, respectively [3]. Improved survival can be achieved by early detection through screening and the removal of premalignant polyps [4]. However, Malaysia currently does not have a population-based national CRC screening programme.

For peer review only - http://bmjopen.bmj.com/site/about/guidelines.xhtml

The Ministry of Health of Malaysia (MoHM) adopted the use of immunochemical faecal occult blood test (iFOBT) for opportunistic CRC screening at public health clinics since 2014 [5]. MoHM guidelines recommend screening for asymptomatic individuals aged 50-75 years old with average risk of CRC [6]. The uptake (number of patients screened/total eligible population) of this opportunistic screening tends to be very low. The annual average uptake during 2014-2018 was 0.5% while the 5-year cumulative uptake was 2.29% due to low awareness about CRC in general and CRC tests in particular, fear of the result, concern about the cost, and absence of a doctor's recommendation [5, 7]. Home-based iFOBT has been implemented in many high-income countries (HICs) to improve the accessibility and uptake of CRC screening [8]. In this context, the Southeast Asia Community Observatory (SEACO) at Monash University Malaysia and Queen's University Belfast (Northern Ireland) collaborated to conduct the research project, '**Colo**r**e**ctal **C**ancer **S**creening **I**ntervention for **M**alaysia' (CRC-SIM) in 2020-2022. This project evaluated the implementation of a home-based CRC screening pilot in Segamat District. The uptake of the novel screening programme was 22%. The significantly higher uptake indicates the potential population wide impact if this screening approach (i.e., using home-based iFOBT and self-reporting test results) was scaled up. However, in order to aid public health decision making, there is a need to model a scaled-up version of the research-tested screening programme and, more specifically, gather insights about the total costs of programme implementation and how it might impact the MoHM budget. Therefore, this budget impact analysis (BIA) assessed the expected changes in the health expenditure of MoHM budget as a result of implementing a population-based national CRC screening programme versus current opportunistic screening (or 'usual care'). It assessed the affordability of the screening programme given potential budget constraints.

For peer review only - http://bmjopen.bmj.com/site/about/guidelines.xhtml

METHODS

The conduct of this BIA and presentation of this paper followed the guidelines developed by the International Society for Pharmacoeconomics and Outcomes Research (ISPOR) Task Force [9, 10]. All costs are presented in local currency -the Malaysian Ringgit (RM)- and International Dollar (I$). RM was converted to I$ using purchasing power parity (PPP) conversion factors instead of market exchange rates. The PPP conversion rate of 1.441 was obtained from the IMF World Economic Outlook Database [11].

Health service under assessment and its comparator

The specific health service that was the focus of the BIA was a population-based screening programme for colorectal cancer using a self-rapid response iFOBT. The comparator was current or 'usual care' - opportunistic screening.

The BIA is predicated on the opportunistic screening programme being replaced by the new population-based screening programme (i.e., the two programmes would not be run in conjunction or in other words, the two scenarios in assessment are mutually exclusive). In each scenario, the patient pathway from the point when patients were invited for screening to receipt of a definitive diagnosis were identified and described. The screening procedure ends at the point of a patient receiving their iFOBT result with encouragement to attend hospital for a colonoscopy (if iFOBT is positive). It is important to note that the BIA included costs of screening and diagnosis (e.g., colonoscopy, biopsy) but not treatment. The BIA also did not address issues with respect to equity of access and uptake of services in either screening scenarios.

For peer review only - http://bmjopen.bmj.com/site/about/guidelines.xhtml

The patient pathways for the ‘usual care’ practice and the novel CRC screening programme are presented in Figure 1 and 2, respectively. In opportunistic screening practice, it is recommended or expected that individuals who are aged 50-75 years will be screened for CRC symptoms when they attend their local health clinic (for any health condition or problem). If they are asymptomatic and have an average risk of having CRC (based on family history), they are offered an iFOBT, followed by a colonoscopy if the iFOBT test was positive. If CRC is detected following a colonoscopy, the result is conveyed to a patient along with an explanation of the treatment plan or referral arrangement.

(Figure 1 is about here)

Details of the home-based screening intervention in CRC-SIM were published elsewhere [12]. Briefly, in the novel CRC screening programme, individuals aged 50-75 years were contacted, checked for eligibility, and invited to participate. A home-screening ‘pack’ was posted to eligible participants followed by two reminders. The test was performed at home by participants who took a photograph of the completed test and texted it to trained medical professionals who interpreted the photograph. Participants with positive iFOBT were referred for a colonoscopy at hospital.

(Figure 2 is about here)

There were two main differences between these patient pathways. Firstly, individuals within the target age group for screening were contacted directly and invited to participate in the novel CRC screening programme while in the situation of ‘usual care’, CRC screening was offered (if screening guideline recommendations were followed) only when members of the target group visited their clinic for some other health condition or problem. Secondly, the iFOBT was performed by doctors at health clinics in the ‘usual care’ pathway while in the novel CRC screening programme, participants self-tested in their home. Home-based testing generated additional stages in the pathways in relation to sending a test, reminding participants, taking a

For peer review only - http://bmjopen.bmj.com/site/about/guidelines.xhtml

photo of a completed test, and sending it to programme officers and vice versa. The remaining stages of each pathway (e.g., being screened for eligibility, receiving a colonoscopy, and receiving a treatment plan) were the same across the two scenarios.

Eligible population and input assumptions

The target population for current opportunistic screening in Malaysia is individuals aged 50-75 years, regardless of sex. Due to the nature of home-based screening, the target population for the CRC screening programme was required to meet some additional inclusion criteria as presented in Figure 2. The number of individuals who presented and completed each stage was estimated using input assumptions.

Data about the population of Malaysia by age was taken from government reports (i.e., Department of Statistics, Malaysia) and from World Population Review [13, 14]. The total population was reported to be 32,676,786 in 2021, of which, 19% or 6,228,195 were aged 50-75 years old [13, 14].

In the 'usual care' – opportunistic screening pathway or scenario, all assumptions were derived from a study by Tamin NSI (2020) which was a 5-year evaluation of opportunistic CRC screening (and the use of stool-based tests) in community health clinics across Malaysia [5]. It was assumed that 0.482% of the eligible population would avail of CRC screening when they attended local health clinics for other conditions; and 9.21% of this proportion of tested patients would receive a positive result. Only 55.9% of patients in the study by Tamin availed of a colonoscopy after a positive iFOBT. CRC detection after colonoscopy investigation was 4.04%.

For peer review only - http://bmjopen.bmj.com/site/about/guidelines.xhtml

In the novel CRC screening programme, all assumptions were derived from the CRC-SIM research project. It was assumed that 50.51% of the eligible population would be contactable and meet all inclusion criteria to participate in the home-based screening programme; 52.27% of people who were eligible would agree to participate; 41.63% would perform the iFOBT and send a photo of a completed test to the programme officers; 18.01% of people who would be tested would receive a positive result; 41.07% would avail of colonoscopy after a positive iFOBT result; and CRC detection after colonoscopy investigation would be 4.35%.

Table 1 summarises details about the input assumptions that were used to estimate the number of individuals at each stage of the respective pathway: the opportunistic screening pathway and the CRC screening programme pathway.

Table 1: Input assumptions used to estimate the population at each stage of the patient pathways

**Stage in pathway**	**Opportunistic screening scenario (Current practice)**		**Population-based CRC programme screening scenario (Proposed practice)**	
	Assumption*	No. of individuals	Assumption**	No. of individuals
Total population (all ages)	NA	32,676,786	NA	32,676,786
Target population (aged 50-75)	19.06%	6,228,195	19.06%	6,228,195
Eligible population (met all inclusion criteria)	100%	6,228,195	50.51%	3,146,020
Availed of/agreed to take CRC screening	0.482%	30,020	52.27%	1,644,561
Needed 1st reminder to return the iFOBT result (among those agreed to participate)	NA	NA	78.71%	1,294,514
Needed 2nd reminder to return the iFOBT result (among those received 1st reminder)	NA	NA	88.10%	1,140,405

For peer review only - http://bmjopen.bmj.com/site/about/guidelines.xhtml

Returned iFOBT result (among those agreed to participate)	100%	30,020	41.63%	684,683
Received iFOBT positive result	9.21%	2,765	18.01%	123,287
Availed of colonoscopy after positive iFOBT	55.9%	1,546	41.07%	50,636
CRC detection after colonoscopy investigation	4.04%	62	4.35%	2,202

CRC: Colorectal cancer; iFOBT: Immunochemical faecal occult blood test; NA: Not applicable; No: Number

** The assumptions were derived from a study of Tamin NSI (2020) which was a 5-year evaluation of using stool-based test for opportunistic CRC screening in primary health institutions across Malaysia [5].*

*** The assumptions were derived from the Colorectal Cancer Screening Intervention for Malaysia (or CRC-SIM research project) in Segamat District, conducted by Queen's University Belfast, Monash University, and Southeast Asia Community Observatory (SEACO) in 2021.*

For peer review only

Cost input and data sources

In the opportunistic screening scenario, the total cost comprised the cost of:

(i) performing screening (e.g., asking for symptoms, family history, and collecting the sample)

(ii) processing stool specimens

(iii) interpreting test results and

(iv) conveying a definitive diagnosis to patients (include explaining treatment plan or referral arrangements)

In the CRC programme screening scenario, the total cost comprised the costs of:

(i) contacting potential participants

(ii) delivering iFOBT test kits (including cost of the test, postage, print materials, and sending video instruction)

(iii) sending a reminder to participants (up to 2 times, by text message and phone call)

(iv) interpreting and conveying results to participants and

(v) following-up patients with positive iFOBT but did not take colonoscopy in order to encourage them to avail of the colonoscopy

For peer review only - http://bmjopen.bmj.com/site/about/guidelines.xhtml

These costs were calculated by multiplying the time allocated for the completion of each task with the salary cost of the person who undertakes each task plus cost of consumables. Table 2 shows the unit cost for each cost element, related assumptions, and data sources.

Table 2: Resources and unit costs

Currency: Malaysian ringgit (RM)

Cost element	Unit cost *(Per screen) RM (I$)*	Assumptions	Source
Current practice (opportunistic screening)			
Performing screening (asking for symptoms, family history, referral) and taking sample	5.58	20 min x salary RM2947/month	1
Processing stool specimens	1.70	10 min x salary RM1797/month	2
Interpreting the test results	2.79	10 min x salary RM2947/month	1
Conveying a definitive diagnosis to patients (along with explaining treatment plan or referral etc.)	8.37	30 min x salary RM2947/month	1
Proposed practice (Population-based CRC screening programme)			
Contact eligible individuals - agreed to participate	0.98	7.1 min x (salary RM1440/month + mobile package RM20/month)	3
Contact eligible individuals - rejected/excluded to participate	0.47	3.4 min x (same as above)	3
iFOBT rapid test kit	6.90		3
Print materials (instruction leaflet, explanatory statement)	1.10	90 cents for colour print + 20 cent for black & white print	3
Postage (stamps, etc.)	5.35		3
Sending video through WhatsApp	0.41	3 min x (salary RM1440/month + mobile package RM20/month)	3
Sending reminder text message	0.41	3 min x (same as above)	3
Reminder call	0.28	2 min x (same as above)	3
Interpreting the test kit result	1.70	10 min x salary RM1797/month	3
Sending text message to inform patient of negative result	0.45	2 min x (salary RM2350/month + mobile package RM20/month)	3
Calling patient to inform him/her of positive result	0.67	3 min x (same as above)	3

For peer review only - http://bmjopen.bmj.com/site/about/guidelines.xhtml

Currency: Malaysian ringgit (RM)

Cost element	Unit cost *(Per screen) RM (I$)*	Assumptions	Source
Preparing and sending referral letter to patient/clinic	1.12	5 min x (same as above)	3
Follow up effort	6.73	30 min x (same as above)	3
Developing communication materials, **one-off cost**	6,063	Communication materials do not change in 5 years	3
Training for data collectors*, **one-off cost**<br>** Data collectors are those employed by the programme to (i) contact potential participants, (ii) deliver iFOBT test kits, and (iii) send a reminder to participants*	109,703	+ 1 day training (virtual using Zoom)<br>+ 1 trainer for maximum 25 trainees<br>+ 1 data collector* is needed for every target population of 400<br>+ Cost=1-day-salary of trainer/trainees x number of trainer/trainees<br>+ No retraining in 5 years	3
Same in both scenarios/practices			
Colonoscopy (including polyps removal and/or biopsy if needed)	200		
Consumables – stool container, gloves, mask, plastic waste bag and disposal of materials from the test	10.80	RM8636.7/800 sets	3

iFOBT: Immunochemical faecal occult blood test; RM: Malaysian ringgit

Source:

1. *Public Services Commission of Malaysia. Medical Officer Grade UD41. Accessed at https://www.spa.gov.my/spa/laman-utama/gaji-syarat-lantikan-deskripsi-tugas/ijazah-sarjana-phd/pegawai-perubatan-gred-ud41*
2. *Public Services Commission of Malaysia. Medical laboratory technologist Grade U29. Accessed at https://www.interactive.jpa.gov.my/ezskim/klasifikasi/perbekalanskim.asp?id_skim=3LU03*
3. *Colorectal Cancer Screening Intervention for Malaysia (or CRC-SIM research project) in Segamat District, conducted by Queen's University Belfast, Monash University, and Southeast Asia Community Observatory (SEACO) in 2021*

In the current practice of opportunistic screening, doctors were consulted about the estimated time to perform each stage in the pathway. The monthly salary of a general doctor and a medical laboratory technologist was based on the rate published by the Public Services Commission of Malaysia [15, 16]. These rates were RM2,947 (~I$2,045) and RM1,797 (~I$1,247), respectively. In the novel CRC screening programme, the time to perform each stage in the pathway, salary of personnel, and costs of material resources (e.g., rapid kit test, consumables, postage, printing materials) were based on the time and expenditure observed in the CRC-SIM research project.

For peer review only - http://bmjopen.bmj.com/site/about/guidelines.xhtml

All costs were calculated per screen except the cost of training and the cost of developing communication materials which were one-off costs based on the assumption that communication materials would not change, and no re-training would be needed within 5 years. It was assumed (based on the experience of operating the screening programme during the CRC-SIM project) that one data collector (i.e., those employed by the programme to (i) contact potential participants, (ii) deliver iFOBT test kits, and (iii) send a reminder to participants) would be needed for every 400 people in the target population. Training would last one day and would be delivered virtually; thus, the cost of training equalled (1-day-salary of trainer x number of trainer) + (1-day-salary of trainees x number of trainees/data collectors).

Perspective and time horizon

The BIA was conducted from the viewpoint of the federal government which finances Malaysia's public health system [17]. Only those costs and resource requirements relevant to the budget holder were included in the analysis. For example, the out-of-pocket expenditure incurred by patients were excluded.

The analysis estimated the annual financial impact over a period of five years as recommended in the guidelines [10, 18]. Costs were not discounted given that the BIA methodology reports the costs for each year in which they occur rather than a net present value [10].

For peer review only

Budget impact analyses

Computing framework and base-case analysis

The BIA used a cost calculator programmed in Microsoft Excel, following the costing template[1] produced by the National Institute for Health and Care Excellence in the UK (NICE). The template was modified to fit the programme under assessment. The cost calculator approach is recommended by guidelines as it is easy for stakeholders to understand and replicate the results [10].

First, the number of individuals who completed each stage was estimated (Table 1). The resources that were used at each stage of the respective pathways (in opportunistic screening and the novel CRC screening programme) were listed along with their unit costs (i.e., cost of each resource per person) (Table 2). Unit costs were multiplied by number of users to give the total cost of resources for each scenario. The net budget impact was calculated as the difference in cost between opportunistic screening and the CRC screening programme. Visual depiction of the cost calculator is shown in Supplementary Material, Figure S1.

Uncertainty and scenario analyses

The input assumptions (that were used to estimate the number of individuals at each stage of the respective pathway) and the cost inputs were varied, and then the impact of these changes in relation to the results was analysed to investigate the sensitivity of the budget impact results to variations in individual input. As recommended by Gray et al. (2011), the range of variation regarding parameters for which data sources about dispersion were unavailable were ±20% of the base case [19].

---

[1] The template can be freely downloaded at https://www.nice.org.uk/Media/Default/About/what-we-do/our-programmes/evidence-standards-framework/budget-impact-template.xlsx

For peer review only - http://bmjopen.bmj.com/site/about/guidelines.xhtml

Patient and public involvement

It was not appropriate or possible to involve patients or the public in the design, or conduct, or reporting, or dissemination plans of our research as this type of study is a secondary analysis of data from a payer perspective (Ministry of Health Malaysia).

RESULTS

Base-case analysis

The total annual cost of the current practice of opportunistic screening is RM1,584,321 (~I$1,099,460), of which 80% (RM1,274,690 ~ I$884,587) was for providing opportunistic CRC to adults who availed of the service. Costs of providing colonoscopy (including polyps removal and/or biopsy if needed) after receipt of a positive iFOBT and conveying definitive diagnosis to patients (along with explaining treatment plan or referral etc.) after the outcome of the colonoscopy were RM309,108 (~I$214,509) and RM523 (~I$363), respectively.

The total annual cost over a 5-year period of the proposed practice (i.e., CRC screening programme) is shown in Table 3. It was assumed that the number of people who would agree to participate in the programme would increase by 5% each year (in consideration of health promotion activities as well as information flows including word of mouth between participants). Therefore, the financial impact would also increase accordingly.

For peer review only - http://bmjopen.bmj.com/site/about/guidelines.xhtml

Table 3: Annual cost of proposed practice (i.e., CRC screening programme)

Currency: Malaysian ringgit (RM) and International Dollar (I$)

Proposed practice	Year 1 RM *(I$)*	Year 2 RM *(I$)*	Year 3 RM *(I$)*	Year 4 RM *(I$)*	Year 5 RM *(I$)*
Contacting adults who are eligible for CRC screening programme (i.e., aged 50-75) and screen for eligibility of participating	2,320,148 *(1,610,096)*	2,320,148 *(1,610,096)*	2,320,148 *(1,610,096)*	2,320,148 *(1,610,096)*	2,320,148 *(1,610,096)*
Providing iFOBT test to adults who agreed to participate in CRC screening programme after being invited	93,654,886 *(64,992,981)*	102,612,907 *(71,209,512)*	111,570,928 *(77,426,043)*	120,528,949 *(83,642,574)*	129,486,970 *(89,859,105)*
Providing 1st reminder to participants	536,929 *(372,609)*	588,286 *(408,248)*	639,643 *(443,888)*	690,999 *(479,527)*	742,356 *(515,167)*
Providing 2nd reminder to participants	315,339 *(218,833)*	345,501 *(239,765)*	375,663 *(260,696)*	405,825 *(281,627)*	435,987 *(302,559)*
Interpreting returned iFOBT samples	1,165,129 *(808,556)*	1,276,572 *(885,893)*	1,388,016 *(963,231)*	1,499,460 *(1,040,569)*	1,610,903 *(1,117,906)*
Conveying result through message to participants with iFOBT negative result	251,990 *(174,872)*	276,093 *(191,598)*	300,196 *(208,324)*	324,298 *(225,050)*	348,401 *(241,777)*
Preparing and sending referral letter and calling participants with iFOBT POSITIVE result	221,356 *(153,613)*	242,529 *(168,306)*	263,701 *(182,999)*	284,874 *(197,692)*	306,046 *(212,384)*
Following up participants who DID NOT take colonoscopy after positive iFOBT	489,158 *(339,457)*	535,945 *(371,926)*	582,733 *(404,394)*	629,520 *(436,863)*	676,308 *(469,332)*
Providing colonoscopy (including polyps removal and/or biopsy if needed) to participants with positive iFOBT	10,127,147 *(7,027,861)*	11,095,801 *(7,700,070)*	12,064,455 *(8,372,280)*	13,033,109 *(9,044,489)*	14,001,764 *(9,716,700)*
Conveying definitive diagnosis to patients (along with explaining treatment plan or referral etc.) after the colonoscopy	18,432 *(12,791)*	20,195 *(14,015)*	21,958 *(15,238)*	23,721 *(16,461)*	25,484 *(17,685)*
Capital costs (Developing communication materials + Training for data collectors)	115,766 *(80,337)*	115,766 *(80,337)*	115,766 *(80,337)*	115,766 *(80,337)*	115,766 *(80,337)*
**Total cost of proposed practice**	**109,216,279** *(75,792,005)*	**119,429,743** *(82,879,766)*	**129,643,206** *(89,967,527)*	**139,856,670** *(97,055,288)*	**150,070,133** *(104,143,049)*

CRC: Colorectal cancer; iFOBT: Immunochemical faecal occult blood test

For peer review only - http://bmjopen.bmj.com/site/about/guidelines.xhtml

Similar to opportunistic screening, the cost to provide iFOBT to the eligible population who availed of the service accounted for 86% of the total cost of the proposed CRC screening programme. The second most costly component was the provision of colonoscopy (including polyps removal and/or biopsy if needed) to patients with an iFOBT positive result, at 9% of the total cost. The remaining nine cost components such as contacting potential participants, reminding participants to send photograph of iFOBT result, conveying diagnosis to participants and the follow-up effort added only up to 5% of the total cost.

The net budget impact in the 1st year of implementing CRC screening programme would be RM107,631,959 (~I$74,692,546 which equalled the total cost of future practice minus the total cost of current practice). The impact increases each year as the number of people who agree to participate in the programme increase, reaching RM117,845,422 (~I$81,780,307) in year 2, RM128,058,885 (~I$88,868,067) in year 3, RM138,272,349 (~I$ 95,955,829) in year 4, and RM148,485,812 (~I$103,043,589) in year 5.

The net budget impact of providing and delivering the CRC screening programme over the 5-year timeframe for each state in Malaysia (calculated according to the population size of each state) can be accessed in Supplementary Material, Table S1. These estimates aid service planning decisions if the novel pilot programme is implemented in one or more of these states before being scaled up into nationwide programme.

Uncertainty and scenario analyses

The tornado diagram in Figure 3 shows the change to net budget impact when assumptions and cost inputs were varied. It presents the results of multiple univariate sensitive analyses on key inputs that exert the most influence on the net budget impact (See Table S2, Supplementary

For peer review only - http://bmjopen.bmj.com/site/about/guidelines.xhtml

For peer review only

Material for results of multiple univariate sensitive analysis on all inputs). These inputs include the probability of (i) making successful contact with adults about the CRC screening programme, (ii) adults agreeing to participate, (iii) adults being eligible to participate in the programme, and (iv) the cost of consumables that are required to take a stool sample. The first three inputs influence the number of individuals who are present at each stage of the patient pathway.

(Figure 3 is about here)

The net budget impact would increase from RM107 million to RM130 million (~I$74-90 million) if there was a 20% increase in (i) the probability of adults who were contactable (from a contact list of people aged 50-75 years old) or (ii) the probability of adults agreeing to participate in the CRC screening programme or (iii) the probability of adults being eligible for the programme (i.e., aged 50-75 years old; having no symptoms of CRC, a smartphone, and WhatsApp; resident within programme area; and did not have colonoscopy this year). In other words, a 20% increase in each one of these factors would require an additional RM23 million (~I$16 million) to be budgeted for the programme. Likewise, a 20% increase in the cost of the consumables that are required for taking stool samples would mean that the programme would cost RM15 million (~I$10 million) more than the originally calculated total cost.

DICUSSION

The result of this analysis provides information to guide public health service planners and commissioners in their decisions about an alternative CRC screening strategy i.e., a population-based CRC screening programme using home-based iFOBT compared to current opportunistic screening. It concluded that the net budget impact in the 1st year of implementing a CRC screening programme of this kind would be RM107,631,959 (~I$74,692,546). The impact would increase by year due to increase in uptake and would reach RM148,485,812 (~I$103,043,589) in the 5th year of implementation. This analytical approach and the results of

For peer review only - http://bmjopen.bmj.com/site/about/guidelines.xhtml

this analysis are presented as aids to better decision making by MoHs and stakeholders in lower-middle-income countries (LMICs) about health programme planning and in this particular illustrative case to the MoHM regarding the degree to which the proposed CRC screening programme is affordable.

The total budget that was allocated to the MoHM in 2022 was RM32.4 billion (~I$22.5 million) [20]. Spending on prevention and public health services in 2009 was reported to be RM1.6 billion (~I$1.1 million) [17]. More recent data and information about the size of the budget that is allocated to cancer screening is not available. As such, it is estimated that the net budget impact of implementing a CRC screening programme would account for between 7-10% of the total budget for prevention and public health services. This represents a significant proportion of the overall budget allocated for prevention programmes/interventions.

The key factor in the implementation of a population-based screening programme/service or the factor that has biggest impact on the budget is the size of the population who use the service. The degree of accuracy regarding population size estimates is related closely to the cost estimates in the budget. It is important for service planners to keep this point in mind and to take into account an increase in uptake and the impact of such an increase. Therefore, in the case of the CRC programme presented here, we assumed a 5% increase annually in uptake and calculated the net budget impact. The net budget impact can be recalculated according to the actual change in uptake after the programme is implemented.

Budget impact analysis is an economic assessment that is used to estimate the changes in expenditure of a specific budget holder if a new health technology/programme is implemented [10]. As such, BIA complements other health economic evaluation methods such as cost-

For peer review only - http://bmjopen.bmj.com/site/about/guidelines.xhtml

effectiveness analysis (CEA) to provide a comprehensive economic assessment of a health care intervention to decision makers [10]. A BIA aids decision making by health service planners and commissioners about whether an intervention or programme is affordable within given budget constraints while a CEA informs decisions about whether an intervention is good value for money [10, 21]. BIA and its pragmatic approach is an ideal method when a situation calls for an evaluation of 'affordability' which is of central importance in LMICs and, arguably, is the key concern of whoever is in charge of managing a health care budget [22, 23].

It could well be that savings in earlier treatment would counterbalance the additional budget impact. However, a BIA is not intended to provide answers to questions about whether or not, in this context, the screening programme is good value for money as it does not take into account the potential improvements in outcomes (e.g., increase quality-related life years) and savings from lower treatment costs for CRC diagnosed at earlier stages. The conduct of other types of economic evaluations such as a cost-effectiveness analysis would be required to provide a complete and comprehensive set of evidence for decision makers.

Finally, the conduct of BIA in this paper has some limitations. First, assumptions and cost inputs for the CRC screening programme were based on the costs and rates that were observed in the CRC-SIM research project. The project was conducted in only one district (Segamat); and the distribution of three main ethnic groups (i.e., Malay, Chinese, Indian) in the project differed from the proportions that have been reported nation-wide (72%:24%:3% vs 62%:21%:6%, respectively). Therefore, it is important to be mindful of the possibility that the assumptions and inputs (based on the project) may not be representative for, or read across to, the whole population of Malaysia. Likewise, it is important to bear in mind that our findings do not include the perspective of other payers and may not generalise to other settings. The

For peer review only - http://bmjopen.bmj.com/site/about/guidelines.xhtml

For peer review only

results are related directly to the context of the Malaysian health system and the epidemiology of CRC in the country though they are illustrative of the positive contribution of the BIA methodology and approach.

CONCLUSIONS

This study employed a BIA methodology to analyse the costs of a novel CRC screening programme using home-based iFOBT and mHealth versus the current opportunistic screening. The findings estimated the net budget impact of implementing a population-based national CRC screening programme in Malaysia. The modelling estimations are important considerations for health authorities when they are required to decide the affordability of implementing a programme and to aid budgetary planning as well as decision making, generally, about implementation. Our study illustrates the use and value of the BIA approach in LMICs and resource-constrained settings.

For peer review only

Abbreviations

BIA	Budget impact analysis
CEA	Cost-effectiveness analysis
CRC	Colorectal cancer
CRC-SIM	Colorectal Cancer Screening Intervention for Malaysia
iFOBT	Immunochemical faecal occult blood test
I$	International Dollar
ISPOR	The Professional Society for Health Economics and Outcomes Research
MoHM	Ministry of Health of Malaysia
NICE	National Institute for Health and Care Excellence
RM	Malaysian ringgit

For peer review only - http://bmjopen.bmj.com/site/about/guidelines.xhtml

SEACO	Southeast Asia Community Observatory
UK	The United Kingdom

DECLARATIONS

Research ethics approval. Not applicable (This study does not involve human participants).

Funding. This research was supported by the Medical Research Council (UK) Global Challenges Research Fund (MR/S014349/1) and the National Medical Research Register Malaysia (NMRR ID-21-02045-O7G). The funder of the study had no role in study design, data collection, data analysis, data interpretation, or writing of the report.

Competing interests. The authors have no conflicts of interest to declare that are relevant to the content of this article.

Availability of data and material. All data generated or analysed during this study are included in this published article.

Authors' contributions. TTN: Methodology, Formal Analysis, Writing – Original draft, Writing – Review & Editing. KR, MRS, and DS: Data Curation, Investigation, Writing – Review & Editing. ST: Resources, Writing – Review & Editing. TTS, and MD: Conceptualization, Funding acquisition, Writing – Review & Editing, Supervision. CON: Methodology, Formal Analysis, Writing – Review & Editing, Supervision.

For peer review only

REFERENCE

1. Global Cancer Observatory: Cancer Today [Internet]. International Agency for Research on Cancer. 2020 [cited 12 April 2021]. Available from: http://gco.iarc.fr/today.
2. Malaysian National Cancer Registry Report 2012-2016 [Internet]. Ministry of Health Malaysia. 2020 [cited April 12, 2022].
3. Veettil SK, Lim KG, Chaiyakunapruk N, Ching SM, Abu Hassan MR. Colorectal cancer in Malaysia: Its burden and implications for a multiethnic country. Asian Journal of Surgery. 2017;40(6):481-9.
4. Rawla P, Sunkara T, Barsouk A. Epidemiology of colorectal cancer: incidence, mortality, survival, and risk factors. Prz Gastroenterol. 2019;14(2):89-103.
5. Tamin NSI, Razalli KA, Sallahuddin SN, Chan HK, Hassan MRA. A 5-year evaluation of using stool-based test for opportunistic colorectal cancer screening in primary health institutions across Malaysia. Cancer epidemiology. 2020;69:101829.

6. Ministry of Health Malaysia. Clinical Practice Guidelines – Management of Colorectal Carcinoma. Putrajaya: Malaysia Health Technology Assessment Section (MaHTAS); 2017.

7. Schliemann D, Ramanathan K, Ibrahim Tamin NSB, Neill C, Cardwell CR, Ismail R, et al. Implementation of a colorectal cancer screening intervention in Malaysia (CRC-SIM) in the context of a pandemic: study protocol. BMJ Open. 2022;12(9):e058420.

8. Issa IA, Noureddine M. Colorectal cancer screening: An updated review of the available options. World journal of gastroenterology. 2017;23(28):5086-96.

9. Mauskopf JA, Sullivan SD, Annemans L, Caro J, Mullins CD, Nuijten M, et al. Principles of good practice for budget impact analysis: report of the ISPOR Task Force on good research practices--budget impact analysis. Value Health. 2007;10(5):336-47.

10. Sullivan SD, Mauskopf JA, Augustovski F, Jaime Caro J, Lee KM, Minchin M, et al. Budget impact analysis-principles of good practice: report of the ISPOR 2012 Budget Impact Analysis Good Practice II Task Force. Value Health. 2014;17(1):5-14.

11. World Economic Outlook Databases [Internet]. 2022 [cited April 13]. Available from: https://www.imf.org/en/Publications/SPROLLS/world-economic-outlook-databases#sort=%40imfdate%20descending.

12. Budget impact analysis of a population-based screening programme for colorectal cancer in Malaysia: technical report of a modelling study [Internet]. Centre for Public Health, Queen's University Belfast. 2022 [cited July 1]. Available from: https://pure.qub.ac.uk/en/publications/budget-impact-analysis-of-a-population-based-screening-programme-.

13. Malaysia Population Pyramid 2022 [Internet]. World Population Review. [cited Jan 21, 2022]. Available from: https://worldpopulationreview.com/countries/malaysia-population.

14. Demographic Statistics Factsheet [Internet]. Department Statistics of Malaysia. 2021 [cited Jan 21, 2022]. Available from: https://www.dosm.gov.my/v1/index.php?r=column/cthemeByCat&cat=430&bul_id=N05ydDRXR1BJWVlTdDY4TldHd253dz09&menu_id=L0pheU43NWJwRWVSZklWdzQ4TlhUUT09.

15. Medical Officer Grade UD41 [Internet]. Public Services Commission of Malaysia. [cited January 27, 2022]. Available from: https://www.spa.gov.my/spa/laman-utama/gaji-syarat-lantikan-deskripsi-tugas/ijazah-sarjana-phd/pegawai-perubatan-gred-ud41.

16. Medical laboratory technologist Grade U29 [Internet]. Public Services Commission of Malaysia. [cited January 27, 2022]. Available from: https://www.interactive.jpa.gov.my/ezskim/klasifikasi/perbekalanskim.asp?id_skim=3LU03.

17. Jaafar S, Noh KM, Muttalib KA, Othman NH, Healy J. Malaysia Health System Review. Asia Pacific Observatory on Health Systems and Policies; 2013.

18. Health Information and Quality Authority. Guidelines for the budget impact analysis of health technologies in Ireland. 2018.

19. M.Gray A, Clarke PM, Wolstenholme JL, Wordsworth S. Applied Methods of Cost-effectiveness Analysis in Health Care. Gray AM, Briggs A, editors. New York: Oxford University Press; 2011.

20. Ministry of Finance Malaysia. Budget 2022: RM32.4 billion allocation for MOH. Official Portal of Ministry of Finance Malaysia. 2021 [cited 2022 April 13]. Available from: https://www.mof.gov.my/portal/en/news/press-citations/budget-2022-rm32-4-billion-allocation-for-moh.

21. Leelahavarong P. Budget Impact Analysis. J Med Assoc Thai. 2014;97(Suppl. 5):S65-S71.

22. Orlewska E, Gulácsi L. Budget-Impact Analyses: A Critical Review of Published Studies. PharmacoEconomics. 2009;27(10):807-27.

23. Garattini L, van de Vooren K. Budget impact analysis in economic evaluation: a proposal for a clearer definition. The European Journal of Health Economics. 2011;12(6):499.

For peer review only - http://bmjopen.bmj.com/site/about/guidelines.xhtml

Figure Legends and Tables

Table 1 Input assumptions used to estimate the population at each stage of the patient pathways

Table 2 Resources and unit costs

Table 3 Annual cost of proposed practice (i.e., CRC screening programme)

Figure 1 Patient pathways in ‘usual care’ practice - opportunistic screening for CRC

Figure 2 Patient pathway in population-based CRC screening programme

Figure 3 Results of multiple univariate sensitive analyses showing key factors that exert most influence the net budget impact

Supplementary material

Figure S1 Visual depiction of the budget impact cost calculator

Table S1 Net budget impact of CRC screening programme over 5-year timeframe, by state

Table S2 Sensitivity of the total budget impact of CRC screening programme to changes in each variable individually

For peer review only

For peer review only - http://bmjopen.bmj.com/site/about/guidelines.xhtml

Figure 1: Patient pathway in 'usual care' practice - opportunistic screening for CRC

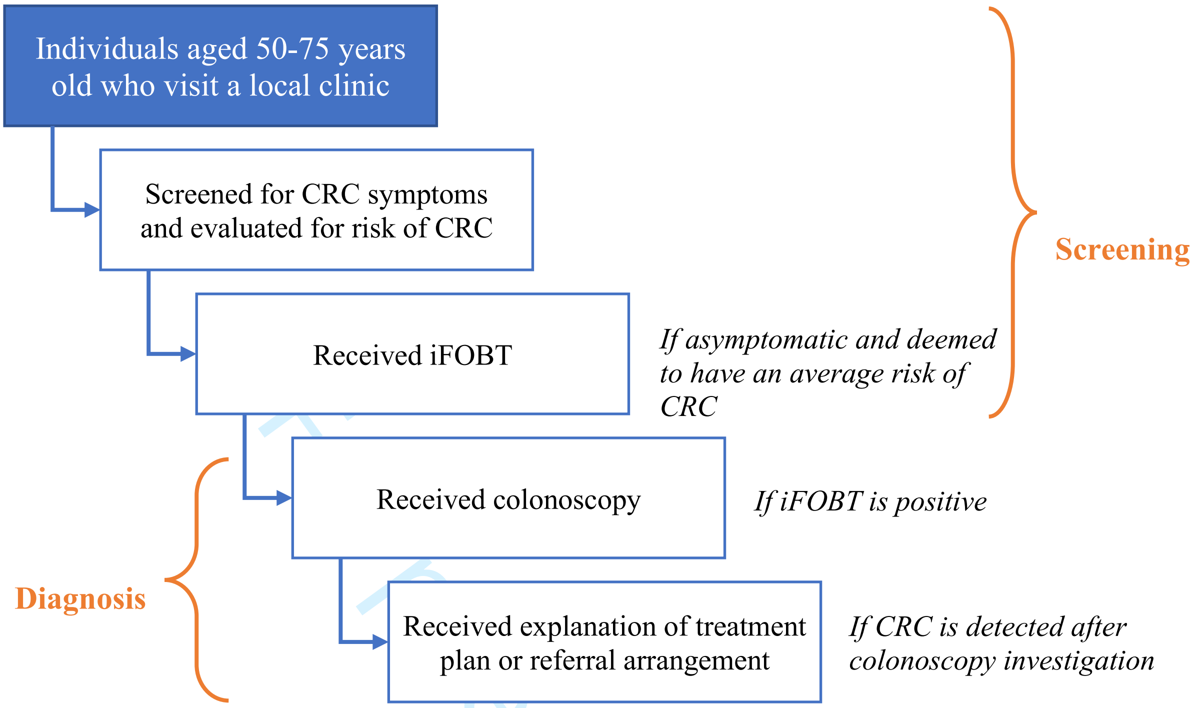

Figure 2: Patient pathway in population-based CRC screening programme

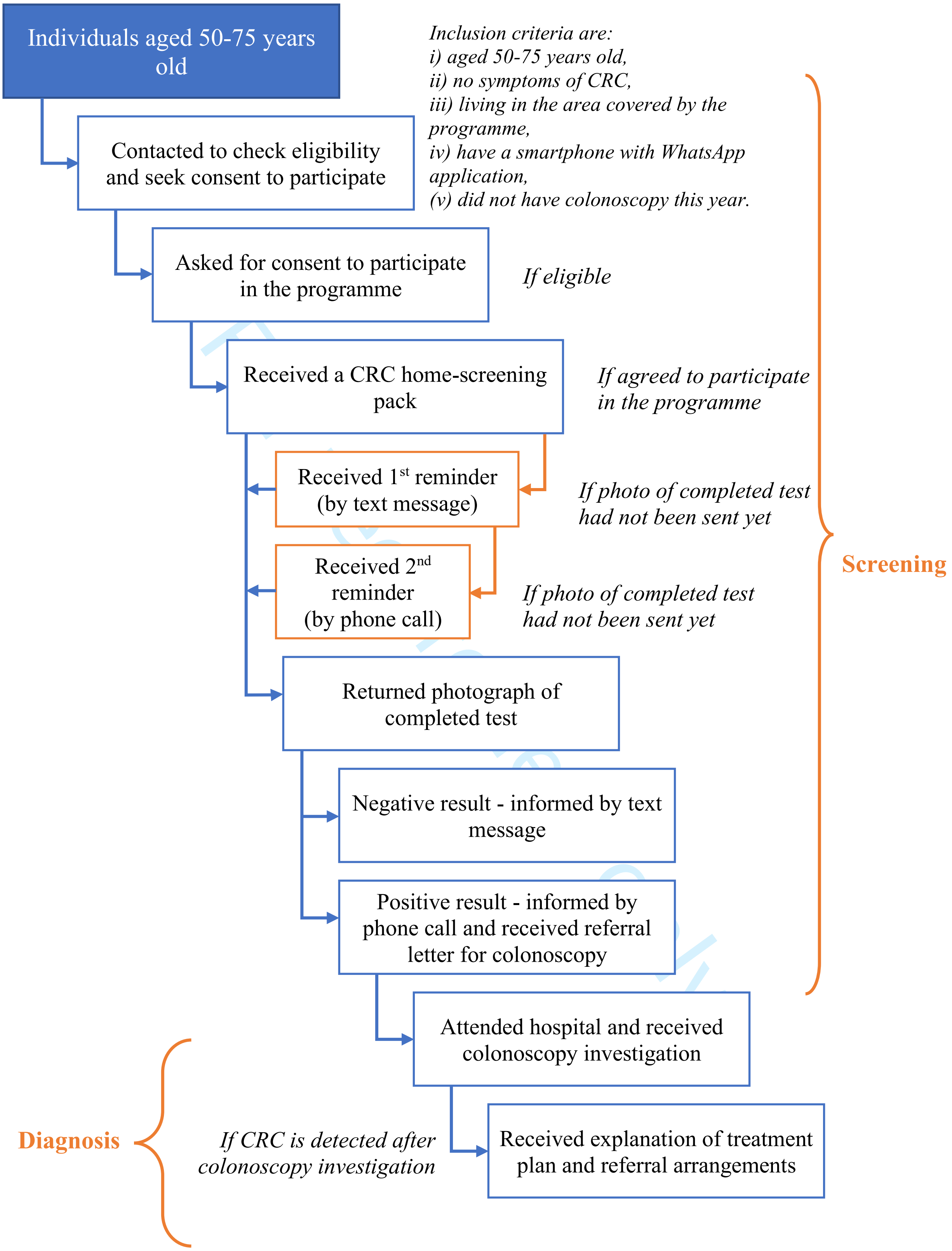

Figure 3: Results of multiple univariate sensitive analyses showing key factors that exert most influence the net budget impact

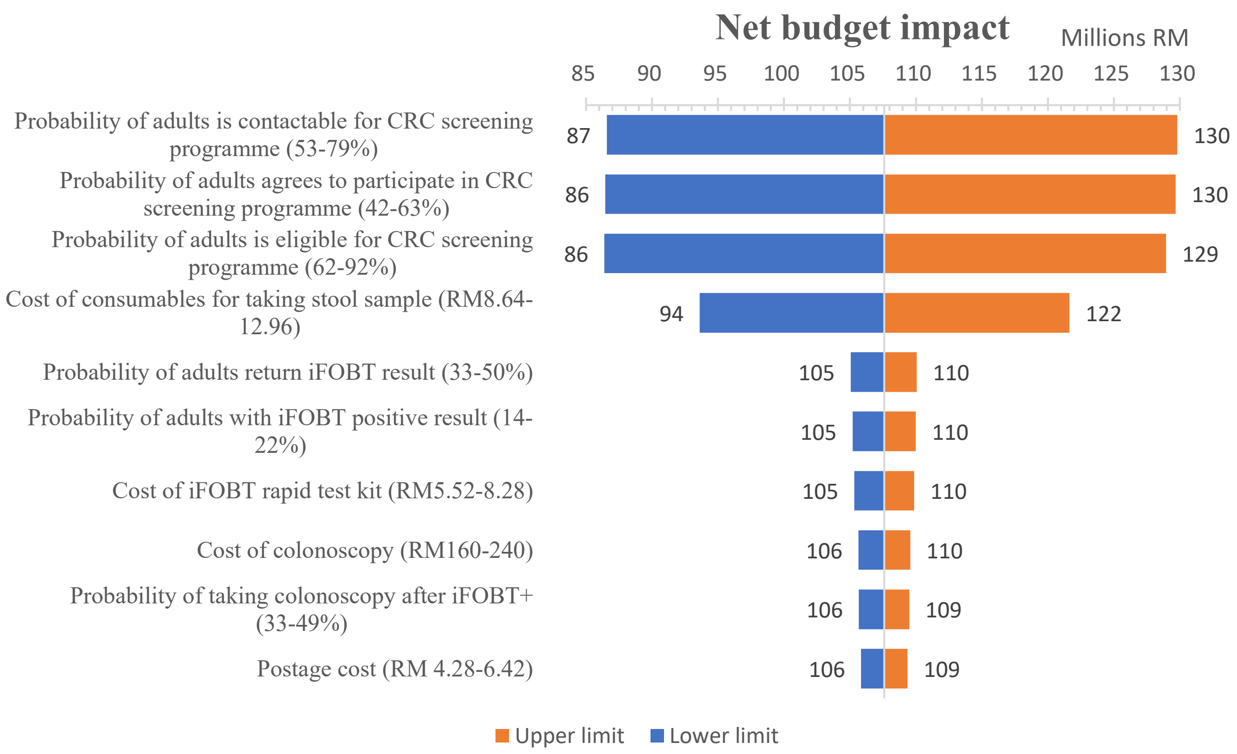

Supplementary material

Figure S1: Visual depiction of the budget impact cost calculator

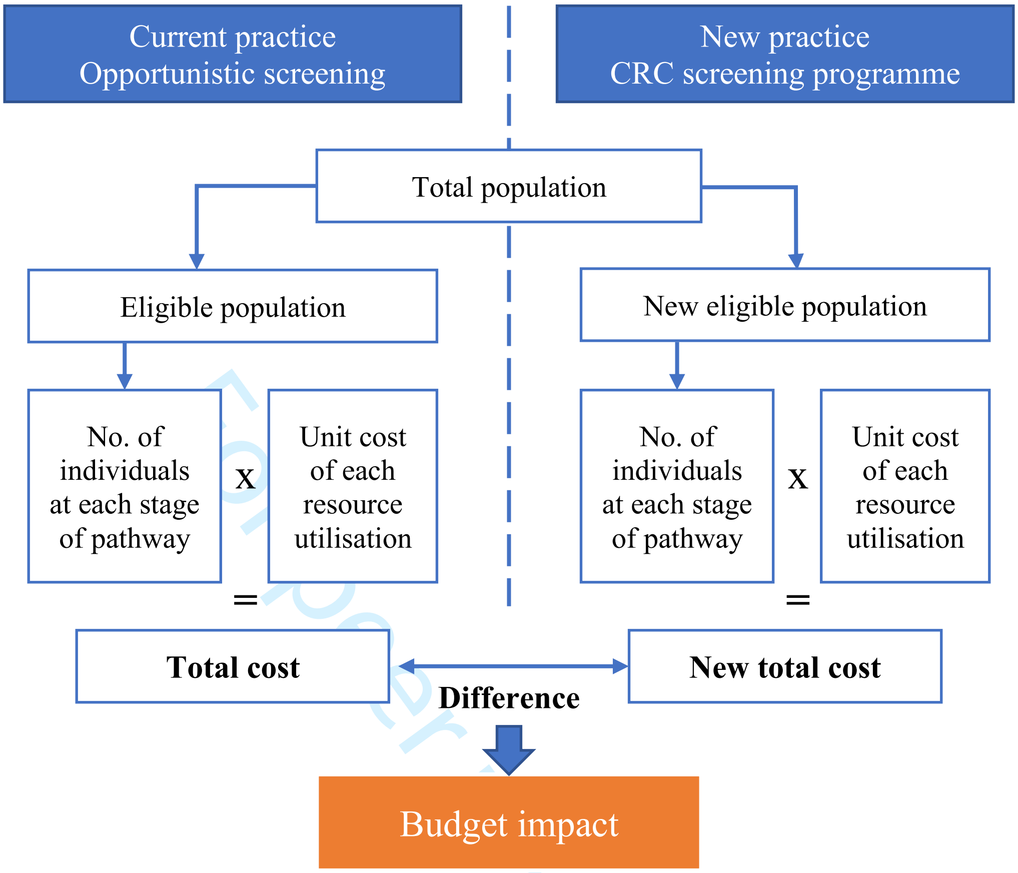

Supplementary material

Table S1: Net budget impact of CRC screening programme over 5-year timeframe, by state

Currency: Malaysian ringgit

State	Population	Year 1	Year 2	Year 3	Year 4	Year 5
**Johor**	3,822,800	12,597,041	13,791,897	14,986,752	16,181,607	17,376,462
**Kedah**	2,206,200	7,272,541	7,962,112	8,651,682	9,341,252	10,030,823
**Kelantan**	1,884,300	6,212,311	6,801,268	7,390,225	7,979,183	8,568,140
**Melaka**	934,700	3,084,637	3,376,787	3,668,937	3,961,087	4,253,238
**Negeri Sembilan**	1,141,000	3,764,122	4,120,753	4,477,384	4,834,016	5,190,647
**Pahang**	1,702,900	5,614,833	6,147,092	6,679,351	7,211,609	7,743,868
**Perak**	2,569,300	8,468,450	9,271,511	10,074,572	10,877,633	11,680,693
**Pulau Pinang**	1,767,100	5,826,301	6,378,626	6,930,951	7,483,276	8,035,601
**Sabah**	3,919,600	12,915,864	14,140,975	15,366,086	16,591,197	17,816,308
**Sarawak**	2,829,400	9,325,144	10,209,501	11,093,859	11,978,217	12,862,575
**Terengganu**	1,245,300	4,107,648	4,496,879	4,886,111	5,275,342	5,664,573
**Perlis**	253,500	841,028	920,262	999,496	1,078,730	1,157,964
**W.P. Kuala Lumpur**	1,790,100	5,902,043	6,461,557	7,021,071	7,580,585	8,140,099
**W.P. Labuan**	99,000	332,138	363,081	394,024	424,968	455,911
**W.P. Putrajaya**	97,100	325,886	356,236	386,585	416,935	447,284
**Nation-wide**	**32,676,786**	**107,631,959**	**117,845,422**	**128,058,885**	**138,272,349**	**148,485,812**

CRC: Colorectal cancer | W.P.: The Federal Territories (Malay: Wilayah Persekutuan)

Readers can convert from Malaysian Ringgit to their currency of interest (e.g., International Dollar, US Dollar, British Pound, Euro etc.) using the free web-based tool 'CCEMG – EPPI-Centre Cost Converter' (https://eppi.ioe.ac.uk/costconversion/default.aspx). This tool help adjusting estimates of cost expressed in one currency and price year to a specific target currency and price year.

For peer review only

Supplementary material

Table S2: Sensitivity of the total budget impact of CRC screening programme to changes in each variable individually

Baseline budget impact=RM107,631,959

Unit: Thousand Ringgit Malaysia

	Baseline value	Min value (-20% from baseline)	Max value (+20% from baseline)	Min budget impact	Max budget impact	Change
Probability of adults is contactable for CRC screening programme	66%	53%	79%	86,574	129,818	43,244
Probability of adults is included (eligible for CRC screening programme)	77%	62%	92%	86,386	128,950	42,564
Probability of adults agree to participate in CRC screening programme after being invited	52%	42%	63%	86,454	129,675	43,221
Probability of adults needing 1st reminder	79%	63%	94%	107,430	107,766	336
Probability of adults needing 2nd reminder	88%	70%	100%	107,535	107,643	108
Probability of adults return iFOBT result	42%	33%	50%	105,062	110,060	4,998
Probability of adults with iFOBT positive result	18%	14%	22%	105,204	109,988	4,784
Probability of adults taking colonoscopy after positive iFOBT	41%	33%	49%	105,673	109,493	3,820
Probability of adults with CRC detection after getting colonoscopy	4%	3%	5%	107,594	107,603	9
Cost to perform the screening (asking for symptoms, family history, referral)	5.58	4.47	6.70	107,633	107,567	-66
Cost of stool specimen processing	1.70	1.36	2.04	107,610	107,590	-20
Interpretation of results	2.79	2.23	3.35	107,617	107,583	-34
Cost to convey definitive diagnosis to patients (along with explaining treatment plan or referral etc.)	8.37	6.70	10.05	107,597	107,604	7
Contact eligible individuals - agreed to participate	0.98	0.79	1.18	107,285	107,926	641
Contact eligible individuals - rejected/excluded to participate	0.47	0.38	0.56	107,465	107,735	270
iFOBT rapid test kit (only the rapid test kit itself)	6.90	5.52	8.28	105,331	109,870	4,539
Print materials (instruction leaflet, explanatory statement)	1.10	0.88	1.32	107,238	107,962	724
Postage (stamp etc.)	5.35	4.28	6.42	105,840	109,360	3,520
Sending video through Whatapp	0.41	0.33	0.50	107,461	107,740	279
Sending reminder text message	0.41	0.33	0.50	107,490	107,710	220
Reminder call	0.28	0.22	0.33	107,536	107,661	125
Interpret the test kit result	1.70	1.36	2.04	107,366	107,832	466

For peer review only

Supplementary material

Unit: Thousand Ringgit Malaysia

	**Baseline value**	**Min value (-20% from baseline)**	**Max value (+20% from baseline)**	**Min budget impact**	**Max budget impact**	**Change**
Sending text message informing negative result	0.45	0.36	0.54	107,550	107,651	101
Call to inform positive result	0.41	0.33	0.50	107,590	107,611	21
Prepare and send referral letter	1.12	0.90	1.35	107,573	107,628	55
Follow up effort	6.73	5.39	8.08	107,503	107,698	195
Colonoscopy	200	160	240	105,638	109,564	3,926
Consumables – stool container, gloves, mask, plastic waste bag and disposal of materials from the test	10.80	8.64	12.96	93,612	121,641	28,029

For peer review only

ISPOR—The Professional Society for Health Economics and Outcomes Research

Budget Impact Analysis—Principles of Good Practice: Report of the ISPOR 2012 Budget Impact Analysis Good Practice II Task Force

Citation: Sullivan SD, Mauskopf JA, Augustovski F, Jaime Caro J, Lee KM, Minchin M, Orlewska E, Penna P, Rodriguez Barrios JM, Shau WY. Budget impact analysis-principles of good practice: report of the ISPOR 2012 Budget Impact Analysis Good Practice II Task Force. Value Health. 2014 Jan-Feb;17(1):5-14. doi: https://doi.org/10.1016/j.jval.2013.08.2291

Recommendations for Reporting Format

Section/topic	Guidance for reporting	Reported in section
**Introduction**		
Objectives	The objective of the BIA should be clearly stated and tied to the study perspectives	Introduction
Epidemiology and management of health problem	Present information about the prevalence and incidence of the particular disease, disease severity, disease progression, undiagnosed or undertreated cases, and risk factors pertinent to estimating the budget impact	Introduction
Clinical impact	Consist of a brief description of the eligible population and existing management options and their efficacy and safety that are relevant to the design of the study of the BIA	Introduction
Economic impact	Include a brief description of previous BIAs in the condition of interest for another intervention and condition-specific treatment patterns and cost of-care studies	Not applicable (No previous BIA)
**Study Design and Methods**		
Patient population	Specify the eligible population for the new intervention	Methods<br>Sub-section: Eligible population and input assumptions<br>Table 1
Intervention mix	Contain a detailed description of the use and characteristics of each intervention in the current intervention mix and in the expected intervention mix after the introduction of the new intervention	Methods<br>Sub-section: Health service under assessment and its comparator<br>Figure 1 and 2
Time horizon	Should be presented and the choice(s) justified	Methods<br>Sub-section: Perspective and time horizon
Perspective	Identify the BIAs' perspective(s), the cost categories included, and the intended audience	Methods<br>Perspective and time horizon

For peer review only - http://bmjopen.bmj.com/site/about/guidelines.xhtml

ISPOR—The Professional Society for Health Economics and Outcomes Research

Section/topic	Guidance for reporting	Reported in section
Analytic framework description	Complete description of the structure of the BIA cost calculator or condition-specific cohort or individual simulation model	Methods<br>Sub-section: Eligible population and input assumptions
Input data	Input values used for the reported analyses, including alternative scenarios, should be presented	Methods<br>Sub-section: Cost input and data sources<br>Table 2
Data sources	The sources of data inputs should be described in detail	Methods<br>Sub-section: Cost input and data sources<br>Table 2
Data collection	The methods and processes for any primary data collection and data abstraction tasks not reported elsewhere should be described and explained.	Not applicable (secondary data)
Analyses	A description of the calculations used to complete the BIA should be provided. The choice of all the scenarios presented in the results should be documented and justified.	Methods<br>Sub-section: Computing framework and base-case analysis under budget impact analyses
Uncertainty	Uncertainty analysis methods should be described and justified	Methods<br>Sub-sections: Uncertainty and scenario analyses under budget impact analyses
**Results**	The budget impact should be presented for each budget period over the time horizon. Both budget period resource use and costs should be presented. The estimates of resource use should be listed in a table that shows the change in use for each time period reported in the BIA<br><br>The results of the uncertainty analyses and scenarios analyzed should be described and presented in figures or tables	Results<br>Table 3<br><br>Results<br>Figure 2
**Conclusions and Limitations**	State the main conclusions on the basis of the results of the BIA.<br>Report the main limitations regarding key issues such as design aspects including off-label use and adherence assumptions and the completeness and quality of data inputs and sources.	Discussion<br><br>Conclusion
**Inclusion of Graphics and Tables**		
Figure of the analytical framework	Flow diagrams or other visual depictions of the cost calculator or condition-specific cohort or individual simulation model are recommended to be included with the analytical framework description.	Supplementary material<br>Figure S1

For peer review only - http://bmjopen.bmj.com/site/about/guidelines.xhtml

ISPOR—The Professional Society for Health Economics and Outcomes Research

Section/topic	Guidance for reporting	Reported in section
Table of assumptions	All the major assumptions should be listed in a tabular form	Table 1
Tables of inputs	All the input parameter values and their data sources and<br>derivations should be presented in a tabular form	Table 2
Tables of outputs	All outputs should be presented in a tabular and/or graphical<br>Form	Table 3
Schematic representation of uncertainty analyses	Diagrams such as Tornado diagrams should be included along with the text on the results of the scenario analyses	Figure 3
Appendices and References	The appendices may cover literature search strategies, evidence summaries, intermediate results (e.g., of individual Delphi panel rounds), and the names and addresses of participating experts and investigators, for example.	Reference

For peer review only - http://bmjopen.bmj.com/site/about/guidelines.xhtml

CHEERS 2022 Checklist

Topic	No.	Item	Location where item is reported
**Title**			
	1	Identify the study as an economic evaluation and specify the interventions being compared.	Title page, Page 1
**Abstract**			
	2	Provide a structured summary that highlights context, key methods, results, and alternative analyses.	Abstract, Page 1
**Introduction**			
**Background and objectives**	3	Give the context for the study, the study question, and its practical relevance for decision making in policy or practice.	Introduction, Line 65-69
**Methods**			
**Health economic analysis plan**	4	Indicate whether a health economic analysis plan was developed and where available.	Not applicable
**Study population**	5	Describe characteristics of the study population (such as age range, demographics, socioeconomic, or clinical characteristics).	Methods, Line 123-152, Table 1
**Setting and location**	6	Provide relevant contextual information that may influence findings.	Methods, Line 79-92
**Comparators**	7	Describe the interventions or strategies being compared and why chosen.	Methods, Line 79-121, Figure 1 and 2
**Perspective**	8	State the perspective(s) adopted by the study and why chosen.	Methods, Line 199-202
**Time horizon**	9	State the time horizon for the study and why appropriate.	Methods, Line 204-205
**Discount rate**	10	Report the discount rate(s) and reason chosen.	Methods, Line 205-206
**Selection of outcomes**	11	Describe what outcomes were used as the measure(s) of benefit(s) and harm(s).	Not applicable

For peer review only - http://bmjopen.bmj.com/site/about/guidelines.xhtml

Topic	No.	Item	Location where item is reported
**Measurement of outcomes**	12	Describe how outcomes used to capture benefit(s) and harm(s) were measured.	Not applicable
**Valuation of outcomes**	13	Describe the population and methods used to measure and value outcomes.	Not applicable
**Measurement and valuation of resources and costs**	14	Describe how costs were valued.	Methods, Line 159-196
**Currency, price date, and conversion**	15	Report the dates of the estimated resource quantities and unit costs, plus the currency and year of conversion.	Methods, Line 73-76
**Rationale and description of model**	16	If modelling is used, describe in detail and why used. Report if the model is publicly available and where it can be accessed.	Not applicable
**Analytics and assumptions**	17	Describe any methods for analysing or statistically transforming data, any extrapolation methods, and approaches for validating any model used.	Methods, Line 211-221, Figure S1 (Supplementary Material)
**Characterising heterogeneity**	18	Describe any methods used for estimating how the results of the study vary for subgroups.	Not applicable
**Characterising distributional effects**	19	Describe how impacts are distributed across different individuals or adjustments made to reflect priority populations.	Not applicable
**Characterising uncertainty**	20	Describe methods to characterise any sources of uncertainty in the analysis.	Methods, Line 224-229, Figure S2 (Supplementary Material)
**Approach to engagement with patients and others affected by the study**	21	Describe any approaches to engage patients or service recipients, the general public, communities, or stakeholders (such as clinicians or payers) in the design of the study.	Methods, Line 233-235
**Results**			
**Study parameters**	22	Report all analytic inputs (such as values, ranges, references) including uncertainty or distributional assumptions.	Table 1 and 2, Table S2 (Supplementary Material)
**Summary of main results**	23	Report the mean values for the main categories of costs and outcomes of interest and summarise them in the most appropriate overall measure.	Results, Line 239-274, Table 3

For peer review only - http://bmjopen.bmj.com/site/about/guidelines.xhtml

Topic	No.	Item	Location where item is reported
**Effect of uncertainty**	24	Describe how uncertainty about analytic judgments, inputs, or projections affect findings. Report the effect of choice of discount rate and time horizon, if applicable.	Results, Line 277-295, Figure 3
**Effect of engagement with patients and others affected by the study**	25	Report on any difference patient/service recipient, general public, community, or stakeholder involvement made to the approach or findings of the study	Not applicable
**Discussion**			
**Study findings, limitations, generalisability, and current knowledge**	26	Report key findings, limitations, ethical or equity considerations not captured, and how these could affect patients, policy, or practice.	Discussion
**Other relevant information**			
**Source of funding**	27	Describe how the study was funded and any role of the funder in the identification, design, conduct, and reporting of the analysis	End of manuscript
**Conflicts of interest**	28	Report authors conflicts of interest according to journal or International Committee of Medical Journal Editors requirements.	End of manuscript

From: Husereau D, Drummond M, Augustovski F, et al. Consolidated Health Economic Evaluation Reporting Standards 2022 (CHEERS 2022) Explanation and Elaboration: A Report of the ISPOR CHEERS II Good Practices Task Force. Value Health 2022;25. doi:10.1016/j.jval.2021.10.008

For peer review only - http://bmjopen.bmj.com/site/about/guidelines.xhtml

BMJ Open

A budget impact analysis of a home-based colorectal cancer screening programme in Malaysia

Journal:	*BMJ Open*
Manuscript ID	bmjopen-2022-066925.R2
Article Type:	Original research
Date Submitted by the Author:	08-Feb-2023

Complete List of Authors:	Ngan, Tran; Queen's University Belfast, Centre for Public Health<br>Ramanathan, Kogila; Monash University Malaysia, Global Public Health, Jeffrey Cheah School of Medicine and Health Sciences; Monash University Malaysia, South East Asia Community Observatory<br>Saleh, Muhamad; Monash University Malaysia, South East Asia Community Observatory<br>Schliemann, Desiree ; Queen's University Belfast, Centre for Public Health<br>Ibrahim Tamin, Nor Saleha ; Ministry of Health Malaysia<br>Su, Tin ; Monash University Malaysia, Global Public Health, Jeffrey Cheah School of Medicine and Health Sciences; Monash University Malaysia, South East Asia Community Observatory<br>Donnelly, Michael; Queen's University Belfast, Centre for Public Health<br>O'Neill, Ciaran; Queen's University Belfast, Centre for Public Health

**Primary Subject Heading**:	Health economics
Secondary Subject Heading:	Global health, Public health, Oncology
Keywords:	Health policy < HEALTH SERVICES ADMINISTRATION & MANAGEMENT, ONCOLOGY, HEALTH ECONOMICS, PUBLIC HEALTH

SCHOLARONE™
Manuscripts

I, the Submitting Author has the right to grant and does grant on behalf of all authors of the Work (as defined in the below author licence), an exclusive licence and/or a non-exclusive licence for contributions from authors who are: i) UK Crown employees; ii) where BMJ has agreed a CC-BY licence shall apply, and/or iii) in accordance with the terms applicable for US Federal Government officers or employees acting as part of their official duties; on a worldwide, perpetual, irrevocable, royalty-free basis to BMJ Publishing Group Ltd ("BMJ") its licensees and where the relevant Journal is co-owned by BMJ to the co-owners of the Journal, to publish the Work in this journal and any other BMJ products and to exploit all rights, as set out in our licence.

The Submitting Author accepts and understands that any supply made under these terms is made by BMJ to the Submitting Author unless you are acting as an employee on behalf of your employer or a postgraduate student of an affiliated institution which is paying any applicable article publishing charge ("APC") for Open Access articles. Where the Submitting Author wishes to make the Work available on an Open Access basis (and intends to pay the relevant APC), the terms of reuse of such Open Access shall be governed by a Creative Commons licence – details of these licences and which Creative Commons licence will apply to this Work are set out in our licence referred to above.

Other than as permitted in any relevant BMJ Author's Self Archiving Policies, I confirm this Work has not been accepted for publication elsewhere, is not being considered for publication elsewhere and does not duplicate material already published. I confirm all authors consent to publication of this Work and authorise the granting of this licence.

For peer review only

A budget impact analysis of a home-based colorectal cancer screening programme in Malaysia

Tran Thu Ngan[1*], Kogila Ramanathan[2,3], Muhamad Raziq Bin Mohd Saleh[3], Désirée Schliemann[1], Nor Saleha Binti Ibrahim Tamin[4], Tin Tin Su[2,3], Michael Donnelly[1], Ciaran O'Neill[1]

[1] *Centre for Public Health, Queen's University Belfast, Belfast, UK*

[2] *Global Public Health, Jeffrey Cheah School of Medicine and Health Sciences, Monash University Malaysia, Selangor, Malaysia*

[3] *South East Asia Community Observatory (SEACO), Jeffrey Cheah School of Medicine and Health Sciences, Monash University Malaysia*

[4] *Ministry of Health Malaysia, Putrajaya, Malaysia*

* Correspondence to:

Tran Thu Ngan, PhD

Postal address: Centre for Public Health, Queen's University Belfast, Belfast, United Kingdom

Email: n.t.tran@qub.ac.uk

ORCID of the authors

Tran Thu Ngan : 0000-0003-2771-9878

Désirée Schliemann : 0000-0002-8746-3002

Ciaran O'Neill : 0000-0001-7668-3934

For peer review only

A budget impact analysis of a home-based colorectal cancer screening programme in Malaysia

Abstract

Objectives: The 2020-2022 research project '**C**olo**r**ectal **C**ancer **S**creening **I**ntervention for **M**alaysia' (CRC-SIM) evaluated the implementation of a home-based CRC screening pilot in Segamat District. This budget impact analysis (BIA) assessed the expected changes in health expenditure of the Malaysian Ministry of Health budget in the scenario where the pilot programme was implemented nationwide versus current opportunistic screening.

Design: Budget impact analysis. Assumptions and costs in the opportunistic and novel CRC screening scenarios were derived from a previous evaluation of opportunistic CRC screening in community health clinics across Malaysia and the CRC-SIM research project, respectively.

Setting: National level (with supplement analysis for district level). The BIA was conducted from the viewpoint of the federal government and estimated the annual financial impact over a period of five years.

Results: The total annual cost of the current practice of opportunistic screening was RM1,584,321 (~I$1,099,460; RM=Ringgit Malaysia; I$=International dollar) of which 80% (RM1,274,690 or ~I$884,587) was expended on the provision of opportunistic CRC to adults who availed of the service. Regarding the implementation of national CRC screening programme, the net budget impact in the 1st year was estimated to be RM107,631,959 (~I$74,692,546) and to reach RM148,485,812 (~I$103,043,589) in the 5th year based on an assumed increased uptake of 5% annually. The costs were calculated to be sensitive to the probability of adults who were contactable, eligible, and agreeable to participating in the programme.

Conclusions: Results from the BIA provided direct and explicit estimates of the budget changes to when implementing a population-based national CRC screening programme to aid decision making by health services planners and commissioners in Malaysia about whether such programme is affordable within given their budget constraint. The study also illustrates the use and value of the BIA approach in LMICs and resource-constrained settings.

Keywords: Colorectal cancer screening, budget impact analysis, home-based testing, global health, Malaysia

For peer review only - http://bmjopen.bmj.com/site/about/guidelines.xhtml

Strengths and limitations of this study

- The budget impact analysis (BIA) was used to evaluate the 'affordability' of colorectal cancer (CRC) screening programme in Malaysia within given budget constraint.
- Assumptions and cost inputs for modelling the budget impact were based on the actual costs and rates observed in Malaysia.
- The total cost of resources (=unit costs * number of users) for opportunistic screening and the CRC screening programme were compared to calculate the net budget impact.
- The BIA was conducted from the viewpoint of the federal government and only included costs and resource requirements relevant to this particular budget holder.
- The BIA could not and was not intended to provide answers to questions about whether or not the screening programme is good value for money (which can be answered by a cost-effectiveness analysis).

INTRODUCTION

Colorectal cancer (CRC) has the second highest incidence and mortality rate among all types of cancer in both sexes in Malaysia [1]. The age standardised incidence rate in 2012-2016 was 14.8 per 100,000 males and 11.1 per 100,000 females which appears to be stable compared to 2007-2011 [2]. In contrast, the proportion of CRC patients who are diagnosed at a late stage (i.e., stage III or IV) is increasing. Report from Ministry of Health Malaysia (MoHM) showed that the proportion of males with late stage CRC increased from 65.9% during 2007-2011 to 72.4% during 2012-2016; and from 65.2% to 73.1% for females [2]. The report did not give an explanation about this increasing trend though [2]. Late stage diagnosis negatively impacts survival rate; for example, the 5-year survival rates for cases diagnosed at stage I, II, III, and IV in 2002-2004 in Kuala Lumpur were 78.6%, 52.9%, 44.3%, and 9.3%, respectively [3]. Improved survival can be achieved by early detection through screening and the removal of premalignant polyps [4]. However, Malaysia currently does not have a population-based national CRC screening programme.

For peer review only - http://bmjopen.bmj.com/site/about/guidelines.xhtml

The Ministry of Health of Malaysia (MoHM) adopted the use of immunochemical faecal occult blood test (iFOBT) for opportunistic CRC screening at public health clinics since 2014 [5]. MoHM guidelines recommend screening for asymptomatic individuals aged 50-75 years old with average risk of CRC [6]. The uptake (number of patients screened/total eligible population) of this opportunistic screening tends to be very low. The annual average uptake during 2014-2018 was 0.5% while the 5-year cumulative uptake was 2.29% due to low awareness about CRC in general and CRC tests in particular, fear of the result, concern about the cost, and absence of a doctor's recommendation [5, 7]. Home-based iFOBT has been implemented in many high-income countries (HICs) to improve the accessibility and uptake of CRC screening [8]. In this context, the Southeast Asia Community Observatory (SEACO) at Monash University Malaysia and Queen's University Belfast (Northern Ireland) collaborated to conduct the research project, '**C**olo**r**ectal **C**ancer **S**creening **I**ntervention for **M**alaysia' (CRC-SIM) in 2020-2022. This project evaluated the implementation of a home-based CRC screening pilot in Segamat District. The uptake of the novel screening programme was 22%. The significantly higher uptake indicates the potential population wide impact if this screening approach (i.e., using home-based iFOBT and self-reporting test results) was scaled up. However, in order to aid public health decision making, there is a need to model a scaled-up version of the research-tested screening programme and, more specifically, gather insights about the total costs of programme implementation and how it might impact the MoHM budget. In other words, there is a need for a budget impact analysis (BIA).

Budget impact analysis was first introduced in 1998 by Mauskopf [9, 10]. Since then, BIA is gradually requested as a part of the health technology assessment (HTA) procedure by a few countries around the world such as Australia, Canada, the United States (the US), England, Ireland, Spain, Belgium, Poland, Israel, and Thailand [11]. Regarding BIA for colorectal cancer

For peer review only

(CRC) screening, a recent systematic review found six studies conducted in the UK, US, Belgium, and Australia [12]. We found two additional studies published in 2018 and 2019 from Spain and Thailand, respectively [13, 14]. Although results from these studies are not comparable as they were specific to each studied country, all studies were conducted to answer the question 'What is the budget impact of implementing a colorectal cancer screening/prevention programme compared with current usual care'. It is also the research question that the BIA in this study aims to answer. Specifically, the BIA assessed the expected changes in the health expenditure of MoHM budget as a result of implementing a population-based national CRC screening programme versus current opportunistic screening (or 'usual care'). It assessed the affordability of the screening programme given potential budget constraints.

METHODS

The conduct of this BIA and presentation of this paper followed the guidelines developed by the International Society for Pharmacoeconomics and Outcomes Research (ISPOR) Task Force [11, 15]. All costs are presented in local currency -the Malaysian Ringgit (RM)- and International Dollar (I$). RM was converted to I$ using purchasing power parity (PPP) conversion factors instead of market exchange rates. The PPP conversion rate of 1.441 was obtained from the IMF World Economic Outlook Database [16].

Health service under assessment and its comparator

The specific health service that was the focus of the BIA was a population-based screening programme for colorectal cancer using a self-rapid response iFOBT. The comparator was current or 'usual care' - opportunistic screening.

For peer review only - http://bmjopen.bmj.com/site/about/guidelines.xhtml

The BIA is predicated on the opportunistic screening programme being replaced by the new population-based screening programme (i.e., the two programmes would not be run in conjunction or in other words, the two scenarios in assessment are mutually exclusive). In each scenario, the patient pathway from the point when patients were invited for screening to receipt of a definitive diagnosis were identified and described. The screening procedure ends at the point of a patient receiving their iFOBT result with encouragement to attend hospital for a colonoscopy (if iFOBT is positive). It is important to note that the BIA included costs of screening and diagnosis (e.g., colonoscopy, biopsy) but not treatment. The BIA also did not address issues with respect to equity of access and uptake of services in either screening scenarios.

The patient pathways for the 'usual care' practice and the novel CRC screening programme are presented in Figure 1 and 2, respectively. In opportunistic screening practice, it is recommended or expected that individuals who are aged 50-75 years will be screened for CRC symptoms when they attend their local health clinic (for any health condition or problem). If they are asymptomatic and have an average risk of having CRC (based on family history), they are offered an iFOBT, followed by a colonoscopy if the iFOBT test was positive. If CRC is detected following a colonoscopy, the result is conveyed to a patient along with an explanation of the treatment plan or referral arrangement.

(Figure 1 is about here)

Details of the home-based screening intervention in CRC-SIM were published elsewhere [17]. Briefly, in the novel CRC screening programme, individuals aged 50-75 years were contacted, checked for eligibility, and invited to participate. A home-screening 'pack' was posted to eligible participants followed by two reminders. The test was performed at home by participants who took a photograph of the completed test and texted it to trained medical professionals who interpreted the photograph. Participants with positive iFOBT were referred for a colonoscopy at hospital.

For peer review only

For peer review only - http://bmjopen.bmj.com/site/about/guidelines.xhtml

(Figure 2 is about here)

There were two main differences between these patient pathways. Firstly, individuals within the target age group for screening were contacted directly and invited to participate in the novel CRC screening programme while in the situation of 'usual care', CRC screening was offered (if screening guideline recommendations were followed) only when members of the target group visited their clinic for some other health condition or problem. Secondly, the iFOBT was performed by doctors at health clinics in the 'usual care' pathway while in the novel CRC screening programme, participants self-tested in their home. Home-based testing generated additional stages in the pathways in relation to sending a test, reminding participants, taking a photo of a completed test, and sending it to programme officers and vice versa. The remaining stages of each pathway (e.g., being screened for eligibility, receiving a colonoscopy, and receiving a treatment plan) were the same across the two scenarios.

Eligible population and input assumptions

The target population for current opportunistic screening in Malaysia is individuals aged 50-75 years, regardless of sex. Due to the nature of home-based screening, the target population for the CRC screening programme was required to meet some additional inclusion criteria as presented in Figure 2. The number of individuals who presented and completed each stage was estimated using input assumptions.

Data about the population of Malaysia by age was taken from government reports (i.e., Department of Statistics, Malaysia) and from World Population Review [18, 19]. The total population was reported to be 32,676,786 in 2021, of which, 19% or 6,228,195 were aged 50-75 years old [18, 19].

For peer review only - http://bmjopen.bmj.com/site/about/guidelines.xhtml

For peer review only

In the 'usual care' – opportunistic screening pathway or scenario, all assumptions were derived from a study by Tamin NSI (2020) which was a 5-year evaluation of opportunistic CRC screening (and the use of stool-based tests) in community health clinics across Malaysia [5]. It was assumed that 0.482% of the eligible population would avail of CRC screening when they attended local health clinics for other conditions; and 9.21% of this proportion of tested patients would receive a positive result. Only 55.9% of patients in the study by Tamin availed of a colonoscopy after a positive iFOBT. CRC detection after colonoscopy investigation was 4.04%.

In the novel CRC screening programme, all assumptions were derived from the CRC-SIM research project. It was assumed that 50.51% of the eligible population would be contactable and meet all inclusion criteria to participate in the home-based screening programme; 52.27% of people who were eligible would agree to participate; 41.63% would perform the iFOBT and send a photo of a completed test to the programme officers; 18.01% of people who would be tested would receive a positive result; 41.07% would avail of colonoscopy after a positive iFOBT result; and CRC detection after colonoscopy investigation would be 4.35%.

Table 1 summarises details about the input assumptions that were used to estimate the number of individuals at each stage of the respective pathway: the opportunistic screening pathway and the CRC screening programme pathway.

For peer review only - http://bmjopen.bmj.com/site/about/guidelines.xhtml

Table 1: Input assumptions used to estimate the population at each stage of the patient pathways

Stage in pathway	Opportunistic screening scenario (Current practice)		Population-based CRC programme screening scenario (Proposed practice)	
	Assumption*	No. of individuals	Assumption**	No. of individuals
Total population (all ages)	NA	32,676,786	NA	32,676,786
Target population (aged 50-75)	19.06%	6,228,195	19.06%	6,228,195
Eligible population (met all inclusion criteria)	100%	6,228,195	50.51%	3,146,020
Availed of/agreed to take CRC screening	0.482%	30,020	52.27%	1,644,561
Needed 1st reminder to return the iFOBT result (among those agreed to participate)	NA	NA	78.71%	1,294,514
Needed 2nd reminder to return the iFOBT result (among those received 1st reminder)	NA	NA	88.10%	1,140,405
Returned iFOBT result (among those agreed to participate)	100%	30,020	41.63%	684,683
Received iFOBT positive result	9.21%	2,765	18.01%	123,287
Availed of colonoscopy after positive iFOBT	55.9%	1,546	41.07%	50,636
CRC detection after colonoscopy investigation	4.04%	62	4.35%	2,202

CRC: Colorectal cancer; iFOBT: Immunochemical faecal occult blood test; NA: Not applicable; No: Number

** The assumptions were derived from a study of Tamin NSI (2020) which was a 5-year evaluation of using stool-based test for opportunistic CRC screening in primary health institutions across Malaysia [5].*

*** The assumptions were derived from the Colorectal Cancer Screening Intervention for Malaysia (or CRC-SIM research project) in Segamat District, conducted by Queen's University Belfast, Monash University, and Southeast Asia Community Observatory (SEACO) in 2021.*

For peer review only - http://bmjopen.bmj.com/site/about/guidelines.xhtml

For peer review only

Cost input and data sources

In the opportunistic screening scenario, the total cost comprised the cost of:

(i) performing screening (e.g., asking for symptoms, family history, and collecting the sample)

(ii) processing stool specimens

(iii) interpreting test results and

(iv) conveying a definitive diagnosis to patients (include explaining treatment plan or referral arrangements)

In the CRC programme screening scenario, the total cost comprised the costs of:

(i) contacting potential participants

(ii) delivering iFOBT test kits (including cost of the test, postage, print materials, and sending video instruction)

(iii) sending a reminder to participants (up to 2 times, by text message and phone call)

(iv) interpreting and conveying results to participants and

(v) following-up patients with positive iFOBT but did not take colonoscopy in order to encourage them to avail of the colonoscopy

These costs were calculated by multiplying the time allocated for the completion of each task with the salary cost of the person who undertakes each task plus cost of consumables. Table 2 shows the unit cost for each cost element, related assumptions, and data sources.

For peer review only

For peer review only - http://bmjopen.bmj.com/site/about/guidelines.xhtml

Table 2: Resources and unit costs

Currency: Malaysian ringgit (RM)

Cost element	Unit cost *(Per screen) RM (I$)*	Assumptions	Source
Current practice (opportunistic screening)			
Performing screening (asking for symptoms, family history, referral) and taking sample	5.58	20 min x salary RM2947/month	1
Processing stool specimens	1.70	10 min x salary RM1797/month	2
Interpreting the test results	2.79	10 min x salary RM2947/month	1
Conveying a definitive diagnosis to patients (along with explaining treatment plan or referral etc.)	8.37	30 min x salary RM2947/month	1
Proposed practice (Population-based CRC screening programme)			
Contact eligible individuals - agreed to participate	0.98	7.1 min x (salary RM1440/month + mobile package RM20/month)	3
Contact eligible individuals - rejected/excluded to participate	0.47	3.4 min x (same as above)	3
iFOBT rapid test kit	6.90		3
Print materials (instruction leaflet, explanatory statement)	1.10	90 cents for colour print + 20 cent for black & white print	3
Postage (stamps, etc.)	5.35		3
Sending video through WhatsApp	0.41	3 min x (salary RM1440/month + mobile package RM20/month)	3
Sending reminder text message	0.41	3 min x (same as above)	3
Reminder call	0.28	2 min x (same as above)	3
Interpreting the test kit result	1.70	10 min x salary RM1797/month	3
Sending text message to inform patient of negative result	0.45	2 min x (salary RM2350/month + mobile package RM20/month)	3
Calling patient to inform him/her of positive result	0.67	3 min x (same as above)	3
Preparing and sending referral letter to patient/clinic	1.12	5 min x (same as above)	3
Follow up effort	6.73	30 min x (same as above)	3
Developing communication materials, **one-off cost**	6,063	Communication materials do not change in 5 years	3

Currency: Malaysian ringgit (RM)

Cost element	Unit cost *(Per screen) RM (I$)*	Assumptions	Source
Training for data collectors*, **one-off cost**<br>* *Data collectors are those employed by the programme to (i) contact potential participants, (ii) deliver iFOBT test kits, and (iii) send a reminder to participants*	109,703	+ 1 day training (virtual using Zoom)<br>+ 1 trainer for maximum 25 trainees<br>+ 1 data collector* is needed for every target population of 400<br>+ Cost=1-day-salary of trainer/trainees x number of trainer/trainees<br>+ No retraining in 5 years	3
Same in both scenarios/practices			
Colonoscopy (including polyps removal and/or biopsy if needed)	200		
Consumables – stool container, gloves, mask, plastic waste bag and disposal of materials from the test	10.80	RM8636.7/800 sets	3

iFOBT: Immunochemical faecal occult blood test; RM: Malaysian ringgit

Source:

1. *Public Services Commission of Malaysia. Medical Officer Grade UD41. Accessed at https://www.spa.gov.my/spa/laman-utama/gaji-syarat-lantikan-deskripsi-tugas/ijazah-sarjana-phd/pegawai-perubatan-gred-ud41*
2. *Public Services Commission of Malaysia. Medical laboratory technologist Grade U29. Accessed at https://www.interactive.jpa.gov.my/ezskim/klasifikasi/perbekalanskim.asp?id_skim=3LU03*
3. *Colorectal Cancer Screening Intervention for Malaysia (or CRC-SIM research project) in Segamat District, conducted by Queen's University Belfast, Monash University, and Southeast Asia Community Observatory (SEACO) in 2021*

In the current practice of opportunistic screening, doctors were consulted about the estimated time to perform each stage in the pathway. The monthly salary of a general doctor and a medical laboratory technologist was based on the rate published by the Public Services Commission of Malaysia [20, 21]. These rates were RM2,947 (~I$2,045) and RM1,797 (~I$1,247), respectively. In the novel CRC screening programme, the time to perform each stage in the pathway, salary of personnel, and costs of material resources (e.g., rapid kit test, consumables, postage, printing materials) were based on the time and expenditure observed in the CRC-SIM research project. All costs were calculated per screen except the cost of training and the cost of developing communication materials which were one-off costs based on the assumption that communication materials would not change, and no re-training would be needed within 5 years.

For peer review only - http://bmjopen.bmj.com/site/about/guidelines.xhtml

It was assumed (based on the experience of operating the screening programme during the CRC-SIM project) that one data collector (i.e., those employed by the programme to (i) contact potential participants, (ii) deliver iFOBT test kits, and (iii) send a reminder to participants) would be needed for every 400 people in the target population. Training would last one day and would be delivered virtually; thus, the cost of training equalled (1-day-salary of trainer x number of trainer) + (1-day-salary of trainees x number of trainees/data collectors).

Perspective and time horizon

The BIA was conducted from the viewpoint of the federal government which finances Malaysia's public health system [22]. Only those costs and resource requirements relevant to the budget holder were included in the analysis. For example, the out-of-pocket expenditure incurred by patients were excluded.

The analysis estimated the annual financial impact over a period of five years as recommended in the guidelines [11, 23]. Costs were not discounted given that the BIA methodology reports the costs for each year in which they occur rather than a net present value [11].

Budget impact analyses

Computing framework and base-case analysis

The BIA used a cost calculator programmed in Microsoft Excel, following the costing template[1] produced by the National Institute for Health and Care Excellence in the UK (NICE). The template was modified to fit the programme under assessment. The cost calculator

[1] The template can be freely downloaded at https://www.nice.org.uk/Media/Default/About/what-we-do/our-programmes/evidence-standards-framework/budget-impact-template.xlsx

For peer review only - http://bmjopen.bmj.com/site/about/guidelines.xhtml

228 approach is recommended by guidelines as it is easy for stakeholders to understand and
229 replicate the results [11].
230
231 First, the number of individuals who completed each stage was estimated (Table 1). The
232 resources that were used at each stage of the respective pathways (in opportunistic screening
233 and the novel CRC screening programme) were listed along with their unit costs (i.e., cost of
234 each resource per person) (Table 2). Unit costs were multiplied by number of users to give the
235 total cost of resources for each scenario. The net budget impact was calculated as the difference
236 in cost between opportunistic screening and the CRC screening programme. Visual depiction
237 of the cost calculator is shown in Supplementary Material, Figure S1.
238
239 ***Uncertainty and scenario analyses***
240 The input assumptions (that were used to estimate the number of individuals at each stage of
241 the respective pathway) and the cost inputs were varied, and then the impact of these changes
242 in relation to the results was analysed to investigate the sensitivity of the budget impact results
243 to variations in individual input. As recommended by Gray et al. (2011), the range of variation
244 regarding parameters for which data sources about dispersion were unavailable were ±20% of
245 the base case [24].
246
247 **Patient and public involvement**
248 It was not appropriate or possible to involve patients or the public in the design, or conduct, or
249 reporting, or dissemination plans of our research as this type of study is a secondary analysis
250 of data from a payer perspective (Ministry of Health Malaysia).
251

For peer review only

For peer review only - http://bmjopen.bmj.com/site/about/guidelines.xhtml

RESULTS

Base-case analysis

The total annual cost of the current practice of opportunistic screening is RM1,584,321 (~I$1,099,460), of which 80% (RM1,274,690 ~ I$884,587) was for providing opportunistic CRC to adults who availed of the service. Costs of providing colonoscopy (including polyps removal and/or biopsy if needed) after receipt of a positive iFOBT and conveying definitive diagnosis to patients (along with explaining treatment plan or referral etc.) after the outcome of the colonoscopy were RM309,108 (~I$214,509) and RM523 (~I$363), respectively.

The total annual cost over a 5-year period of the proposed practice (i.e., CRC screening programme) is shown in Table 3. It was assumed that the number of people who would agree to participate in the programme would increase by 5% each year (in consideration of health promotion activities as well as information flows including word of mouth between participants). Therefore, the financial impact would also increase accordingly.

For peer review only

For peer review only - http://bmjopen.bmj.com/site/about/guidelines.xhtml

Table 3: Annual cost of proposed practice (i.e., CRC screening programme)

Currency: Malaysian ringgit (RM) and International Dollar (I$)

Proposed practice	Year 1<br>RM *(I$)*	Year 2<br>RM *(I$)*	Year 3<br>RM *(I$)*	Year 4<br>RM *(I$)*	Year 5<br>RM *(I$)*
Contacting adults who are eligible for CRC screening programme (i.e., aged 50-75) and screen for eligibility of participating	2,320,148<br>*(1,610,096)*	2,320,148<br>*(1,610,096)*	2,320,148<br>*(1,610,096)*	2,320,148<br>*(1,610,096)*	2,320,148<br>*(1,610,096)*
Providing iFOBT test to adults who agreed to participate in CRC screening programme after being invited	93,654,886<br>*(64,992,981)*	102,612,907<br>*(71,209,512)*	111,570,928<br>*(77,426,043)*	120,528,949<br>*(83,642,574)*	129,486,970<br>*(89,859,105)*
Providing 1st reminder to participants	536,929<br>*(372,609)*	588,286<br>*(408,248)*	639,643<br>*(443,888)*	690,999<br>*(479,527)*	742,356<br>*(515,167)*
Providing 2nd reminder to participants	315,339<br>*(218,833)*	345,501<br>*(239,765)*	375,663<br>*(260,696)*	405,825<br>*(281,627)*	435,987<br>*(302,559)*
Interpreting returned iFOBT samples	1,165,129<br>*(808,556)*	1,276,572<br>*(885,893)*	1,388,016<br>*(963,231)*	1,499,460<br>*(1,040,569)*	1,610,903<br>*(1,117,906)*
Conveying result through message to participants with iFOBT negative result	251,990<br>*(174,872)*	276,093<br>*(191,598)*	300,196<br>*(208,324)*	324,298<br>*(225,050)*	348,401<br>*(241,777)*
Preparing and sending referral letter and calling participants with iFOBT POSITIVE result	221,356<br>*(153,613)*	242,529<br>*(168,306)*	263,701<br>*(182,999)*	284,874<br>*(197,692)*	306,046<br>*(212,384)*
Following up participants who DID NOT take colonoscopy after positive iFOBT	489,158<br>*(339,457)*	535,945<br>*(371,926)*	582,733<br>*(404,394)*	629,520<br>*(436,863)*	676,308<br>*(469,332)*
Providing colonoscopy (including polyps removal and/or biopsy if needed) to participants with positive iFOBT	10,127,147<br>*(7,027,861)*	11,095,801<br>*(7,700,070)*	12,064,455<br>*(8,372,280)*	13,033,109<br>*(9,044,489)*	14,001,764<br>*(9,716,700)*
Conveying definitive diagnosis to patients (along with explaining treatment plan or referral etc.) after the colonoscopy	18,432<br>*(12,791)*	20,195<br>*(14,015)*	21,958<br>*(15,238)*	23,721<br>*(16,461)*	25,484<br>*(17,685)*
Capital costs (Developing communication materials + Training for data collectors)	115,766<br>*(80,337)*	115,766<br>*(80,337)*	115,766<br>*(80,337)*	115,766<br>*(80,337)*	115,766<br>*(80,337)*
**Total cost of proposed practice**	**109,216,279**<br>*(75,792,005)*	**119,429,743**<br>*(82,879,766)*	**129,643,206**<br>*(89,967,527)*	**139,856,670**<br>*(97,055,288)*	**150,070,133**<br>*(104,143,049)*

CRC: Colorectal cancer; iFOBT: Immunochemical faecal occult blood test

For peer review only - http://bmjopen.bmj.com/site/about/guidelines.xhtml

Similar to opportunistic screening, the cost to provide iFOBT to the eligible population who availed of the service accounted for 86% of the total cost of the proposed CRC screening programme. The second most costly component was the provision of colonoscopy (including polyps removal and/or biopsy if needed) to patients with an iFOBT positive result, at 9% of the total cost. The remaining nine cost components such as contacting potential participants, reminding participants to send photograph of iFOBT result, conveying diagnosis to participants and the follow-up effort added only up to 5% of the total cost.

The net budget impact in the 1st year of implementing CRC screening programme would be RM107,631,959 (~I$74,692,546 which equalled the total cost of future practice minus the total cost of current practice). The impact increases each year as the number of people who agree to participate in the programme increase, reaching RM117,845,422 (~I$81,780,307) in year 2, RM128,058,885 (~I$88,868,067) in year 3, RM138,272,349 (~I$ 95,955,829) in year 4, and RM148,485,812 (~I$103,043,589) in year 5.

The net budget impact of providing and delivering the CRC screening programme over the 5-year timeframe for each state in Malaysia (calculated according to the population size of each state) can be accessed in Supplementary Material, Table S1. These estimates aid service planning decisions if the novel pilot programme is implemented in one or more of these states before being scaled up into nationwide programme.

Uncertainty and scenario analyses

The tornado diagram in Figure 3 shows the change to net budget impact when assumptions and cost inputs were varied. It presents the results of multiple univariate sensitive analyses on key inputs that exert the most influence on the net budget impact (See Table S2, Supplementary

For peer review only - http://bmjopen.bmj.com/site/about/guidelines.xhtml

Material for results of multiple univariate sensitive analysis on all inputs). These inputs include the probability of (i) making successful contact with adults about the CRC screening programme, (ii) adults agreeing to participate, (iii) adults being eligible to participate in the programme, and (iv) the cost of consumables that are required to take a stool sample. The first three inputs influence the number of individuals who are present at each stage of the patient pathway.

(Figure 3 is about here)

The net budget impact would increase from RM107 million to RM130 million (~I$74-90 million) if there was a 20% increase in (i) the probability of adults who were contactable (from a contact list of people aged 50-75 years old) or (ii) the probability of adults agreeing to participate in the CRC screening programme or (iii) the probability of adults being eligible for the programme (i.e., aged 50-75 years old; having no symptoms of CRC, a smartphone, and WhatsApp; resident within programme area; and did not have colonoscopy this year). In other words, a 20% increase in each one of these factors would require an additional RM23 million (~I$16 million) to be budgeted for the programme. Likewise, a 20% increase in the cost of the consumables that are required for taking stool samples would mean that the programme would cost RM15 million (~I$10 million) more than the originally calculated total cost.

DICUSSION

The result of this analysis provides information to guide public health service planners and commissioners in their decisions about an alternative CRC screening strategy i.e., a population-based CRC screening programme using home-based iFOBT compared to current opportunistic screening. It concluded that the net budget impact in the 1st year of implementing a CRC screening programme of this kind would be RM107,631,959 (~I$74,692,546). The impact would increase by year due to increase in uptake and would reach RM148,485,812 (~I$103,043,589) in the 5th year of implementation. This analytical approach and the results of

For peer review only

this analysis are presented as aids to better decision making by MoHs and stakeholders in lower-middle-income countries (LMICs) about health programme planning and in this particular illustrative case to the MoHM regarding the degree to which the proposed CRC screening programme is affordable.

The total budget that was allocated to the MoHM in 2022 was RM32.4 billion (~I$22.5 million) [25]. Spending on prevention and public health services in 2009 was reported to be RM1.6 billion (~I$1.1 million) [22]. More recent data and information about the size of the budget that is allocated to cancer screening is not available. As such, it is estimated that the net budget impact of implementing a CRC screening programme would account for between 7-10% of the total budget for prevention and public health services. This represents a significant proportion of the overall budget allocated for prevention programmes/interventions.

The key factor in the implementation of a population-based screening programme/service or the factor that has biggest impact on the budget is the size of the population who use the service. The degree of accuracy regarding population size estimates is related closely to the cost estimates in the budget. It is important for service planners to keep this point in mind and to take into account an increase in uptake and the impact of such an increase. Therefore, in the case of the CRC programme presented here, we assumed a 5% increase annually in uptake and calculated the net budget impact. The net budget impact can be recalculated according to the actual change in uptake after the programme is implemented.

Budget impact analysis is an economic assessment that is used to estimate the changes in expenditure of a specific budget holder if a new health technology/programme is implemented [11]. As such, BIA complements other health economic evaluation methods such as cost-

For peer review only

For peer review only - http://bmjopen.bmj.com/site/about/guidelines.xhtml

effectiveness analysis (CEA) to provide a comprehensive economic assessment of a health care intervention to decision makers [11]. A BIA aids decision making by health service planners and commissioners about whether an intervention or programme is affordable within given budget constraints while a CEA informs decisions about whether an intervention is good value for money [11, 26]. BIA and its pragmatic approach is an ideal method when a situation calls for an evaluation of 'affordability' which is of central importance in LMICs and, arguably, is the key concern of whoever is in charge of managing a health care budget [27, 28].

It could well be that savings in earlier treatment would counterbalance the additional budget impact. Likewise, reduction in travel and time costs of participant while using home-based screening would reduce the total costs of the screening programme from a societal perspective. However, a BIA is not intended to provide answers to questions about whether or not, in this context, the screening programme is good value for money as it does not take into account the potential improvements in outcomes (e.g., increase quality-related life years) and savings from lower treatment costs for CRC diagnosed at earlier stages. The conduct of other types of economic evaluations such as a cost-effectiveness analysis would be required to provide a complete and comprehensive set of evidence for decision makers.

Finally, the conduct of BIA in this paper has some limitations. First, assumptions and cost inputs for the CRC screening programme were based on the costs and rates that were observed in the CRC-SIM research project. Due to unavailability of data about dispersion of the parameters, the used range of variation (±20% of the base case) may overestimate the uncertainty and suggests that the next step for further research is a CEA where parameter uncertainty is investigated with actual data. The project was conducted in only one district (Segamat); and the distribution of three main ethnic groups (i.e., Malay, Chinese, Indian) in

For peer review only

For peer review only - http://bmjopen.bmj.com/site/about/guidelines.xhtml

the project differed from the proportions that have been reported nation-wide (72%:24%:3% vs 62%:21%:6%, respectively). Therefore, it is important to be mindful of the possibility that the assumptions and inputs (based on the project) may not be representative for, or read across to, the whole population of Malaysia. Likewise, it is important to bear in mind that our findings do not include the perspective of other payers and may not generalise to other settings. The results are related directly to the context of the Malaysian health system and the epidemiology of CRC in the country though they are illustrative of the positive contribution of the BIA methodology and approach.

CONCLUSIONS

This study employed a BIA methodology to analyse the costs of a novel CRC screening programme using home-based iFOBT and mHealth versus the current opportunistic screening. The findings estimated the net budget impact of implementing a population-based national CRC screening programme in Malaysia. The modelling estimations are important considerations for health authorities when they are required to decide the affordability of implementing a programme and to aid budgetary planning as well as decision making, generally, about implementation. Our study illustrates the use and value of the BIA approach in LMICs and resource-constrained settings.

Abbreviations

BIA	Budget impact analysis
CEA	Cost-effectiveness analysis
CRC	Colorectal cancer
CRC-SIM	Colorectal Cancer Screening Intervention for Malaysia

For peer review only - http://bmjopen.bmj.com/site/about/guidelines.xhtml

iFOBT	Immunochemical faecal occult blood test
I$	International Dollar
ISPOR	The Professional Society for Health Economics and Outcomes Research
MoHM	Ministry of Health of Malaysia
NICE	National Institute for Health and Care Excellence
RM	Malaysian ringgit
SEACO	Southeast Asia Community Observatory
UK	The United Kingdom

DECLARATIONS

Research ethics approval. Not applicable (This study does not involve human participants).

Funding. This research was supported by the Medical Research Council (UK) Global Challenges Research Fund (MR/S014349/1) and the National Medical Research Register Malaysia (NMRR ID-21-02045-O7G). The funder of the study had no role in study design, data collection, data analysis, data interpretation, or writing of the report.

Competing interests. The authors have no conflicts of interest to declare that are relevant to the content of this article.

Availability of data and material. All data generated or analysed during this study are included in this published article.

Authors' contributions. TTN: Methodology, Formal Analysis, Writing – Original draft, Writing – Review & Editing. KR, MRS, and DS: Data Curation, Investigation, Writing – Review & Editing. ST: Resources, Writing – Review & Editing. TTS, and MD: Conceptualization, Funding acquisition, Writing – Review & Editing, Supervision. CON: Methodology, Formal Analysis, Writing – Review & Editing, Supervision.

For peer review only

REFERENCE

1. Global Cancer Observatory: Cancer Today [Internet]. International Agency for Research on Cancer. 2020 [cited 12 April 2021]. Available from: http://gco.iarc.fr/today.

2. Malaysian National Cancer Registry Report 2012-2016 [Internet]. Ministry of Health Malaysia. 2020 [cited April 12, 2022].

3. Veettil SK, Lim KG, Chaiyakunapruk N, Ching SM, Abu Hassan MR. Colorectal cancer in Malaysia: Its burden and implications for a multiethnic country. Asian Journal of Surgery. 2017;40(6):481-9.

4. Rawla P, Sunkara T, Barsouk A. Epidemiology of colorectal cancer: incidence, mortality, survival, and risk factors. Prz Gastroenterol. 2019;14(2):89-103.

5. Tamin NSI, Razalli KA, Sallahuddin SN, Chan HK, Hassan MRA. A 5-year evaluation of using stool-based test for opportunistic colorectal cancer screening in primary health institutions across Malaysia. Cancer epidemiology. 2020;69:101829.

6. Ministry of Health Malaysia. Clinical Practice Guidelines – Management of Colorectal Carcinoma. Putrajaya: Malaysia Health Technology Assessment Section (MaHTAS); 2017.

7. Schliemann D, Ramanathan K, Ibrahim Tamin NSB, Neill C, Cardwell CR, Ismail R, et al. Implementation of a colorectal cancer screening intervention in Malaysia (CRC-SIM) in the context of a pandemic: study protocol. BMJ Open. 2022;12(9):e058420.

8. Issa IA, Noureddine M. Colorectal cancer screening: An updated review of the available options. World journal of gastroenterology. 2017;23(28):5086-96.

9. Mauskopf J. Prevalence-Based Economic Evaluation. Value in Health. 1998;1(4):251-9.

10. Trueman P, Drummond M, Hutton J. Developing guidance for budget impact analysis. Pharmacoeconomics. 2001;19(6):609-21.

11. Sullivan SD, Mauskopf JA, Augustovski F, Jaime Caro J, Lee KM, Minchin M, et al. Budget impact analysis-principles of good practice: report of the ISPOR 2012 Budget Impact Analysis Good Practice II Task Force. Value Health. 2014;17(1):5-14.

12. Jahn B, Todorovic J, Bundo M, Sroczynski G, Conrads-Frank A, Rochau U, et al. Budget Impact Analysis of Cancer Screening: A Methodological Review. Appl Health Econ Health Policy. 2019;17(4):493-511.

13. Arrospide A, Idigoras I, Mar J, de Koning H, van der Meulen M, Soto-Gordoa M, et al. Cost-effectiveness and budget impact analyses of a colorectal cancer screening programme in a high adenoma prevalence scenario using MISCAN-Colon microsimulation model. BMC Cancer. 2018;18(1):464.

14. Phisalprapa P, Supakankunti S, Chaiyakunapruk N. Cost-effectiveness and budget impact analyses of colorectal cancer screenings in a low- and middle-income country: example from Thailand. Journal of medical economics. 2019;22(12):1351-61.

15. Mauskopf JA, Sullivan SD, Annemans L, Caro J, Mullins CD, Nuijten M, et al. Principles of good practice for budget impact analysis: report of the ISPOR Task Force on good research practices--budget impact analysis. Value Health. 2007;10(5):336-47.

16. World Economic Outlook Databases [Internet]. 2022 [cited April 13]. Available from: https://www.imf.org/en/Publications/SPROLLS/world-economic-outlook-databases#sort=%40imfdate%20descending.

17. Budget impact analysis of a population-based screening programme for colorectal cancer in Malaysia: technical report of a modelling study [Internet]. Centre for Public Health, Queen's University Belfast. 2022 [cited July 1]. Available from: https://pure.qub.ac.uk/en/publications/budget-impact-analysis-of-a-population-based-screening-programme-.

18. Malaysia Population Pyramid 2022 [Internet]. World Population Review. [cited Jan 21, 2022]. Available from: https://worldpopulationreview.com/countries/malaysia-population.
19. Demographic Statistics Factsheet [Internet]. Department Statistics of Malaysia. 2021 [cited Jan 21, 2022]. Available from: https://www.dosm.gov.my/v1/index.php?r=column/cthemeByCat&cat=430&bul_id=N05ydDRXR1BJWVlTdDY4TldHd253dz09&menu_id=L0pheU43NWJwRWVSZklWdzQ4TlhUUT09.
20. Medical Officer Grade UD41 [Internet]. Public Services Commission of Malaysia. [cited January 27, 2022]. Available from: https://www.spa.gov.my/spa/laman-utama/gaji-syarat-lantikan-deskripsi-tugas/ijazah-sarjana-phd/pegawai-perubatan-gred-ud41.
21. Medical laboratory technologist Grade U29 [Internet]. Public Services Commission of Malaysia. [cited January 27, 2022]. Available from: https://www.interactive.jpa.gov.my/ezskim/klasifikasi/perbekalanskim.asp?id_skim=3LU03.
22. Jaafar S, Noh KM, Muttalib KA, Othman NH, Healy J. Malaysia Health System Review. Asia Pacific Observatory on Health Systems and Policies; 2013.
23. Health Information and Quality Authority. Guidelines for the budget impact analysis of health technologies in Ireland. 2018.
24. M.Gray A, Clarke PM, Wolstenholme JL, Wordsworth S. Applied Methods of Cost-effectiveness Analysis in Health Care. Gray AM, Briggs A, editors. New York: Oxford University Press; 2011.
25. Ministry of Finance Malaysia. Budget 2022: RM32.4 billion allocation for MOH. Official Portal of Ministry of Finance Malaysia. 2021 [cited 2022 April 13]. Available from: https://www.mof.gov.my/portal/en/news/press-citations/budget-2022-rm32-4-billion-allocation-for-moh.
26. Leelahavarong P. Budget Impact Analysis. J Med Assoc Thai. 2014;97(Suppl. 5):S65-S71.
27. Orlewska E, Gulácsi L. Budget-Impact Analyses: A Critical Review of Published Studies. PharmacoEconomics. 2009;27(10):807-27.
28. Garattini L, van de Vooren K. Budget impact analysis in economic evaluation: a proposal for a clearer definition. The European Journal of Health Economics. 2011;12(6):499.

Figure Legends and Tables

Table 1 Input assumptions used to estimate the population at each stage of the patient pathways

Table 2 Resources and unit costs

Table 3 Annual cost of proposed practice (i.e., CRC screening programme)

Figure 1 Patient pathways in 'usual care' practice - opportunistic screening for CRC

Figure 2 Patient pathway in population-based CRC screening programme

Figure 3 Results of multiple univariate sensitive analyses showing key factors that exert most influence the net budget impact

For peer review only - http://bmjopen.bmj.com/site/about/guidelines.xhtml

Supplementary material

Figure S1 Visual depiction of the budget impact cost calculator

Table S1 Net budget impact of CRC screening programme over 5-year timeframe, by state

Table S2 Sensitivity of the total budget impact of CRC screening programme to changes in each variable individually

For peer review only

For peer review only - http://bmjopen.bmj.com/site/about/guidelines.xhtml

Figure 1: Patient pathway in 'usual care' practice - opportunistic screening for CRC

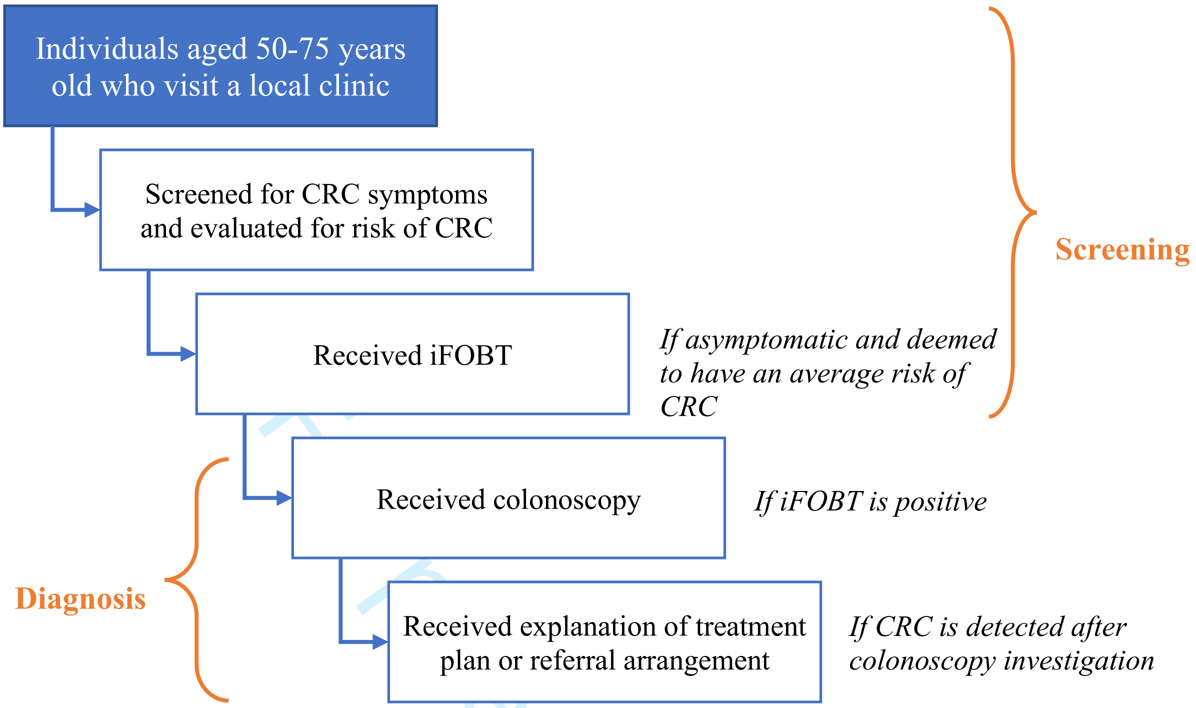

For peer review only

Figure 2: Patient pathway in population-based CRC screening programme

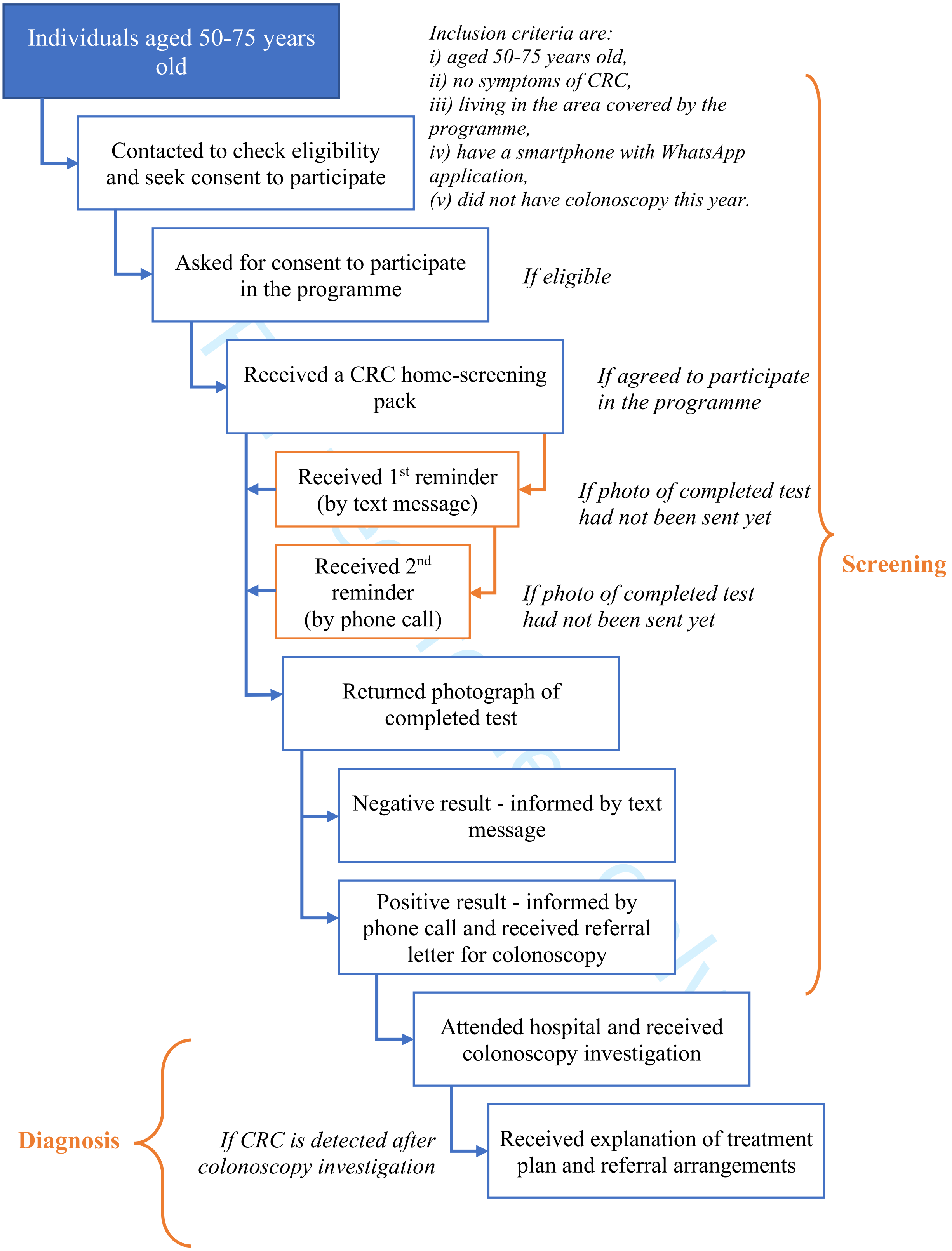

Figure 3: Results of multiple univariate sensitive analyses showing key factors that exert most influence the net budget impact

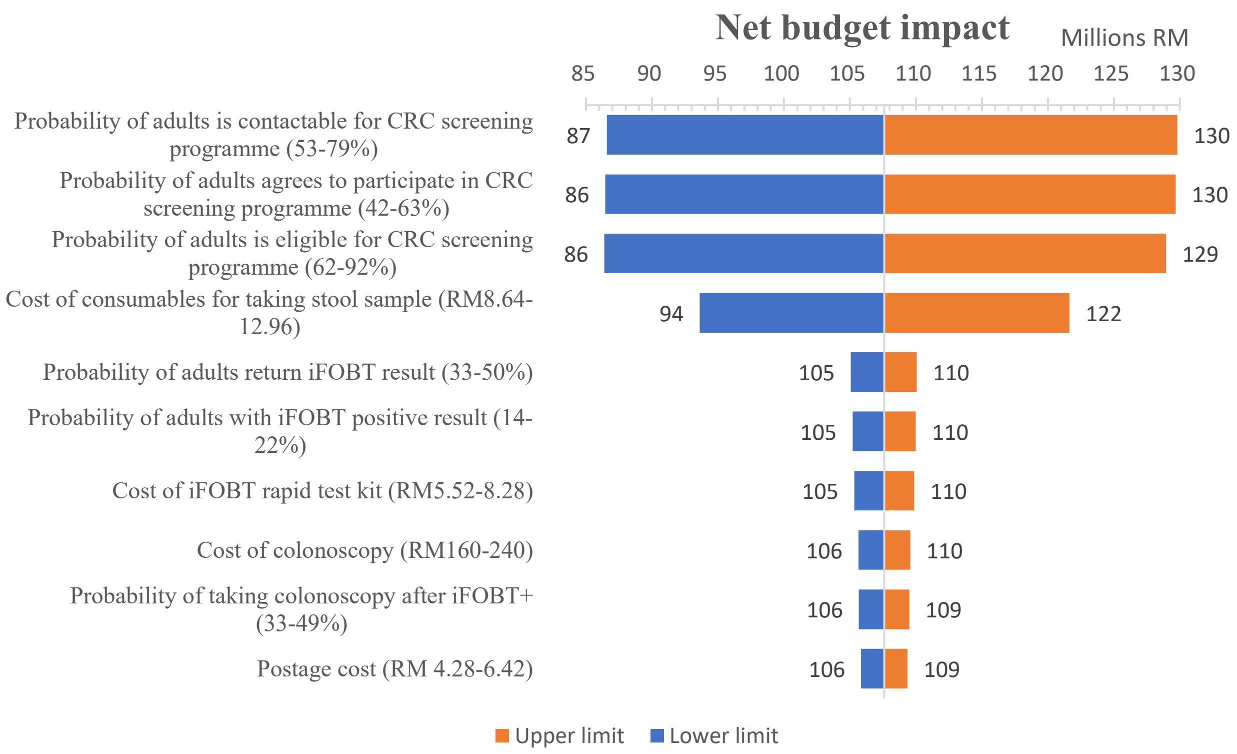

Supplementary material

Figure S1: Visual depiction of the budget impact cost calculator

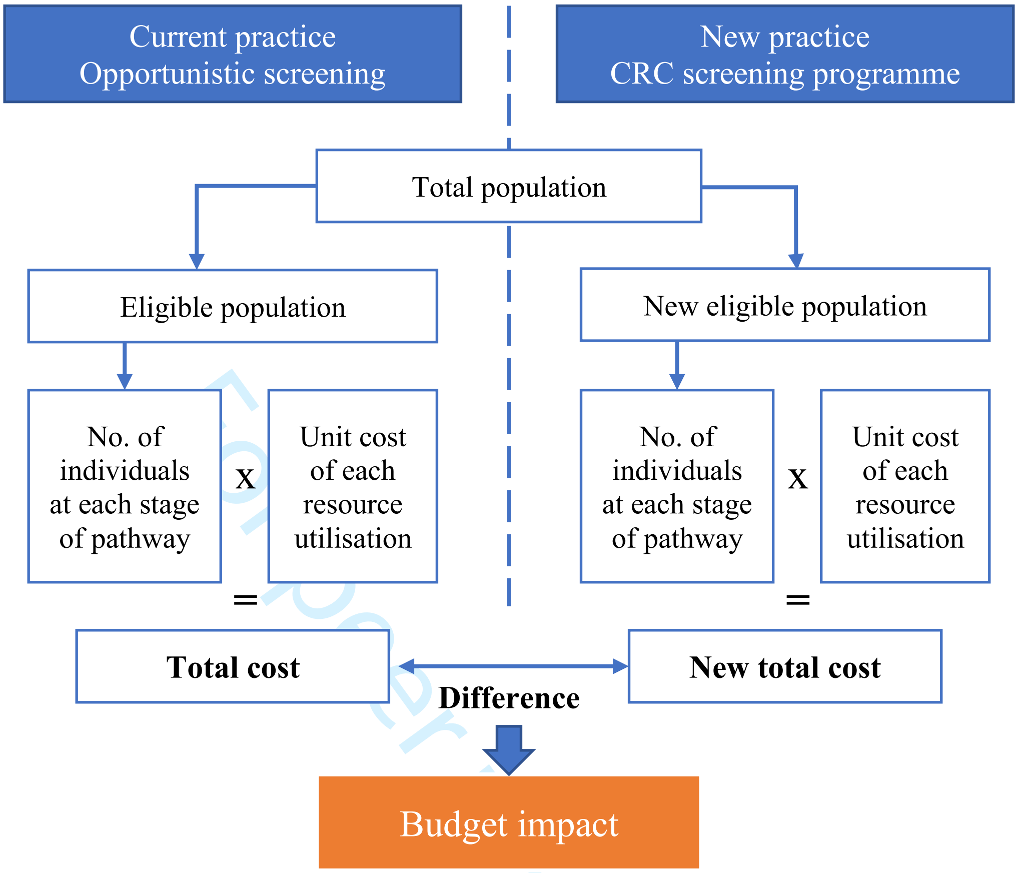

Supplementary material

Table S1: Net budget impact of CRC screening programme over 5-year timeframe, by state

Currency: Malaysian ringgit

State	Population	Year 1	Year 2	Year 3	Year 4	Year 5
**Johor**	3,822,800	12,597,041	13,791,897	14,986,752	16,181,607	17,376,462
**Kedah**	2,206,200	7,272,541	7,962,112	8,651,682	9,341,252	10,030,823
**Kelantan**	1,884,300	6,212,311	6,801,268	7,390,225	7,979,183	8,568,140
**Melaka**	934,700	3,084,637	3,376,787	3,668,937	3,961,087	4,253,238
**Negeri Sembilan**	1,141,000	3,764,122	4,120,753	4,477,384	4,834,016	5,190,647
**Pahang**	1,702,900	5,614,833	6,147,092	6,679,351	7,211,609	7,743,868
**Perak**	2,569,300	8,468,450	9,271,511	10,074,572	10,877,633	11,680,693
**Pulau Pinang**	1,767,100	5,826,301	6,378,626	6,930,951	7,483,276	8,035,601
**Sabah**	3,919,600	12,915,864	14,140,975	15,366,086	16,591,197	17,816,308
**Sarawak**	2,829,400	9,325,144	10,209,501	11,093,859	11,978,217	12,862,575
**Terengganu**	1,245,300	4,107,648	4,496,879	4,886,111	5,275,342	5,664,573
**Perlis**	253,500	841,028	920,262	999,496	1,078,730	1,157,964
**W.P. Kuala Lumpur**	1,790,100	5,902,043	6,461,557	7,021,071	7,580,585	8,140,099
**W.P. Labuan**	99,000	332,138	363,081	394,024	424,968	455,911
**W.P. Putrajaya**	97,100	325,886	356,236	386,585	416,935	447,284
**Nation-wide**	**32,676,786**	**107,631,959**	**117,845,422**	**128,058,885**	**138,272,349**	**148,485,812**

CRC: Colorectal cancer | W.P.: The Federal Territories (Malay: Wilayah Persekutuan)

Readers can convert from Malaysian Ringgit to their currency of interest (e.g., International Dollar, US Dollar, British Pound, Euro etc.) using the free web-based tool 'CCEMG – EPPI-Centre Cost Converter' (https://eppi.ioe.ac.uk/costconversion/default.aspx). This tool help adjusting estimates of cost expressed in one currency and price year to a specific target currency and price year.

Supplementary material

Table S2: Sensitivity of the total budget impact of CRC screening programme to changes in each variable individually

Baseline budget impact=RM107,631,959

Unit: Thousand Ringgit Malaysia

	Baseline value	Min value (-20% from baseline)	Max value (+20% from baseline)	Min budget impact	Max budget impact	Change
Probability of adults is contactable for CRC screening programme	66%	53%	79%	86,574	129,818	43,244
Probability of adults is included (eligible for CRC screening programme)	77%	62%	92%	86,386	128,950	42,564
Probability of adults agree to participate in CRC screening programme after being invited	52%	42%	63%	86,454	129,675	43,221
Probability of adults needing 1st reminder	79%	63%	94%	107,430	107,766	336
Probability of adults needing 2nd reminder	88%	70%	100%	107,535	107,643	108
Probability of adults return iFOBT result	42%	33%	50%	105,062	110,060	4,998
Probability of adults with iFOBT positive result	18%	14%	22%	105,204	109,988	4,784
Probability of adults taking colonoscopy after positive iFOBT	41%	33%	49%	105,673	109,493	3,820
Probability of adults with CRC detection after getting colonoscopy	4%	3%	5%	107,594	107,603	9
Cost to perform the screening (asking for symptoms, family history, referral)	5.58	4.47	6.70	107,633	107,567	-66
Cost of stool specimen processing	1.70	1.36	2.04	107,610	107,590	-20
Interpretation of results	2.79	2.23	3.35	107,617	107,583	-34
Cost to convey definitive diagnosis to patients (along with explaining treatment plan or referral etc.)	8.37	6.70	10.05	107,597	107,604	7
Contact eligible individuals - agreed to participate	0.98	0.79	1.18	107,285	107,926	641
Contact eligible individuals - rejected/excluded to participate	0.47	0.38	0.56	107,465	107,735	270
iFOBT rapid test kit (only the rapid test kit itself)	6.90	5.52	8.28	105,331	109,870	4,539
Print materials (instruction leaflet, explanatory statement)	1.10	0.88	1.32	107,238	107,962	724
Postage (stamp etc.)	5.35	4.28	6.42	105,840	109,360	3,520
Sending video through Whatapp	0.41	0.33	0.50	107,461	107,740	279
Sending reminder text message	0.41	0.33	0.50	107,490	107,710	220
Reminder call	0.28	0.22	0.33	107,536	107,661	125
Interpret the test kit result	1.70	1.36	2.04	107,366	107,832	466

For peer review only

Supplementary material

Unit: Thousand Ringgit Malaysia

	**Baseline value**	**Min value (-20% from baseline)**	**Max value (+20% from baseline)**	**Min budget impact**	**Max budget impact**	**Change**
Sending text message informing negative result	0.45	0.36	0.54	107,550	107,651	101
Call to inform positive result	0.41	0.33	0.50	107,590	107,611	21
Prepare and send referral letter	1.12	0.90	1.35	107,573	107,628	55
Follow up effort	6.73	5.39	8.08	107,503	107,698	195
Colonoscopy	200	160	240	105,638	109,564	3,926
Consumables – stool container, gloves, mask, plastic waste bag and disposal of materials from the test	10.80	8.64	12.96	93,612	121,641	28,029

For peer review only

ISPOR—The Professional Society for Health Economics and Outcomes Research

Budget Impact Analysis—Principles of Good Practice: Report of the ISPOR 2012 Budget Impact Analysis Good Practice II Task Force

Citation: Sullivan SD, Mauskopf JA, Augustovski F, Jaime Caro J, Lee KM, Minchin M, Orlewska E, Penna P, Rodriguez Barrios JM, Shau WY. Budget impact analysis-principles of good practice: report of the ISPOR 2012 Budget Impact Analysis Good Practice II Task Force. Value Health. 2014 Jan-Feb;17(1):5-14. doi: https://doi.org/10.1016/j.jval.2013.08.2291

Recommendations for Reporting Format

Section/topic	Guidance for reporting	Reported in section
**Introduction**		
Objectives	The objective of the BIA should be clearly stated and tied to the study perspectives	Introduction
Epidemiology and management of health problem	Present information about the prevalence and incidence of the particular disease, disease severity, disease progression, undiagnosed or undertreated cases, and risk factors pertinent to estimating the budget impact	Introduction
Clinical impact	Consist of a brief description of the eligible population and existing management options and their efficacy and safety that are relevant to the design of the study of the BIA	Introduction
Economic impact	Include a brief description of previous BIAs in the condition of interest for another intervention and condition-specific treatment patterns and cost of-care studies	Not applicable (No previous BIA)
**Study Design and Methods**		
Patient population	Specify the eligible population for the new intervention	Methods<br>Sub-section: Eligible population and input assumptions<br>Table 1
Intervention mix	Contain a detailed description of the use and characteristics of each intervention in the current intervention mix and in the expected intervention mix after the introduction of the new intervention	Methods<br>Sub-section: Health service under assessment and its comparator<br>Figure 1 and 2
Time horizon	Should be presented and the choice(s) justified	Methods<br>Sub-section: Perspective and time horizon
Perspective	Identify the BIAs' perspective(s), the cost categories included, and the intended audience	Methods<br>Perspective and time horizon

For peer review only - http://bmjopen.bmj.com/site/about/guidelines.xhtml

ISPOR—The Professional Society for Health Economics and Outcomes Research

Section/topic	Guidance for reporting	Reported in section
Analytic framework description	Complete description of the structure of the BIA cost calculator or condition-specific cohort or individual simulation model	Methods<br>Sub-section: Eligible population and input assumptions
Input data	Input values used for the reported analyses, including alternative scenarios, should be presented	Methods<br>Sub-section: Cost input and data sources<br>Table 2
Data sources	The sources of data inputs should be described in detail	Methods<br>Sub-section: Cost input and data sources<br>Table 2
Data collection	The methods and processes for any primary data collection and data abstraction tasks not reported elsewhere should be described and explained.	Not applicable (secondary data)
Analyses	A description of the calculations used to complete the BIA should be provided. The choice of all the scenarios presented in the results should be documented and justified.	Methods<br>Sub-section: Computing framework and base-case analysis under budget impact analyses
Uncertainty	Uncertainty analysis methods should be described and justified	Methods<br>Sub-sections: Uncertainty and scenario analyses under budget impact analyses
**Results**	The budget impact should be presented for each budget period over the time horizon. Both budget period resource use and costs should be presented. The estimates of resource use should be listed in a table that shows the change in use for each time period reported in the BIA<br><br>The results of the uncertainty analyses and scenarios analyzed should be described and presented in figures or tables	Results<br>Table 3<br><br>Results<br>Figure 2
**Conclusions and Limitations**	State the main conclusions on the basis of the results of the BIA.<br>Report the main limitations regarding key issues such as design aspects including off-label use and adherence assumptions and the completeness and quality of data inputs and sources.	Discussion<br><br>Conclusion
**Inclusion of Graphics and Tables**		
Figure of the analytical framework	Flow diagrams or other visual depictions of the cost calculator or condition-specific cohort or individual simulation model are recommended to be included with the analytical framework description.	Supplementary material<br>Figure S1

For peer review only - http://bmjopen.bmj.com/site/about/guidelines.xhtml

ISPOR—The Professional Society for Health Economics and Outcomes Research

Section/topic	Guidance for reporting	Reported in section
Table of assumptions	All the major assumptions should be listed in a tabular form	Table 1
Tables of inputs	All the input parameter values and their data sources and<br>derivations should be presented in a tabular form	Table 2
Tables of outputs	All outputs should be presented in a tabular and/or graphical<br>Form	Table 3
Schematic representation of uncertainty analyses	Diagrams such as Tornado diagrams should be included along with the text on the results of the scenario analyses	Figure 3
Appendices and References	The appendices may cover literature search strategies, evidence summaries, intermediate results (e.g., of individual Delphi panel rounds), and the names and addresses of participating experts and investigators, for example.	Reference

For peer review only - http://bmjopen.bmj.com/site/about/guidelines.xhtml

CHEERS 2022 Checklist

Topic	No.	Item	Location where item is reported
**Title**			
	1	Identify the study as an economic evaluation and specify the interventions being compared.	Title page, Page 1
**Abstract**			
	2	Provide a structured summary that highlights context, key methods, results, and alternative analyses.	Abstract, Page 1
**Introduction**			
**Background and objectives**	3	Give the context for the study, the study question, and its practical relevance for decision making in policy or practice.	Introduction, Line 65-69
**Methods**			
**Health economic analysis plan**	4	Indicate whether a health economic analysis plan was developed and where available.	Not applicable
**Study population**	5	Describe characteristics of the study population (such as age range, demographics, socioeconomic, or clinical characteristics).	Methods, Line 123-152, Table 1
**Setting and location**	6	Provide relevant contextual information that may influence findings.	Methods, Line 79-92
**Comparators**	7	Describe the interventions or strategies being compared and why chosen.	Methods, Line 79-121, Figure 1 and 2
**Perspective**	8	State the perspective(s) adopted by the study and why chosen.	Methods, Line 199-202
**Time horizon**	9	State the time horizon for the study and why appropriate.	Methods, Line 204-205
**Discount rate**	10	Report the discount rate(s) and reason chosen.	Methods, Line 205-206
**Selection of outcomes**	11	Describe what outcomes were used as the measure(s) of benefit(s) and harm(s).	Not applicable

Topic	No.	Item	Location where item is reported
**Measurement of outcomes**	12	Describe how outcomes used to capture benefit(s) and harm(s) were measured.	Not applicable
**Valuation of outcomes**	13	Describe the population and methods used to measure and value outcomes.	Not applicable
**Measurement and valuation of resources and costs**	14	Describe how costs were valued.	Methods, Line 159-196
**Currency, price date, and conversion**	15	Report the dates of the estimated resource quantities and unit costs, plus the currency and year of conversion.	Methods, Line 73-76
**Rationale and description of model**	16	If modelling is used, describe in detail and why used. Report if the model is publicly available and where it can be accessed.	Not applicable
**Analytics and assumptions**	17	Describe any methods for analysing or statistically transforming data, any extrapolation methods, and approaches for validating any model used.	Methods, Line 211-221, Figure S1 (Supplementary Material)
**Characterising heterogeneity**	18	Describe any methods used for estimating how the results of the study vary for subgroups.	Not applicable
**Characterising distributional effects**	19	Describe how impacts are distributed across different individuals or adjustments made to reflect priority populations.	Not applicable
**Characterising uncertainty**	20	Describe methods to characterise any sources of uncertainty in the analysis.	Methods, Line 224-229, Figure S2 (Supplementary Material)
**Approach to engagement with patients and others affected by the study**	21	Describe any approaches to engage patients or service recipients, the general public, communities, or stakeholders (such as clinicians or payers) in the design of the study.	Methods, Line 233-235
**Results**			
**Study parameters**	22	Report all analytic inputs (such as values, ranges, references) including uncertainty or distributional assumptions.	Table 1 and 2, Table S2 (Supplementary Material)
**Summary of main results**	23	Report the mean values for the main categories of costs and outcomes of interest and summarise them in the most appropriate overall measure.	Results, Line 239-274, Table 3

For peer review only - http://bmjopen.bmj.com/site/about/guidelines.xhtml

Topic	No.	Item	Location where item is reported
**Effect of uncertainty**	24	Describe how uncertainty about analytic judgments, inputs, or projections affect findings. Report the effect of choice of discount rate and time horizon, if applicable.	Results, Line 277-295, Figure 3
**Effect of engagement with patients and others affected by the study**	25	Report on any difference patient/service recipient, general public, community, or stakeholder involvement made to the approach or findings of the study	Not applicable
**Discussion**			
**Study findings, limitations, generalisability, and current knowledge**	26	Report key findings, limitations, ethical or equity considerations not captured, and how these could affect patients, policy, or practice.	Discussion
**Other relevant information**			
**Source of funding**	27	Describe how the study was funded and any role of the funder in the identification, design, conduct, and reporting of the analysis	End of manuscript
**Conflicts of interest**	28	Report authors conflicts of interest according to journal or International Committee of Medical Journal Editors requirements.	End of manuscript

From: Husereau D, Drummond M, Augustovski F, et al. Consolidated Health Economic Evaluation Reporting Standards 2022 (CHEERS 2022) Explanation and Elaboration: A Report of the ISPOR CHEERS II Good Practices Task Force. Value Health 2022;25. doi:10.1016/j.jval.2021.10.008

w only

For peer review only - http://bmjopen.bmj.com/site/about/guidelines.xhtml

A budget impact analysis of a home-based colorectal cancer screening programme in Malaysia

Journal:	*BMJ Open*
Manuscript ID	bmjopen-2022-066925.R3
Article Type:	Original research
Date Submitted by the Author:	09-Mar-2023
Complete List of Authors:	Ngan, Tran; Queen's University Belfast, Centre for Public Health<br>Ramanathan, Kogila; Monash University Malaysia, Global Public Health, Jeffrey Cheah School of Medicine and Health Sciences; Monash University Malaysia, South East Asia Community Observatory<br>Saleh, Muhamad; Monash University Malaysia, South East Asia Community Observatory<br>Schliemann, Desiree ; Queen's University Belfast, Centre for Public Health<br>Ibrahim Tamin, Nor Saleha ; Ministry of Health Malaysia<br>Su, Tin ; Monash University Malaysia, Global Public Health, Jeffrey Cheah School of Medicine and Health Sciences; Monash University Malaysia, South East Asia Community Observatory<br>Donnelly, Michael; Queen's University Belfast, Centre for Public Health<br>O'Neill, Ciaran; Queen's University Belfast, Centre for Public Health
**Primary Subject Heading**:	Health economics
Secondary Subject Heading:	Global health, Public health, Oncology
Keywords:	Health policy < HEALTH SERVICES ADMINISTRATION & MANAGEMENT, ONCOLOGY, HEALTH ECONOMICS, PUBLIC HEALTH

I, the Submitting Author has the right to grant and does grant on behalf of all authors of the Work (as defined in the below author licence), an exclusive licence and/or a non-exclusive licence for contributions from authors who are: i) UK Crown employees; ii) where BMJ has agreed a CC-BY licence shall apply, and/or iii) in accordance with the terms applicable for US Federal Government officers or employees acting as part of their official duties; on a worldwide, perpetual, irrevocable, royalty-free basis to BMJ Publishing Group Ltd ("BMJ") its licensees and where the relevant Journal is co-owned by BMJ to the co-owners of the Journal, to publish the Work in this journal and any other BMJ products and to exploit all rights, as set out in our licence.

The Submitting Author accepts and understands that any supply made under these terms is made by BMJ to the Submitting Author unless you are acting as an employee on behalf of your employer or a postgraduate student of an affiliated institution which is paying any applicable article publishing charge ("APC") for Open Access articles. Where the Submitting Author wishes to make the Work available on an Open Access basis (and intends to pay the relevant APC), the terms of reuse of such Open Access shall be governed by a Creative Commons licence – details of these licences and which Creative Commons licence will apply to this Work are set out in our licence referred to above.

Other than as permitted in any relevant BMJ Author's Self Archiving Policies, I confirm this Work has not been accepted for publication elsewhere, is not being considered for publication elsewhere and does not duplicate material already published. I confirm all authors consent to publication of this Work and authorise the granting of this licence.

For peer review only

A budget impact analysis of a home-based colorectal cancer screening programme in Malaysia

Tran Thu Ngan[1*], Kogila Ramanathan[2,3], Muhamad Raziq Bin Mohd Saleh[3], Désirée Schliemann[1], Nor Saleha Binti Ibrahim Tamin[4], Tin Tin Su[2,3], Michael Donnelly[1], Ciaran O'Neill[1]

[1] *Centre for Public Health, Queen's University Belfast, Belfast, UK*

[2] *Global Public Health, Jeffrey Cheah School of Medicine and Health Sciences, Monash University Malaysia, Selangor, Malaysia*

[3] *South East Asia Community Observatory (SEACO), Jeffrey Cheah School of Medicine and Health Sciences, Monash University Malaysia*

[4] *Ministry of Health Malaysia, Putrajaya, Malaysia*

* Correspondence to:

Tran Thu Ngan, PhD

Postal address: Centre for Public Health, Queen's University Belfast, Belfast, United Kingdom

Email: n.t.tran@qub.ac.uk

ORCID of the authors

Tran Thu Ngan : 0000-0003-2771-9878

Désirée Schliemann : 0000-0002-8746-3002

Ciaran O'Neill : 0000-0001-7668-3934

For peer review only

A budget impact analysis of a home-based colorectal cancer screening programme in Malaysia

Abstract

Objectives: The 2020-2022 research project '**C**olo**r**ectal **C**ancer **S**creening **I**ntervention for **M**alaysia' (CRC-SIM) evaluated the implementation of a home-based CRC screening pilot in Segamat District. This budget impact analysis (BIA) assessed the expected changes in health expenditure of the Malaysian Ministry of Health budget in the scenario where the pilot programme was implemented nationwide versus current opportunistic screening.

Design: Budget impact analysis. Assumptions and costs in the opportunistic and novel CRC screening scenarios were derived from a previous evaluation of opportunistic CRC screening in community health clinics across Malaysia and the CRC-SIM research project, respectively.

Setting: National level (with supplement analysis for district level). The BIA was conducted from the viewpoint of the federal government and estimated the annual financial impact over a period of five years.

Results: The total annual cost of the current practice of opportunistic screening was RM1,584,321 (~I$1,099,460; RM=Ringgit Malaysia; I$=International dollar) of which 80% (RM1,274,690 or ~I$884,587) was expended on the provision of opportunistic CRC to adults who availed of the service. Regarding the implementation of national CRC screening programme, the net budget impact in the 1st year was estimated to be RM107,631,959 (~I$74,692,546) and to reach RM148,485,812 (~I$103,043,589) in the 5th year based on an assumed increased uptake of 5% annually. The costs were calculated to be sensitive to the probability of adults who were contactable, eligible, and agreeable to participating in the programme.

Conclusions: Results from the BIA provided direct and explicit estimates of the budget changes to when implementing a population-based national CRC screening programme to aid decision making by health services planners and commissioners in Malaysia about whether such programme is affordable within given their budget constraint. The study also illustrates the use and value of the BIA approach in LMICs and resource-constrained settings.

Keywords: Colorectal cancer screening, budget impact analysis, home-based testing, global health, Malaysia

For peer review only - http://bmjopen.bmj.com/site/about/guidelines.xhtml

Strengths and limitations of this study

- The budget impact analysis (BIA) was used to evaluate the ‘affordability’ of colorectal cancer (CRC) screening programme in Malaysia within given budget constraint.
- Assumptions and cost inputs for modelling the budget impact were based on the actual costs and rates observed in Malaysia.
- The total cost of resources (=unit costs * number of users) for opportunistic screening and the CRC screening programme were compared to calculate the net budget impact.
- The BIA was conducted from the viewpoint of the federal government and only included costs and resource requirements relevant to this particular budget holder.
- The BIA could not and was not intended to provide answers to questions about whether or not the screening programme is good value for money (which can be answered by a cost-effectiveness analysis).

INTRODUCTION

Colorectal cancer (CRC) has the second highest incidence and mortality rate among all types of cancer in both sexes in Malaysia [1]. The age standardised incidence rate in 2012-2016 was 14.8 per 100,000 males and 11.1 per 100,000 females which appears to be stable compared to 2007-2011 [2]. In contrast, the proportion of CRC patients who are diagnosed at a late stage (i.e., stage III or IV) is increasing. Report from Ministry of Health Malaysia (MoHM) showed that the proportion of males with late stage CRC increased from 65.9% during 2007-2011 to 72.4% during 2012-2016; and from 65.2% to 73.1% for females [2]. The report did not give an explanation about this increasing trend though [2]. Late stage diagnosis negatively impacts survival rate; for example, the 5-year survival rates for cases diagnosed at stage I, II, III, and IV in 2002-2004 in Kuala Lumpur were 78.6%, 52.9%, 44.3%, and 9.3%, respectively [3]. Improved survival can be achieved by early detection through screening and the removal of premalignant polyps [4]. However, Malaysia currently does not have a population-based national CRC screening programme.

For peer review only

The Ministry of Health of Malaysia (MoHM) adopted the use of immunochemical faecal occult blood test (iFOBT) for opportunistic CRC screening at public health clinics since 2014 [5]. MoHM guidelines recommend screening for asymptomatic individuals aged 50-75 years old with average risk of CRC [6]. The uptake (number of patients screened/total eligible population) of this opportunistic screening tends to be very low. The annual average uptake during 2014-2018 was 0.5% while the 5-year cumulative uptake was 2.29% due to low awareness about CRC in general and CRC tests in particular, fear of the result, concern about the cost, and absence of a doctor's recommendation [5, 7]. Home-based iFOBT has been implemented in many high-income countries (HICs) to improve the accessibility and uptake of CRC screening [8]. In this context, the Southeast Asia Community Observatory (SEACO) at Monash University Malaysia and Queen's University Belfast (Northern Ireland) collaborated to conduct the research project, '**C**olo**r**ectal **C**ancer **S**creening **I**ntervention for **M**alaysia' (CRC-SIM) in 2020-2022. This project evaluated the implementation of a home-based CRC screening pilot in Segamat District. The uptake of the novel screening programme was 22%. The significantly higher uptake indicates the potential population wide impact if this screening approach (i.e., using home-based iFOBT and self-reporting test results) was scaled up. However, in order to aid public health decision making, there is a need to model a scaled-up version of the research-tested screening programme and, more specifically, gather insights about the total costs of programme implementation and how it might impact the MoHM budget. In other words, there is a need for a budget impact analysis (BIA).

Budget impact analysis was first introduced in 1998 by Mauskopf [9, 10]. Since then, BIA is gradually requested as a part of the health technology assessment (HTA) procedure by a few countries around the world such as Australia, Canada, the United States (the US), England, Ireland, Spain, Belgium, Poland, Israel, and Thailand [11]. Regarding BIA for colorectal cancer

For peer review only - http://bmjopen.bmj.com/site/about/guidelines.xhtml

(CRC) screening, a recent systematic review found six studies conducted in the UK, US, Belgium, and Australia [12]. We found two additional studies published in 2018 and 2019 from Spain and Thailand, respectively [13, 14]. Although results from these studies are not comparable as they were specific to each studied country, all studies were conducted to answer the question 'What is the budget impact of implementing a colorectal cancer screening/prevention programme compared with current usual care'. It is also the research question that the BIA in this study aims to answer. Specifically, the BIA assessed the expected changes in the health expenditure of MoHM budget as a result of implementing a population-based national CRC screening programme versus current opportunistic screening (or 'usual care'). It assessed the affordability of the screening programme given potential budget constraints.

METHODS

The conduct of this BIA and presentation of this paper followed the guidelines developed by the International Society for Pharmacoeconomics and Outcomes Research (ISPOR) Task Force [11, 15]. All costs are presented in local currency -the Malaysian Ringgit (RM)- and International Dollar (I$). RM was converted to I$ using purchasing power parity (PPP) conversion factors instead of market exchange rates. The PPP conversion rate of 1.441 was obtained from the IMF World Economic Outlook Database [16].

Health service under assessment and its comparator

The specific health service that was the focus of the BIA was a population-based screening programme for colorectal cancer using a self-rapid response iFOBT. The comparator was current or 'usual care' - opportunistic screening.

For peer review only - http://bmjopen.bmj.com/site/about/guidelines.xhtml

The BIA is predicated on the opportunistic screening programme being replaced by the new population-based screening programme (i.e., the two programmes would not be run in conjunction or in other words, the two scenarios in assessment are mutually exclusive). In each scenario, the patient pathway from the point when patients were invited for screening to receipt of a definitive diagnosis were identified and described. The screening procedure ends at the point of a patient receiving their iFOBT result with encouragement to attend hospital for a colonoscopy (if iFOBT is positive). It is important to note that the BIA included costs of screening and diagnosis (e.g., colonoscopy, biopsy) but not treatment. The BIA also did not address issues with respect to equity of access and uptake of services in either screening scenarios.

The patient pathways for the 'usual care' practice and the novel CRC screening programme are presented in Figure 1 and 2, respectively. In opportunistic screening practice, it is recommended or expected that individuals who are aged 50-75 years will be screened for CRC symptoms when they attend their local health clinic (for any health condition or problem). If they are asymptomatic and have an average risk of having CRC (based on family history), they are offered an iFOBT, followed by a colonoscopy if the iFOBT test was positive. If CRC is detected following a colonoscopy, the result is conveyed to a patient along with an explanation of the treatment plan or referral arrangement.

(Figure 1 is about here)

Details of the home-based screening intervention in CRC-SIM were published elsewhere [17]. Briefly, in the novel CRC screening programme, individuals aged 50-75 years were contacted, checked for eligibility, and invited to participate. A home-screening 'pack' was posted to eligible participants followed by two reminders. The test was performed at home by participants who took a photograph of the completed test and texted it to trained medical professionals who interpreted the photograph. Participants with positive iFOBT were referred for a colonoscopy at hospital.

For peer review only - http://bmjopen.bmj.com/site/about/guidelines.xhtml

(Figure 2 is about here)

There were two main differences between these patient pathways. Firstly, individuals within the target age group for screening were contacted directly and invited to participate in the novel CRC screening programme while in the situation of 'usual care', CRC screening was offered (if screening guideline recommendations were followed) only when members of the target group visited their clinic for some other health condition or problem. Secondly, the iFOBT was performed by doctors at health clinics in the 'usual care' pathway while in the novel CRC screening programme, participants self-tested in their home. Home-based testing generated additional stages in the pathways in relation to sending a test, reminding participants, taking a photo of a completed test, and sending it to programme officers and vice versa. The remaining stages of each pathway (e.g., being screened for eligibility, receiving a colonoscopy, and receiving a treatment plan) were the same across the two scenarios.

Eligible population and input assumptions

The target population for current opportunistic screening in Malaysia is individuals aged 50-75 years, regardless of sex. Due to the nature of home-based screening, the target population for the CRC screening programme was required to meet some additional inclusion criteria as presented in Figure 2. The number of individuals who presented and completed each stage was estimated using input assumptions.

Data about the population of Malaysia by age was taken from government reports (i.e., Department of Statistics, Malaysia) and from World Population Review [18, 19]. The total population was reported to be 32,676,786 in 2021, of which, 19% or 6,228,195 were aged 50-75 years old [18, 19].

For peer review only - http://bmjopen.bmj.com/site/about/guidelines.xhtml

In the 'usual care' – opportunistic screening pathway or scenario, all assumptions were derived from a study by Tamin NSI (2020) which was a 5-year evaluation of opportunistic CRC screening (and the use of stool-based tests) in community health clinics across Malaysia [5]. It was assumed that 0.482% of the eligible population would avail of CRC screening when they attended local health clinics for other conditions; and 9.21% of this proportion of tested patients would receive a positive result. Only 55.9% of patients in the study by Tamin availed of a colonoscopy after a positive iFOBT. CRC detection after colonoscopy investigation was 4.04%.

In the novel CRC screening programme, all assumptions were derived from the CRC-SIM research project. It was assumed that 50.51% of the eligible population would be contactable and meet all inclusion criteria to participate in the home-based screening programme; 52.27% of people who were eligible would agree to participate; 41.63% would perform the iFOBT and send a photo of a completed test to the programme officers; 18.01% of people who would be tested would receive a positive result; 41.07% would avail of colonoscopy after a positive iFOBT result; and CRC detection after colonoscopy investigation would be 4.35%.

Table 1 summarises details about the input assumptions that were used to estimate the number of individuals at each stage of the respective pathway: the opportunistic screening pathway and the CRC screening programme pathway.

For peer review only - http://bmjopen.bmj.com/site/about/guidelines.xhtml

Table 1: Input assumptions used to estimate the population at each stage of the patient pathways

Stage in pathway	Opportunistic screening scenario (Current practice)		Population-based CRC programme screening scenario (Proposed practice)	
	Assumption*	No. of individuals	Assumption**	No. of individuals
Total population (all ages)	NA	32,676,786	NA	32,676,786
Target population (aged 50-75)	19.06%	6,228,195	19.06%	6,228,195
Eligible population (met all inclusion criteria)	100%	6,228,195	50.51%	3,146,020
Availed of/agreed to take CRC screening	0.482%	30,020	52.27%	1,644,561
Needed 1st reminder to return the iFOBT result (among those agreed to participate)	NA	NA	78.71%	1,294,514
Needed 2nd reminder to return the iFOBT result (among those received 1st reminder)	NA	NA	88.10%	1,140,405
Returned iFOBT result (among those agreed to participate)	100%	30,020	41.63%	684,683
Received iFOBT positive result	9.21%	2,765	18.01%	123,287
Availed of colonoscopy after positive iFOBT	55.9%	1,546	41.07%	50,636
CRC detection after colonoscopy investigation	4.04%	62	4.35%	2,202

CRC: Colorectal cancer; iFOBT: Immunochemical faecal occult blood test; NA: Not applicable; No: Number

** The assumptions were derived from a study of Tamin NSI (2020) which was a 5-year evaluation of using stool-based test for opportunistic CRC screening in primary health institutions across Malaysia [5].*

*** The assumptions were derived from the Colorectal Cancer Screening Intervention for Malaysia (or CRC-SIM research project) in Segamat District, conducted by Queen's University Belfast, Monash University, and Southeast Asia Community Observatory (SEACO) in 2021.*

For peer review only

Cost input and data sources

In the opportunistic screening scenario, the total cost comprised the cost of:

(i) performing screening (e.g., asking for symptoms, family history, and collecting the sample)

(ii) processing stool specimens

(iii) interpreting test results and

(iv) conveying a definitive diagnosis to patients (include explaining treatment plan or referral arrangements)

In the CRC programme screening scenario, the total cost comprised the costs of:

(i) contacting potential participants

(ii) delivering iFOBT test kits (including cost of the test, postage, print materials, and sending video instruction)

(iii) sending a reminder to participants (up to 2 times, by text message and phone call)

(iv) interpreting and conveying results to participants and

(v) following-up patients with positive iFOBT but did not take colonoscopy in order to encourage them to avail of the colonoscopy

These costs were calculated by multiplying the time allocated for the completion of each task with the salary cost of the person who undertakes each task plus cost of consumables. Table 2 shows the unit cost for each cost element, related assumptions, and data sources.

For peer review only

For peer review only - http://bmjopen.bmj.com/site/about/guidelines.xhtml

Table 2: Resources and unit costs

Currency: Malaysian ringgit (RM)

Cost element	Unit cost *(Per screen) RM (I$)*	Assumptions	Source
Current practice (opportunistic screening)			
Performing screening (asking for symptoms, family history, referral) and taking sample	5.58	20 min x salary RM2947/month	1
Processing stool specimens	1.70	10 min x salary RM1797/month	2
Interpreting the test results	2.79	10 min x salary RM2947/month	1
Conveying a definitive diagnosis to patients (along with explaining treatment plan or referral etc.)	8.37	30 min x salary RM2947/month	1
Proposed practice (Population-based CRC screening programme)			
Contact eligible individuals - agreed to participate	0.98	7.1 min x (salary RM1440/month + mobile package RM20/month)	3
Contact eligible individuals - rejected/excluded to participate	0.47	3.4 min x (same as above)	3
iFOBT rapid test kit	6.90		3
Print materials (instruction leaflet, explanatory statement)	1.10	90 cents for colour print + 20 cent for black & white print	3
Postage (stamps, etc.)	5.35		3
Sending video through WhatsApp	0.41	3 min x (salary RM1440/month + mobile package RM20/month)	3
Sending reminder text message	0.41	3 min x (same as above)	3
Reminder call	0.28	2 min x (same as above)	3
Interpreting the test kit result	1.70	10 min x salary RM1797/month	3
Sending text message to inform patient of negative result	0.45	2 min x (salary RM2350/month + mobile package RM20/month)	3
Calling patient to inform him/her of positive result	0.67	3 min x (same as above)	3
Preparing and sending referral letter to patient/clinic	1.12	5 min x (same as above)	3
Follow up effort	6.73	30 min x (same as above)	3
Developing communication materials, **one-off cost**	6,063	Communication materials do not change in 5 years	3

Currency: Malaysian ringgit (RM)

Cost element	Unit cost *(Per screen) RM (I$)*	Assumptions	Source
Training for data collectors*, **one-off cost**<br>** Data collectors are those employed by the programme to (i) contact potential participants, (ii) deliver iFOBT test kits, and (iii) send a reminder to participants*	109,703	+ 1 day training (virtual using Zoom)<br>+ 1 trainer for maximum 25 trainees<br>+ 1 data collector* is needed for every target population of 400<br>+ Cost=1-day-salary of trainer/trainees x number of trainer/trainees<br>+ No retraining in 5 years	3
Same in both scenarios/practices			
Colonoscopy (including polyps removal and/or biopsy if needed)	200		
Consumables – stool container, gloves, mask, plastic waste bag and disposal of materials from the test	10.80	RM8636.7/800 sets	3

iFOBT: Immunochemical faecal occult blood test; RM: Malaysian ringgit

Source:

1. *Public Services Commission of Malaysia. Medical Officer Grade UD41. Accessed at https://www.spa.gov.my/spa/laman-utama/gaji-syarat-lantikan-deskripsi-tugas/ijazah-sarjana-phd/pegawai-perubatan-gred-ud41*
2. *Public Services Commission of Malaysia. Medical laboratory technologist Grade U29. Accessed at https://www.interactive.jpa.gov.my/ezskim/klasifikasi/perbekalanskim.asp?id_skim=3LU03*
3. *Colorectal Cancer Screening Intervention for Malaysia (or CRC-SIM research project) in Segamat District, conducted by Queen's University Belfast, Monash University, and Southeast Asia Community Observatory (SEACO) in 2021*

In the current practice of opportunistic screening, doctors were consulted about the estimated time to perform each stage in the pathway. The monthly salary of a general doctor and a medical laboratory technologist was based on the rate published by the Public Services Commission of Malaysia [20, 21]. These rates were RM2,947 (~I$2,045) and RM1,797 (~I$1,247), respectively. In the novel CRC screening programme, the time to perform each stage in the pathway, salary of personnel, and costs of material resources (e.g., rapid kit test, consumables, postage, printing materials) were based on the time and expenditure observed in the CRC-SIM research project. All costs were calculated per screen except the cost of training and the cost of developing communication materials which were one-off costs based on the assumption that communication materials would not change, and no re-training would be needed within 5 years.

For peer review only

For peer review only - http://bmjopen.bmj.com/site/about/guidelines.xhtml

It was assumed (based on the experience of operating the screening programme during the CRC-SIM project) that one data collector (i.e., those employed by the programme to (i) contact potential participants, (ii) deliver iFOBT test kits, and (iii) send a reminder to participants) would be needed for every 400 people in the target population. Training would last one day and would be delivered virtually; thus, the cost of training equalled (1-day-salary of trainer x number of trainer) + (1-day-salary of trainees x number of trainees/data collectors).

Perspective and time horizon

The BIA was conducted from the viewpoint of the federal government which finances Malaysia's public health system [22]. Only those costs and resource requirements relevant to the budget holder were included in the analysis. For example, the out-of-pocket expenditure incurred by patients were excluded.

The analysis estimated the annual financial impact over a period of five years as recommended in the guidelines [11, 23]. Costs were not discounted given that the BIA methodology reports the costs for each year in which they occur rather than a net present value [11].

Budget impact analyses

Computing framework and base-case analysis

The BIA used a cost calculator programmed in Microsoft Excel, following the costing template[1] produced by the National Institute for Health and Care Excellence in the UK (NICE). The template was modified to fit the programme under assessment. The cost calculator

[1] The template can be freely downloaded at https://www.nice.org.uk/Media/Default/About/what-we-do/our-programmes/evidence-standards-framework/budget-impact-template.xlsx

For peer review only - http://bmjopen.bmj.com/site/about/guidelines.xhtml

approach is recommended by guidelines as it is easy for stakeholders to understand and replicate the results [11].

First, the number of individuals who completed each stage was estimated (Table 1). The resources that were used at each stage of the respective pathways (in opportunistic screening and the novel CRC screening programme) were listed along with their unit costs (i.e., cost of each resource per person) (Table 2). Unit costs were multiplied by number of users to give the total cost of resources for each scenario. The net budget impact was calculated as the difference in cost between opportunistic screening and the CRC screening programme. Visual depiction of the cost calculator is shown in Supplementary Material, Figure S1.

Uncertainty and scenario analyses

The input assumptions (that were used to estimate the number of individuals at each stage of the respective pathway) and the cost inputs were varied, and then the impact of these changes in relation to the results was analysed to investigate the sensitivity of the budget impact results to variations in individual input. As recommended by Gray et al. (2011), the range of variation regarding parameters for which data sources about dispersion were unavailable were ±20% of the base case [24].

Patient and public involvement

It was not appropriate or possible to involve patients or the public in the design, or conduct, or reporting, or dissemination plans of our research as this type of study is a secondary analysis of data from a payer perspective (Ministry of Health Malaysia).

For peer review only - http://bmjopen.bmj.com/site/about/guidelines.xhtml

RESULTS

Base-case analysis

The total annual cost of the current practice of opportunistic screening is RM1,584,321 (~I$1,099,460), of which 80% (RM1,274,690 ~ I$884,587) was for providing opportunistic CRC to adults who availed of the service. Costs of providing colonoscopy (including polyps removal and/or biopsy if needed) after receipt of a positive iFOBT and conveying definitive diagnosis to patients (along with explaining treatment plan or referral etc.) after the outcome of the colonoscopy were RM309,108 (~I$214,509) and RM523 (~I$363), respectively.

The total annual cost over a 5-year period of the proposed practice (i.e., CRC screening programme) is shown in Table 3. It was assumed that the number of people who would agree to participate in the programme would increase by 5% each year (in consideration of health promotion activities as well as information flows including word of mouth between participants). Therefore, the financial impact would also increase accordingly.

For peer review only

Table 3: Annual cost of proposed practice (i.e., CRC screening programme)

Currency: Malaysian ringgit (RM) and International Dollar (I$)

Proposed practice	Year 1 RM *(I$)*	Year 2 RM *(I$)*	Year 3 RM *(I$)*	Year 4 RM *(I$)*	Year 5 RM *(I$)*
Contacting adults who are eligible for CRC screening programme (i.e., aged 50-75) and screen for eligibility of participating	2,320,148 *(1,610,096)*	2,320,148 *(1,610,096)*	2,320,148 *(1,610,096)*	2,320,148 *(1,610,096)*	2,320,148 *(1,610,096)*
Providing iFOBT test to adults who agreed to participate in CRC screening programme after being invited	93,654,886 *(64,992,981)*	102,612,907 *(71,209,512)*	111,570,928 *(77,426,043)*	120,528,949 *(83,642,574)*	129,486,970 *(89,859,105)*
Providing 1st reminder to participants	536,929 *(372,609)*	588,286 *(408,248)*	639,643 *(443,888)*	690,999 *(479,527)*	742,356 *(515,167)*
Providing 2nd reminder to participants	315,339 *(218,833)*	345,501 *(239,765)*	375,663 *(260,696)*	405,825 *(281,627)*	435,987 *(302,559)*
Interpreting returned iFOBT samples	1,165,129 *(808,556)*	1,276,572 *(885,893)*	1,388,016 *(963,231)*	1,499,460 *(1,040,569)*	1,610,903 *(1,117,906)*
Conveying result through message to participants with iFOBT negative result	251,990 *(174,872)*	276,093 *(191,598)*	300,196 *(208,324)*	324,298 *(225,050)*	348,401 *(241,777)*
Preparing and sending referral letter and calling participants with iFOBT POSITIVE result	221,356 *(153,613)*	242,529 *(168,306)*	263,701 *(182,999)*	284,874 *(197,692)*	306,046 *(212,384)*
Following up participants who DID NOT take colonoscopy after positive iFOBT	489,158 *(339,457)*	535,945 *(371,926)*	582,733 *(404,394)*	629,520 *(436,863)*	676,308 *(469,332)*
Providing colonoscopy (including polyps removal and/or biopsy if needed) to participants with positive iFOBT	10,127,147 *(7,027,861)*	11,095,801 *(7,700,070)*	12,064,455 *(8,372,280)*	13,033,109 *(9,044,489)*	14,001,764 *(9,716,700)*
Conveying definitive diagnosis to patients (along with explaining treatment plan or referral etc.) after the colonoscopy	18,432 *(12,791)*	20,195 *(14,015)*	21,958 *(15,238)*	23,721 *(16,461)*	25,484 *(17,685)*
Capital costs (Developing communication materials + Training for data collectors)	115,766 *(80,337)*	115,766 *(80,337)*	115,766 *(80,337)*	115,766 *(80,337)*	115,766 *(80,337)*
**Total cost of proposed practice**	**109,216,279** *(75,792,005)*	**119,429,743** *(82,879,766)*	**129,643,206** *(89,967,527)*	**139,856,670** *(97,055,288)*	**150,070,133** *(104,143,049)*

CRC: Colorectal cancer; iFOBT: Immunochemical faecal occult blood test

For peer review only

Similar to opportunistic screening, the cost to provide iFOBT to the eligible population who availed of the service accounted for 86% of the total cost of the proposed CRC screening programme. The second most costly component was the provision of colonoscopy (including polyps removal and/or biopsy if needed) to patients with an iFOBT positive result, at 9% of the total cost. The remaining nine cost components such as contacting potential participants, reminding participants to send photograph of iFOBT result, conveying diagnosis to participants and the follow-up effort added only up to 5% of the total cost.

The net budget impact in the 1st year of implementing CRC screening programme would be RM107,631,959 (~I$74,692,546 which equalled the total cost of future practice minus the total cost of current practice). The impact increases each year as the number of people who agree to participate in the programme increase, reaching RM117,845,422 (~I$81,780,307) in year 2, RM128,058,885 (~I$88,868,067) in year 3, RM138,272,349 (~I$ 95,955,829) in year 4, and RM148,485,812 (~I$103,043,589) in year 5.

The net budget impact of providing and delivering the CRC screening programme over the 5-year timeframe for each state in Malaysia (calculated according to the population size of each state) can be accessed in Supplementary Material, Table S1. These estimates aid service planning decisions if the novel pilot programme is implemented in one or more of these states before being scaled up into nationwide programme.

Uncertainty and scenario analyses

The tornado diagram in Figure 3 shows the change to net budget impact when assumptions and cost inputs were varied. It presents the results of multiple univariate sensitive analyses on key

For peer review only - http://bmjopen.bmj.com/site/about/guidelines.xhtml

inputs that exert the most influence on the net budget impact (See Table S2, Supplementary Material for results of multiple univariate sensitive analysis on all inputs). These inputs include the probability of (i) making successful contact with adults about the CRC screening programme, (ii) adults agreeing to participate, (iii) adults being eligible to participate in the programme, and (iv) the cost of consumables that are required to take a stool sample. The first three inputs influence the number of individuals who are present at each stage of the patient pathway.

(Figure 3 is about here)

The net budget impact would increase from RM107 million to RM130 million (~I$74-90 million) if there was a 20% increase in (i) the probability of adults who were contactable (from a contact list of people aged 50-75 years old) or (ii) the probability of adults agreeing to participate in the CRC screening programme or (iii) the probability of adults being eligible for the programme (i.e., aged 50-75 years old; having no symptoms of CRC, a smartphone, and WhatsApp; resident within programme area; and did not have colonoscopy this year). In other words, a 20% increase in each one of these factors would require an additional RM23 million (~I$16 million) to be budgeted for the programme. Likewise, a 20% increase in the cost of the consumables that are required for taking stool samples would mean that the programme would cost RM15 million (~I$10 million) more than the originally calculated total cost.

DICUSSION

The result of this analysis provides information to guide public health service planners and commissioners in their decisions about an alternative CRC screening strategy i.e., a population-based CRC screening programme using home-based iFOBT compared to current opportunistic screening. It concluded that the net budget impact in the 1st year of implementing a CRC screening programme of this kind would be RM107,631,959 (~I$74,692,546). The impact would increase by year due to increase in uptake and would reach RM148,485,812

For peer review only - http://bmjopen.bmj.com/site/about/guidelines.xhtml

319 (~I$103,043,589) in the 5th year of implementation. This analytical approach and the results of this analysis are presented as aids to better decision making by MoHs and stakeholders in lower-middle-income countries (LMICs) about health programme planning and in this particular illustrative case to the MoHM regarding the degree to which the proposed CRC screening programme is affordable.

The total budget that was allocated to the MoHM in 2022 was RM32.4 billion (~I$22.5 million) [25]. Spending on prevention and public health services in 2009 was reported to be RM1.6 billion (~I$1.1 million) [22]. More recent data and information about the size of the budget that is allocated to cancer screening is not available. As such, it is estimated that the net budget impact of implementing a CRC screening programme would account for between 7-10% of the total budget for prevention and public health services. This represents a significant proportion of the overall budget allocated for prevention programmes/interventions.

The key factor in the implementation of a population-based screening programme/service or the factor that has biggest impact on the budget is the size of the population who use the service. The degree of accuracy regarding population size estimates is related closely to the cost estimates in the budget. It is important for service planners to keep this point in mind and to take into account an increase in uptake and the impact of such an increase. Therefore, in the case of the CRC programme presented here, we assumed a 5% increase annually in uptake and calculated the net budget impact. The net budget impact can be recalculated according to the actual change in uptake after the programme is implemented.

Budget impact analysis is an economic assessment that is used to estimate the changes in expenditure of a specific budget holder if a new health technology/programme is implemented

For peer review only - http://bmjopen.bmj.com/site/about/guidelines.xhtml

[11]. As such, BIA complements other health economic evaluation methods such as cost-effectiveness analysis (CEA) to provide a comprehensive economic assessment of a health care intervention to decision makers [11]. A BIA aids decision making by health service planners and commissioners about whether an intervention or programme is affordable within given budget constraints while a CEA informs decisions about whether an intervention is good value for money [11, 26]. BIA and its pragmatic approach is an ideal method when a situation calls for an evaluation of 'affordability' which is of central importance in LMICs and, arguably, is the key concern of whoever is in charge of managing a health care budget [27, 28].

It could well be that savings in earlier treatment would counterbalance the additional budget impact. Likewise, reduction in travel and time costs of participant while using home-based screening would reduce the total costs of the screening programme from a societal perspective. If we assume that travel distance to a clinic is 10km (77% of Malaysian live within 5km of a clinic [29]), travel time is 10 minutes (travel speed = 60km/hour), opportunistic screening takes 40 minutes (Table 2), and performing iFOBT at home take 10 minutes, the reduction in travel and time costs will be 40 minutes. This can be monetised using Gross Domestic Product per capita at RM50,224 [30] to which is then added 10km x RM1 per km (i.e., tolls & fuel [31]) = RM14 per participant. Consistent with BIA best practice guidance these have not been included in our estimate of the BIA which focuses on costs to the provider. Further work in this area may though be useful or a health technology assessment given the potential for aspects of societal cost to influence cost-effectiveness and service uptake.

Finally, the conduct of BIA in this paper has some limitations. First, assumptions and cost inputs for the CRC screening programme were based on the costs and rates that were observed in the CRC-SIM research project. Due to unavailability of data about dispersion of the

For peer review only

For peer review only - http://bmjopen.bmj.com/site/about/guidelines.xhtml

parameters, the used range of variation (±20% of the base case) may overestimate the uncertainty and suggests that the next step for further research is a CEA where parameter uncertainty is investigated with actual data. The project was conducted in only one district (Segamat); and the distribution of three main ethnic groups (i.e., Malay, Chinese, Indian) in the project differed from the proportions that have been reported nation-wide (72%:24%:3% vs 62%:21%:6%, respectively). Therefore, it is important to be mindful of the possibility that the assumptions and inputs (based on the project) may not be representative for, or read across to, the whole population of Malaysia. Likewise, it is important to bear in mind that our findings do not include the perspective of other payers and may not generalise to other settings. The results are related directly to the context of the Malaysian health system and the epidemiology of CRC in the country though they are illustrative of the positive contribution of the BIA methodology and approach.

CONCLUSIONS

This study employed a BIA methodology to analyse the costs of a novel CRC screening programme using home-based iFOBT and mHealth versus the current opportunistic screening. The findings estimated the net budget impact of implementing a population-based national CRC screening programme in Malaysia. The modelling estimations are important considerations for health authorities when they are required to decide the affordability of implementing a programme and to aid budgetary planning as well as decision making, generally, about implementation. Our study illustrates the use and value of the BIA approach in LMICs and resource-constrained settings.

For peer review only

Abbreviations

BIA	Budget impact analysis
CEA	Cost-effectiveness analysis
CRC	Colorectal cancer
CRC-SIM	Colorectal Cancer Screening Intervention for Malaysia
iFOBT	Immunochemical faecal occult blood test
I$	International Dollar
ISPOR	The Professional Society for Health Economics and Outcomes Research
MoHM	Ministry of Health of Malaysia
NICE	National Institute for Health and Care Excellence
RM	Malaysian ringgit
SEACO	Southeast Asia Community Observatory
UK	The United Kingdom

For peer review only

DECLARATIONS

Research ethics approval. Not applicable (This study does not involve human participants).

Funding. This research was supported by the Medical Research Council (UK) Global Challenges Research Fund (MR/S014349/1) and the National Medical Research Register Malaysia (NMRR ID-21-02045-O7G). The funder of the study had no role in study design, data collection, data analysis, data interpretation, or writing of the report.

Competing interests. The authors have no conflicts of interest to declare that are relevant to the content of this article.

Availability of data and material. All data generated or analysed during this study are included in this published article.

For peer review only - http://bmjopen.bmj.com/site/about/guidelines.xhtml

Authors' contributions. TTN: Methodology, Formal Analysis, Writing – Original draft, Writing – Review & Editing. KR, MRS, and DS: Data Curation, Investigation, Writing – Review & Editing. ST: Resources, Writing – Review & Editing. TTS, and MD: Conceptualization, Funding acquisition, Writing – Review & Editing, Supervision. CON: Methodology, Formal Analysis, Writing – Review & Editing, Supervision.

REFERENCE

1. Global Cancer Observatory: Cancer Today [Internet]. International Agency for Research on Cancer. 2020 [cited 12 April 2021]. Available from: http://gco.iarc.fr/today.
2. Malaysian National Cancer Registry Report 2012-2016 [Internet]. Ministry of Health Malaysia. 2020 [cited April 12, 2022].
3. Veettil SK, Lim KG, Chaiyakunapruk N, Ching SM, Abu Hassan MR. Colorectal cancer in Malaysia: Its burden and implications for a multiethnic country. Asian Journal of Surgery. 2017;40(6):481-9.
4. Rawla P, Sunkara T, Barsouk A. Epidemiology of colorectal cancer: incidence, mortality, survival, and risk factors. Prz Gastroenterol. 2019;14(2):89-103.
5. Tamin NSI, Razalli KA, Sallahuddin SN, Chan HK, Hassan MRA. A 5-year evaluation of using stool-based test for opportunistic colorectal cancer screening in primary health institutions across Malaysia. Cancer epidemiology. 2020;69:101829.
6. Ministry of Health Malaysia. Clinical Practice Guidelines – Management of Colorectal Carcinoma. Putrajaya: Malaysia Health Technology Assessment Section (MaHTAS); 2017.
7. Schliemann D, Ramanathan K, Ibrahim Tamin NSB, Neill C, Cardwell CR, Ismail R, et al. Implementation of a colorectal cancer screening intervention in Malaysia (CRC-SIM) in the context of a pandemic: study protocol. BMJ Open. 2022;12(9):e058420.
8. Issa IA, Noureddine M. Colorectal cancer screening: An updated review of the available options. World journal of gastroenterology. 2017;23(28):5086-96.
9. Mauskopf J. Prevalence-Based Economic Evaluation. Value in Health. 1998;1(4):251-9.
10. Trueman P, Drummond M, Hutton J. Developing guidance for budget impact analysis. Pharmacoeconomics. 2001;19(6):609-21.
11. Sullivan SD, Mauskopf JA, Augustovski F, Jaime Caro J, Lee KM, Minchin M, et al. Budget impact analysis-principles of good practice: report of the ISPOR 2012 Budget Impact Analysis Good Practice II Task Force. Value Health. 2014;17(1):5-14.
12. Jahn B, Todorovic J, Bundo M, Sroczynski G, Conrads-Frank A, Rochau U, et al. Budget Impact Analysis of Cancer Screening: A Methodological Review. Appl Health Econ Health Policy. 2019;17(4):493-511.
13. Arrospide A, Idigoras I, Mar J, de Koning H, van der Meulen M, Soto-Gordoa M, et al. Cost-effectiveness and budget impact analyses of a colorectal cancer screening programme in a high adenoma prevalence scenario using MISCAN-Colon microsimulation model. BMC Cancer. 2018;18(1):464.
14. Phisalprapa P, Supakankunti S, Chaiyakunapruk N. Cost-effectiveness and budget impact analyses of colorectal cancer screenings in a low- and middle-income country: example from Thailand. Journal of medical economics. 2019;22(12):1351-61.

For peer review only - http://bmjopen.bmj.com/site/about/guidelines.xhtml

15. Mauskopf JA, Sullivan SD, Annemans L, Caro J, Mullins CD, Nuijten M, et al. Principles of good practice for budget impact analysis: report of the ISPOR Task Force on good research practices--budget impact analysis. Value Health. 2007;10(5):336-47.

16. World Economic Outlook Databases [Internet]. 2022 [cited April 13]. Available from: https://www.imf.org/en/Publications/SPROLLS/world-economic-outlook-databases#sort=%40imfdate%20descending.

17. Budget impact analysis of a population-based screening programme for colorectal cancer in Malaysia: technical report of a modelling study [Internet]. Centre for Public Health, Queen's University Belfast. 2022 [cited July 1]. Available from: https://pure.qub.ac.uk/en/publications/budget-impact-analysis-of-a-population-based-screening-programme-.

18. Malaysia Population Pyramid 2022 [Internet]. World Population Review. [cited Jan 21, 2022]. Available from: https://worldpopulationreview.com/countries/malaysia-population.

19. Demographic Statistics Factsheet [Internet]. Department Statistics of Malaysia. 2021 [cited Jan 21, 2022]. Available from: https://www.dosm.gov.my/v1/index.php?r=column/cthemeByCat&cat=430&bul_id=N05ydDRXR1BJWVlTdDY4TldHd253dz09&menu_id=L0pheU43NWJwRWVSZklWdzQ4TlhUUT09.

20. Medical Officer Grade UD41 [Internet]. Public Services Commission of Malaysia. [cited January 27, 2022]. Available from: https://www.spa.gov.my/spa/laman-utama/gaji-syarat-lantikan-deskripsi-tugas/ijazah-sarjana-phd/pegawai-perubatan-gred-ud41.

21. Medical laboratory technologist Grade U29 [Internet]. Public Services Commission of Malaysia. [cited January 27, 2022]. Available from: https://www.interactive.jpa.gov.my/ezskim/klasifikasi/perbekalanskim.asp?id_skim=3LU03.

22. Jaafar S, Noh KM, Muttalib KA, Othman NH, Healy J. Malaysia Health System Review. Asia Pacific Observatory on Health Systems and Policies; 2013.

23. Health Information and Quality Authority. Guidelines for the budget impact analysis of health technologies in Ireland. 2018.

24. M.Gray A, Clarke PM, Wolstenholme JL, Wordsworth S. Applied Methods of Cost-effectiveness Analysis in Health Care. Gray AM, Briggs A, editors. New York: Oxford University Press; 2011.

25. Ministry of Finance Malaysia. Budget 2022: RM32.4 billion allocation for MOH. Official Portal of Ministry of Finance Malaysia. 2021 [cited 2022 April 13]. Available from: https://www.mof.gov.my/portal/en/news/press-citations/budget-2022-rm32-4-billion-allocation-for-moh.

26. Leelahavarong P. Budget Impact Analysis. J Med Assoc Thai. 2014;97(Suppl. 5):S65-S71.

27. Orlewska E, Gulácsi L. Budget-Impact Analyses: A Critical Review of Published Studies. PharmacoEconomics. 2009;27(10):807-27.

28. Garattini L, van de Vooren K. Budget impact analysis in economic evaluation: a proposal for a clearer definition. The European Journal of Health Economics. 2011;12(6):499.

29. Risso-Gill I, Balabanova D, Majid F, Ng KK, Yusoff K, Mustapha F, et al. Understanding the modifiable health systems barriers to hypertension management in Malaysia: a multi-method health systems appraisal approach. BMC Health Services Research. 2015;15(1):254.

30. World Bank Open Data: GDP per capita [Internet]. World Bank. 2021 [cited March 9, 2023]. Available from: https://data.worldbank.org/indicator/NY.GDP.PCAP.CD.

31. Malaysia Toll Calculator – Google Maps with Tolls & Fuel [Internet]. TollGuru. 2023 [cited March 9, 2023]. Available from: https://tollguru.com/toll-calculator-malaysia.

For peer review only - http://bmjopen.bmj.com/site/about/guidelines.xhtml

Figure Legends and Tables

Table 1 Input assumptions used to estimate the population at each stage of the patient pathways

Table 2 Resources and unit costs

Table 3 Annual cost of proposed practice (i.e., CRC screening programme)

Figure 1 Patient pathways in 'usual care' practice - opportunistic screening for CRC

Figure 2 Patient pathway in population-based CRC screening programme

Figure 3 Results of multiple univariate sensitive analyses showing key factors that exert most influence the net budget impact

Supplementary material

Figure S1 Visual depiction of the budget impact cost calculator

Table S1 Net budget impact of CRC screening programme over 5-year timeframe, by state

Table S2 Sensitivity of the total budget impact of CRC screening programme to changes in each variable individually

For peer review only

Figure 1: Patient pathway in 'usual care' practice - opportunistic screening for CRC

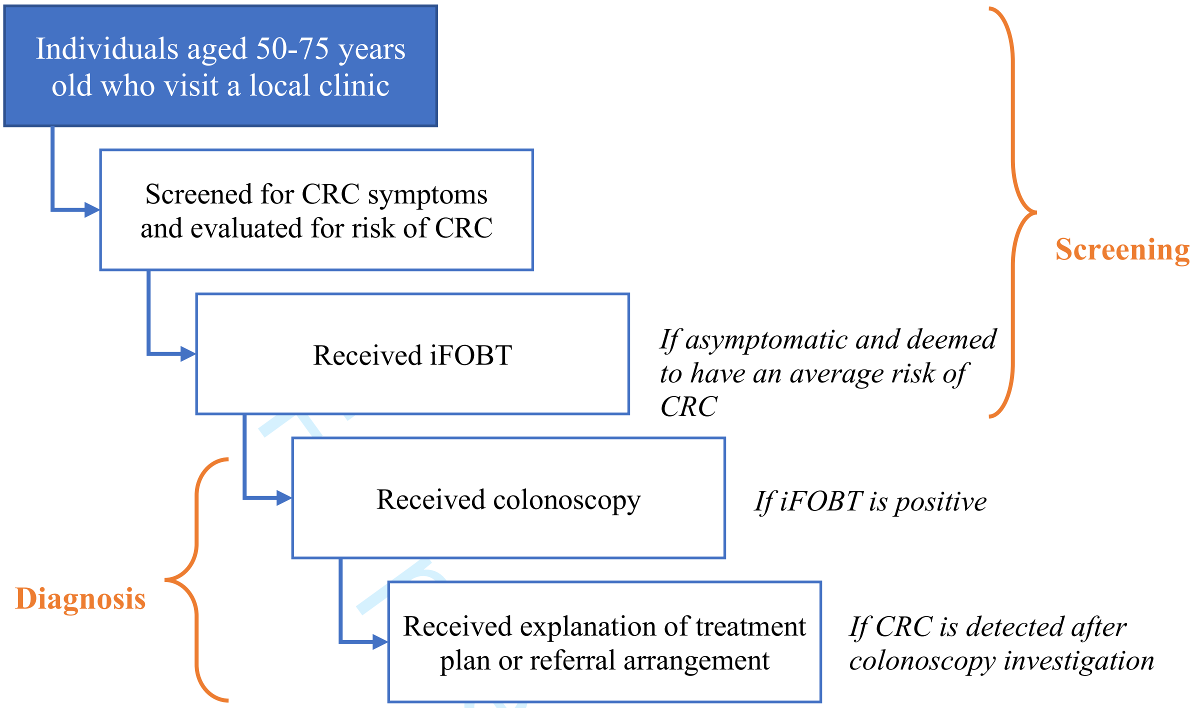

Figure 2: Patient pathway in population-based CRC screening programme

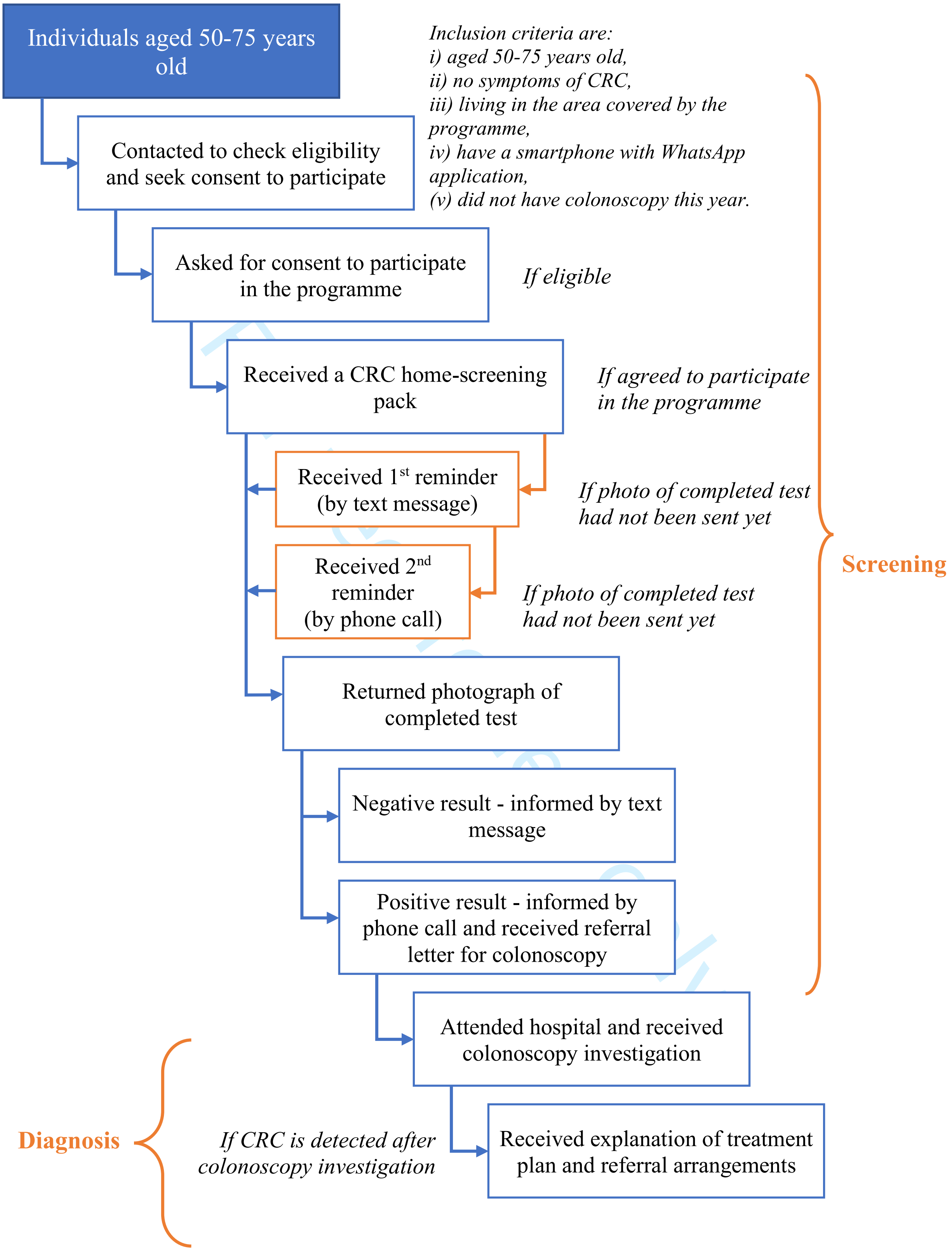

Figure 3: Results of multiple univariate sensitive analyses showing key factors that exert most influence the net budget impact

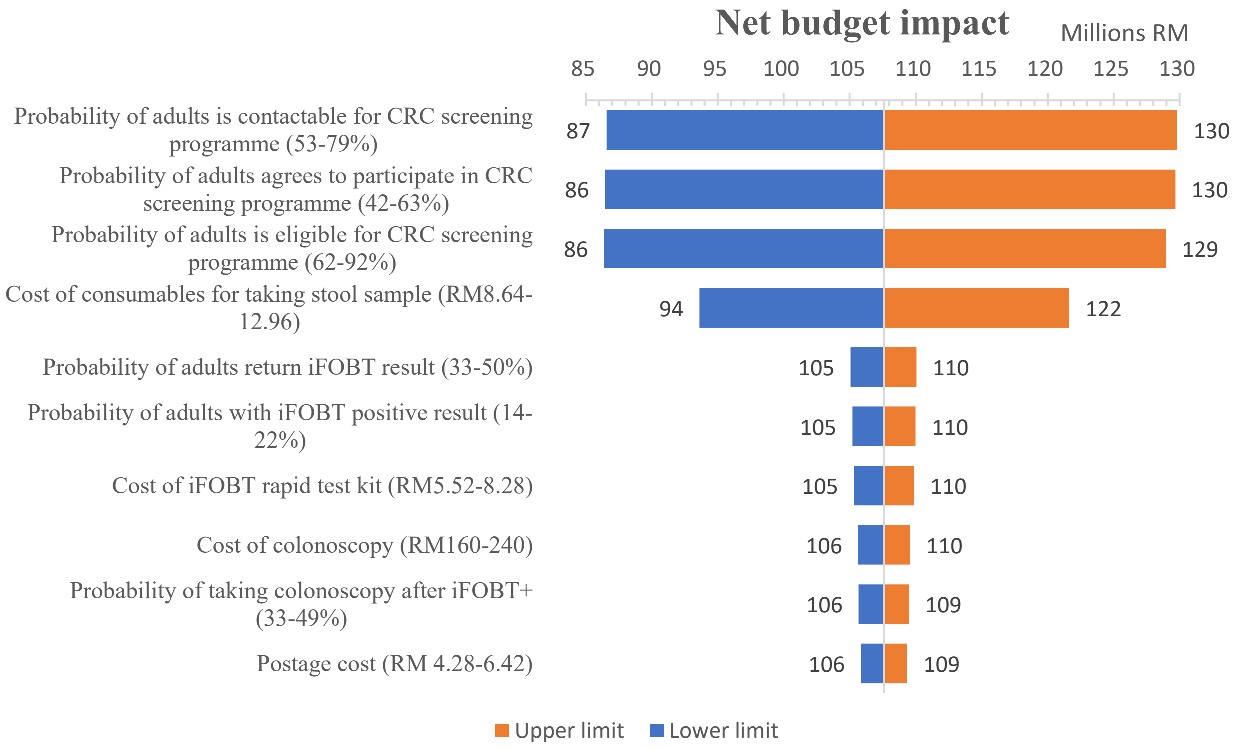

For peer review only

Supplementary material

Figure S1: Visual depiction of the budget impact cost calculator

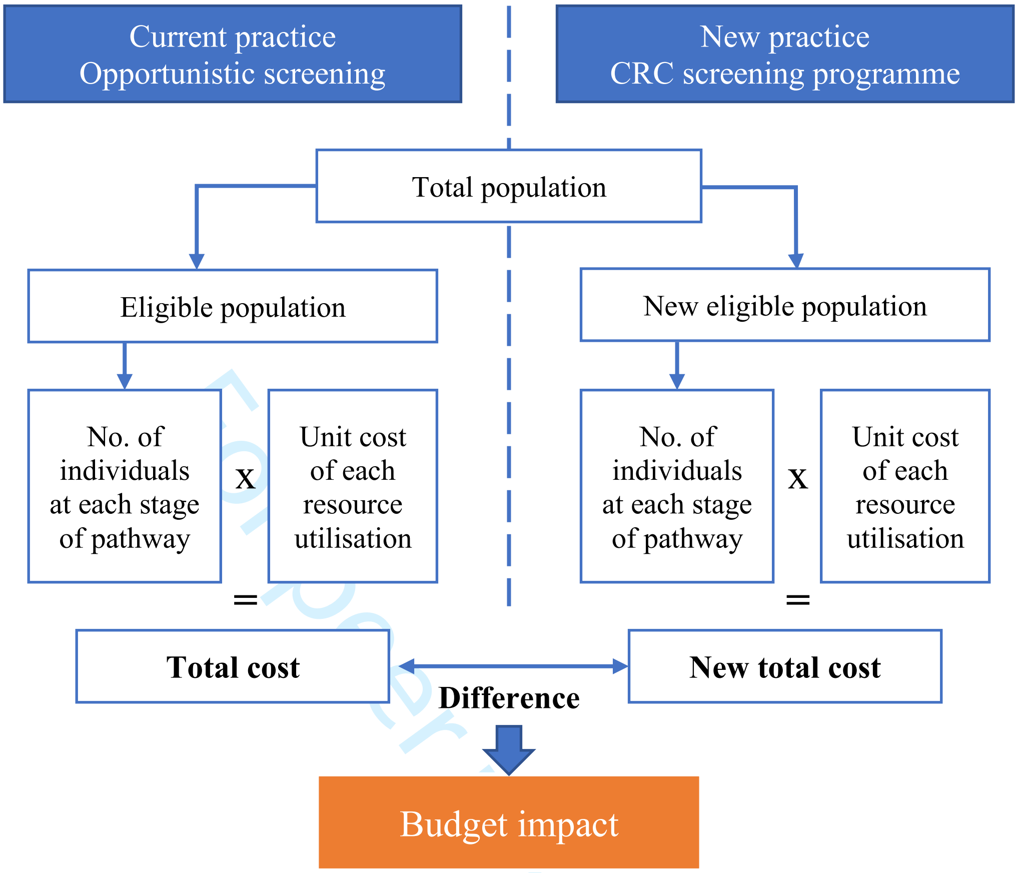

For peer review only

Supplementary material

Table S1: Net budget impact of CRC screening programme over 5-year timeframe, by state

Currency: Malaysian ringgit

State	Population	Year 1	Year 2	Year 3	Year 4	Year 5
**Johor**	3,822,800	12,597,041	13,791,897	14,986,752	16,181,607	17,376,462
**Kedah**	2,206,200	7,272,541	7,962,112	8,651,682	9,341,252	10,030,823
**Kelantan**	1,884,300	6,212,311	6,801,268	7,390,225	7,979,183	8,568,140
**Melaka**	934,700	3,084,637	3,376,787	3,668,937	3,961,087	4,253,238
**Negeri Sembilan**	1,141,000	3,764,122	4,120,753	4,477,384	4,834,016	5,190,647
**Pahang**	1,702,900	5,614,833	6,147,092	6,679,351	7,211,609	7,743,868
**Perak**	2,569,300	8,468,450	9,271,511	10,074,572	10,877,633	11,680,693
**Pulau Pinang**	1,767,100	5,826,301	6,378,626	6,930,951	7,483,276	8,035,601
**Sabah**	3,919,600	12,915,864	14,140,975	15,366,086	16,591,197	17,816,308
**Sarawak**	2,829,400	9,325,144	10,209,501	11,093,859	11,978,217	12,862,575
**Terengganu**	1,245,300	4,107,648	4,496,879	4,886,111	5,275,342	5,664,573
**Perlis**	253,500	841,028	920,262	999,496	1,078,730	1,157,964
**W.P. Kuala Lumpur**	1,790,100	5,902,043	6,461,557	7,021,071	7,580,585	8,140,099
**W.P. Labuan**	99,000	332,138	363,081	394,024	424,968	455,911
**W.P. Putrajaya**	97,100	325,886	356,236	386,585	416,935	447,284
**Nation-wide**	**32,676,786**	**107,631,959**	**117,845,422**	**128,058,885**	**138,272,349**	**148,485,812**

CRC: Colorectal cancer | W.P.: The Federal Territories (Malay: Wilayah Persekutuan)

Readers can convert from Malaysian Ringgit to their currency of interest (e.g., International Dollar, US Dollar, British Pound, Euro etc.) using the free web-based tool 'CCEMG – EPPI-Centre Cost Converter' (https://eppi.ioe.ac.uk/costconversion/default.aspx). This tool help adjusting estimates of cost expressed in one currency and price year to a specific target currency and price year.

For peer review only

Supplementary material

Table S2: Sensitivity of the total budget impact of CRC screening programme to changes in each variable individually

Baseline budget impact=RM107,631,959

Unit: Thousand Ringgit Malaysia

	Baseline value	Min value (-20% from baseline)	Max value (+20% from baseline)	Min budget impact	Max budget impact	Change
Probability of adults is contactable for CRC screening programme	66%	53%	79%	86,574	129,818	43,244
Probability of adults is included (eligible for CRC screening programme)	77%	62%	92%	86,386	128,950	42,564
Probability of adults agree to participate in CRC screening programme after being invited	52%	42%	63%	86,454	129,675	43,221
Probability of adults needing 1st reminder	79%	63%	94%	107,430	107,766	336
Probability of adults needing 2nd reminder	88%	70%	100%	107,535	107,643	108
Probability of adults return iFOBT result	42%	33%	50%	105,062	110,060	4,998
Probability of adults with iFOBT positive result	18%	14%	22%	105,204	109,988	4,784
Probability of adults taking colonoscopy after positive iFOBT	41%	33%	49%	105,673	109,493	3,820
Probability of adults with CRC detection after getting colonoscopy	4%	3%	5%	107,594	107,603	9
Cost to perform the screening (asking for symptoms, family history, referral)	5.58	4.47	6.70	107,633	107,567	-66
Cost of stool specimen processing	1.70	1.36	2.04	107,610	107,590	-20
Interpretation of results	2.79	2.23	3.35	107,617	107,583	-34
Cost to convey definitive diagnosis to patients (along with explaining treatment plan or referral etc.)	8.37	6.70	10.05	107,597	107,604	7
Contact eligible individuals - agreed to participate	0.98	0.79	1.18	107,285	107,926	641
Contact eligible individuals - rejected/excluded to participate	0.47	0.38	0.56	107,465	107,735	270
iFOBT rapid test kit (only the rapid test kit itself)	6.90	5.52	8.28	105,331	109,870	4,539
Print materials (instruction leaflet, explanatory statement)	1.10	0.88	1.32	107,238	107,962	724
Postage (stamp etc.)	5.35	4.28	6.42	105,840	109,360	3,520
Sending video through Whatapp	0.41	0.33	0.50	107,461	107,740	279
Sending reminder text message	0.41	0.33	0.50	107,490	107,710	220
Reminder call	0.28	0.22	0.33	107,536	107,661	125
Interpret the test kit result	1.70	1.36	2.04	107,366	107,832	466

For peer review only

Supplementary material

Unit: Thousand Ringgit Malaysia

	**Baseline value**	**Min value (-20% from baseline)**	**Max value (+20% from baseline)**	**Min budget impact**	**Max budget impact**	**Change**
Sending text message informing negative result	0.45	0.36	0.54	107,550	107,651	101
Call to inform positive result	0.41	0.33	0.50	107,590	107,611	21
Prepare and send referral letter	1.12	0.90	1.35	107,573	107,628	55
Follow up effort	6.73	5.39	8.08	107,503	107,698	195
Colonoscopy	200	160	240	105,638	109,564	3,926
Consumables – stool container, gloves, mask, plastic waste bag and disposal of materials from the test	10.80	8.64	12.96	93,612	121,641	28,029

For peer review only

ISPOR—The Professional Society for Health Economics and Outcomes Research

Budget Impact Analysis—Principles of Good Practice: Report of the ISPOR 2012 Budget Impact Analysis Good Practice II Task Force

Citation: Sullivan SD, Mauskopf JA, Augustovski F, Jaime Caro J, Lee KM, Minchin M, Orlewska E, Penna P, Rodriguez Barrios JM, Shau WY. Budget impact analysis-principles of good practice: report of the ISPOR 2012 Budget Impact Analysis Good Practice II Task Force. Value Health. 2014 Jan-Feb;17(1):5-14. doi: https://doi.org/10.1016/j.jval.2013.08.2291

Recommendations for Reporting Format

Section/topic	Guidance for reporting	Reported in section
**Introduction**		
Objectives	The objective of the BIA should be clearly stated and tied to the study perspectives	Introduction
Epidemiology and management of health problem	Present information about the prevalence and incidence of the particular disease, disease severity, disease progression, undiagnosed or undertreated cases, and risk factors pertinent to estimating the budget impact	Introduction
Clinical impact	Consist of a brief description of the eligible population and existing management options and their efficacy and safety that are relevant to the design of the study of the BIA	Introduction
Economic impact	Include a brief description of previous BIAs in the condition of interest for another intervention and condition-specific treatment patterns and cost of-care studies	Not applicable (No previous BIA)
**Study Design and Methods**		
Patient population	Specify the eligible population for the new intervention	Methods<br>Sub-section: Eligible population and input assumptions<br>Table 1
Intervention mix	Contain a detailed description of the use and characteristics of each intervention in the current intervention mix and in the expected intervention mix after the introduction of the new intervention	Methods<br>Sub-section: Health service under assessment and its comparator<br>Figure 1 and 2
Time horizon	Should be presented and the choice(s) justified	Methods<br>Sub-section: Perspective and time horizon
Perspective	Identify the BIAs' perspective(s), the cost categories included, and the intended audience	Methods<br>Perspective and time horizon

For peer review only - http://bmjopen.bmj.com/site/about/guidelines.xhtml

ISPOR—The Professional Society for Health Economics and Outcomes Research

Section/topic	Guidance for reporting	Reported in section
Analytic framework description	Complete description of the structure of the BIA cost calculator or condition-specific cohort or individual simulation model	Methods<br>Sub-section: Eligible population and input assumptions
Input data	Input values used for the reported analyses, including alternative scenarios, should be presented	Methods<br>Sub-section: Cost input and data sources<br>Table 2
Data sources	The sources of data inputs should be described in detail	Methods<br>Sub-section: Cost input and data sources<br>Table 2
Data collection	The methods and processes for any primary data collection and data abstraction tasks not reported elsewhere should be described and explained.	Not applicable (secondary data)
Analyses	A description of the calculations used to complete the BIA should be provided. The choice of all the scenarios presented in the results should be documented and justified.	Methods<br>Sub-section: Computing framework and base-case analysis under budget impact analyses
Uncertainty	Uncertainty analysis methods should be described and justified	Methods<br>Sub-sections: Uncertainty and scenario analyses under budget impact analyses
**Results**	The budget impact should be presented for each budget period over the time horizon. Both budget period resource use and costs should be presented. The estimates of resource use should be listed in a table that shows the change in use for each time period reported in the BIA<br><br>The results of the uncertainty analyses and scenarios analyzed should be described and presented in figures or tables	Results<br>Table 3<br><br>Results<br>Figure 2
**Conclusions and Limitations**	State the main conclusions on the basis of the results of the BIA.<br>Report the main limitations regarding key issues such as design aspects including off-label use and adherence assumptions and the completeness and quality of data inputs and sources.	Discussion<br><br>Conclusion
**Inclusion of Graphics and Tables**		
Figure of the analytical framework	Flow diagrams or other visual depictions of the cost calculator or condition-specific cohort or individual simulation model are recommended to be included with the analytical framework description.	Supplementary material<br>Figure S1

For peer review only - http://bmjopen.bmj.com/site/about/guidelines.xhtml

ISPOR—The Professional Society for Health Economics and Outcomes Research

Section/topic	Guidance for reporting	Reported in section
Table of assumptions	All the major assumptions should be listed in a tabular form	Table 1
Tables of inputs	All the input parameter values and their data sources and derivations should be presented in a tabular form	Table 2
Tables of outputs	All outputs should be presented in a tabular and/or graphical Form	Table 3
Schematic representation of uncertainty analyses	Diagrams such as Tornado diagrams should be included along with the text on the results of the scenario analyses	Figure 3
Appendices and References	The appendices may cover literature search strategies, evidence summaries, intermediate results (e.g., of individual Delphi panel rounds), and the names and addresses of participating experts and investigators, for example.	Reference

For peer review only - http://bmjopen.bmj.com/site/about/guidelines.xhtml

CHEERS 2022 Checklist

Topic	No.	Item	Location where item is reported
**Title**			
	1	Identify the study as an economic evaluation and specify the interventions being compared.	Title page, Page 1
**Abstract**			
	2	Provide a structured summary that highlights context, key methods, results, and alternative analyses.	Abstract, Page 1
**Introduction**			
**Background and objectives**	3	Give the context for the study, the study question, and its practical relevance for decision making in policy or practice.	Introduction, Line 65-69
**Methods**			
**Health economic analysis plan**	4	Indicate whether a health economic analysis plan was developed and where available.	Not applicable
**Study population**	5	Describe characteristics of the study population (such as age range, demographics, socioeconomic, or clinical characteristics).	Methods, Line 123-152, Table 1
**Setting and location**	6	Provide relevant contextual information that may influence findings.	Methods, Line 79-92
**Comparators**	7	Describe the interventions or strategies being compared and why chosen.	Methods, Line 79-121, Figure 1 and 2
**Perspective**	8	State the perspective(s) adopted by the study and why chosen.	Methods, Line 199-202
**Time horizon**	9	State the time horizon for the study and why appropriate.	Methods, Line 204-205
**Discount rate**	10	Report the discount rate(s) and reason chosen.	Methods, Line 205-206
**Selection of outcomes**	11	Describe what outcomes were used as the measure(s) of benefit(s) and harm(s).	Not applicable

Topic	No.	Item	Location where item is reported
**Measurement of outcomes**	12	Describe how outcomes used to capture benefit(s) and harm(s) were measured.	Not applicable
**Valuation of outcomes**	13	Describe the population and methods used to measure and value outcomes.	Not applicable
**Measurement and valuation of resources and costs**	14	Describe how costs were valued.	Methods, Line 159-196
**Currency, price date, and conversion**	15	Report the dates of the estimated resource quantities and unit costs, plus the currency and year of conversion.	Methods, Line 73-76
**Rationale and description of model**	16	If modelling is used, describe in detail and why used. Report if the model is publicly available and where it can be accessed.	Not applicable
**Analytics and assumptions**	17	Describe any methods for analysing or statistically transforming data, any extrapolation methods, and approaches for validating any model used.	Methods, Line 211-221, Figure S1 (Supplementary Material)
**Characterising heterogeneity**	18	Describe any methods used for estimating how the results of the study vary for subgroups.	Not applicable
**Characterising distributional effects**	19	Describe how impacts are distributed across different individuals or adjustments made to reflect priority populations.	Not applicable
**Characterising uncertainty**	20	Describe methods to characterise any sources of uncertainty in the analysis.	Methods, Line 224-229, Figure S2 (Supplementary Material)
**Approach to engagement with patients and others affected by the study**	21	Describe any approaches to engage patients or service recipients, the general public, communities, or stakeholders (such as clinicians or payers) in the design of the study.	Methods, Line 233-235
**Results**			
**Study parameters**	22	Report all analytic inputs (such as values, ranges, references) including uncertainty or distributional assumptions.	Table 1 and 2, Table S2 (Supplementary Material)
**Summary of main results**	23	Report the mean values for the main categories of costs and outcomes of interest and summarise them in the most appropriate overall measure.	Results, Line 239-274, Table 3

Topic	No.	Item	Location where item is reported
**Effect of uncertainty**	24	Describe how uncertainty about analytic judgments, inputs, or projections affect findings. Report the effect of choice of discount rate and time horizon, if applicable.	Results, Line 277-295, Figure 3
**Effect of engagement with patients and others affected by the study**	25	Report on any difference patient/service recipient, general public, community, or stakeholder involvement made to the approach or findings of the study	Not applicable
**Discussion**			
**Study findings, limitations, generalisability, and current knowledge**	26	Report key findings, limitations, ethical or equity considerations not captured, and how these could affect patients, policy, or practice.	Discussion
**Other relevant information**			
**Source of funding**	27	Describe how the study was funded and any role of the funder in the identification, design, conduct, and reporting of the analysis	End of manuscript
**Conflicts of interest**	28	Report authors conflicts of interest according to journal or International Committee of Medical Journal Editors requirements.	End of manuscript

From: Husereau D, Drummond M, Augustovski F, et al. Consolidated Health Economic Evaluation Reporting Standards 2022 (CHEERS 2022) Explanation and Elaboration: A Report of the ISPOR CHEERS II Good Practices Task Force. Value Health 2022;25. doi:10.1016/j.jval.2021.10.008

For peer review only - http://bmjopen.bmj.com/site/about/guidelines.xhtml
